# Inspection of the Grapevine BURP Superfamily Highlights an Expansion of *RD22* Genes with Distinctive Expression Features in Berry Development and ABA-Mediated Stress Responses

**DOI:** 10.1371/journal.pone.0110372

**Published:** 2014-10-16

**Authors:** José Tomás Matus, Felipe Aquea, Carmen Espinoza, Andrea Vega, Erika Cavallini, Silvia Dal Santo, Paola Cañón, Amparo Rodríguez-Hoces de la Guardia, Jennifer Serrano, Giovanni Battista Tornielli, Patricio Arce-Johnson

**Affiliations:** 1 Center for Research in Agricultural Genomics CSIC-IRTA-UAB-UB, Bellaterra, Barcelona, Spain; 2 Departamento de Genética Molecular y Microbiología, Pontificia Universidad Católica de Chile, Santiago, Chile; 3 Departamento de Ciencias Vegetales, Facultad de Agronomía e Ingeniería Forestal, Pontificia Universidad Católica de Chile, Santiago, Chile; 4 Department of Biotechnology, University of Verona, Verona, Italy; USDA-ARS, United States of America

## Abstract

The *RESPONSIVE TO DEHYDRATION 22* (*RD22*) gene is a molecular link between abscisic acid (ABA) signalling and abiotic stress responses. Its expression has been used as a reliable ABA early response marker. In Arabidopsis, the single copy *RD22* gene possesses a BURP domain also located at the C-terminus of USP embryonic proteins and the beta subunit of polygalacturonases. In grapevine, a *RD22* gene has been identified but putative paralogs are also found in the grape genome, possibly forming a large *RD22* family in this species. In this work, we searched for annotations containing BURP domains in the *Vitis vinifera* genome. Nineteen proteins were defined by a comparative analysis between the two genome predictions and RNA-Seq data. These sequences were compared to other plant BURPs identified in previous genome surveys allowing us to reconceive group classifications based on phylogenetic relationships and protein motif occurrence. We observed a lineage-specific evolution of the RD22 family, with the biggest expansion in grapevine and poplar. In contrast, rice, sorghum and maize presented highly expanded monocot-specific groups. The Vitis RD22 group may have expanded from segmental duplications as most of its members are confined to a region in chromosome 4. The inspection of transcriptomic data revealed variable expression of BURP genes in vegetative and reproductive organs. Many genes were induced in specific tissues or by abiotic and biotic stresses. Three *RD22* genes were further studied showing that they responded oppositely to ABA and to stress conditions. Our results show that the inclusion of RNA-Seq data is essential while describing gene families and improving gene annotations. Robust phylogenetic analyses including all BURP members from other sequenced species helped us redefine previous relationships that were erroneously established. This work provides additional evidence for *RD22* genes serving as marker genes for different organs or stresses in grapevine.

## Introduction

A plant's adaptive response to overcome any abiotic or biotic stress requires the initiation of various biochemical and physiological measures, which will ultimately allow the organism to survive. Many advances in understanding plant adaptation to abiotic stress have arisen from the study of the phytohormone abscisic acid (ABA), which accumulates under cold, salinity and drought [1]. Changes in its concentration can lead to a number of adaptations including stomatal closure [2], growth inhibition and senescence or flowering induction, all of which can be regulated at a transcriptional level [3]. These events comprise changes in the expression of hundreds of genes that are influenced by the degree, extent and rate of each stress [4].

Genes induced by water-deficit stress belong to different functional categories, as reviewed by Bray [5], such as cell signalling, rescue and detoxification, cell maintenance, pathogenesis-related functions, solute and water relocation. Several genes which respond to dehydration (named *RD*) have been isolated in *Arabidopsis thaliana* [6]. The *RESPONSIVE TO DEHYDRATION* 22 (*RD22*) protein is induced by an ABA-dependent signalling mechanism, requiring *de novo* protein synthesis [7]. The Arabidopsis MYC2 (also known as RD22-BP1) and MYB2 transcription factors bind cis-elements in the *RD22* promoter and cooperatively activate its transcription in response to drought, salinity and exposure to exogenous ABA [8].

The high and rapid inducible expression of *RD22* genes provides strong evidence for their involvement in stress tolerance. Over-expression of both AtMYC2 and AtMYB2, causes an ABA-hypersensitive phenotype, improves the osmotic-stress tolerance of transgenic plants and accelerates *RD22* expression [9]. Wang et al [10] showed that the soybean *Glycine max* GmRD22 protein could directly improve stress tolerance when overexpressed in rice and it was also able to interact with an apoplastic peroxidase linked to lignin biosynthesis and cell wall strengthening in response to salt stress. Wang et al [11] reported how the *Gossypium arboreum GaRDL1* gene was transactivated by the GLABRA1-like GaMYB2 and had a role in cotton fiber production. More recently, another cotton RD22 ortholog from *Gossypium hirsutum (GhRDL1)* was shown to interact with an α-expansin, promoting seed mass and fiber (seed trichome) length [12].

RD22 proteins possess a BURP domain, found at the C-terminus of several other plant proteins such as USP embryonic abundant and polygalacturonase proteins (BURP: BNM2, USP, RD22, PG1β). Members of the BURP superfamily share some primary structural features, subcellular localization patterns (e.g. cell wall matrix) and possible mechanistic similarities. BURP proteins possess different modules in addition to the BURP domain: a hypothetical transit peptide (N-terminal hydrophobic region), a short segment and a segment of repeated motifs that are unique to each family.

*BURP* genes have been recently genome-wide identified and related to abiotic stress tolerance in several plant species. In mangrove, four genes encoding BURP domain-containing proteins (*BgBDC1*, *2*, *3*, and *4*) were all induced by salt, ABA, and drought stress [13]. In rice, *OsBURP03*, *OsBURP05* and *OsBURP17* were induced by at least one abiotic stress treatment, with ABA-dependent and independent pathways involved [14]. Recently, *BURP* genes were identified in soybean [15], maize [16], sorghum [16] and poplar [17], showing that their expression is differentially responsive to ABA and ABA-related stress conditions.

Grapevine is an interesting model for studying drought and ABA signalling responses as the commercial production of this species is usually controlled by regulated-deficit irrigation regimes. In addition, fruit ripening in this species is associated with a short and rapid increase in ABA synthesis (reviewed by Kuhn et al [18]). A grape *RD22* gene was identified [19], which is constitutively expressed at low levels in all tissues. Nevertheless, its expression was induced by drought and salt stress, ABA and sugar. In addition to this gene, other reports have shown the possible existence of other *RD22* genes in grape. This evidence comes from microarray experiments in different grapevine organs, such as shoot tips [20], berries [21] and virus-infected leaves [22]. However, these studies focused on understanding global transcriptomic networks responding to stress, and individual genes were not isolated or characterized. Finally, no previous studies have assessed the dimension of the Vitis BURP domain superfamily. In this work, we characterized the grapevine BURP superfamily by conducting *in silico* phylogenetic and transcriptomic analyses. Some members from the RD22 family were isolated and their expression profiles were studied in different conditions for testing them as putative marker genes for organs or different stresses in grapevine.

## Methods

### Search for BURP-domain containing homologues in the grape genome

An approximately 230 amino acid consensus BURP domain sequence was obtained from the alignment of Arabidopsis and rice BURP-domain proteins. This consensus was used in a BLAT search to identify homologous gene models in the Genoscope Grape 8X Genome Browser [23] and CRIBI's 12X V1 prediction (http://genomes.cribi.unipd.it/). Since a variable number of gene models were obtained from each genome version, we compared these annotations with previously published RNA-Seq data [24], [25] with the use of alignment and contig assembly tools in Vector NTI v9 (Invitrogen). Nineteen proteins were defined and deduced by manual editing based on the Genoscope and CRIBI annotations, RNA-Seq data and the comparisons with corresponding expressed sequence tags and deduced protein sequences from paralogous genes.

### Phylogeny reconstruction and bootstrap analysis

Grape BURPs were aligned against the full predicted amino acid sequences of proteins belonging to Arabidopsis thaliana, Populus trichocarpa, Glycine max, Vicia faba, Gossypium hirsutum, Gossypium arboreum, Brassica napus, Zea mays, Sorghum bicolour and Oryza sativa. Alignments were performed using the MUSCLE algorithm-based AlignX module from MEGA5 software [26]. Phylogenetic trees were constructed using the Neighbour Joining Tree, Maximum Parsimony and Maximum Likelihood methods in MEGA5 and computed using the “p-distance” and “no difference” methods, with uniform rates among sites and partial deletion gap treatment. The trees obtained were graphed in MEGA5 and FigTree. Tree nodes were evaluated by bootstrap analysis for 100 replicates. For the construction of the complete BURP tree, the RD29 protein was used as outgroup.

### Identification of conserved protein motifs

The online MEME (Multiple EM for Motif Elicitation) Suite was employed to analyze the protein sequences of BURP members from different plant species with an expected value lower than 2×10^−30^ (http://meme.nbcr.net/; [27]). Seventy-three sequences were screened, excluding the Polygalacturonase family since it is the most divergent outside the BURP domain.

### Clustering analyses of transcriptomic data

As a first approach, the expression profiles of grapevine BURP genes were assessed in the global *V. vinifera* cv Corvina (clone 48) gene expression ATLAS (Nimblegen platform) of different organs at various developmental stages [28]. The expression data were analyzed using T-MeV v4.81 [29], The fluorescence intensity values of each transcript in all tissues/organs were calculated as log2 and normalized, based on the median center genes/rows adjustment in order to generate a clustered heat map such as the ones generated by Dal Santo et al [30].

Secondly, we searched for grape Affymetrix microarray public data in PLEXdb (Plant Expression Database) [31]. Probe sets corresponding to the putative *VvBURP* genes were identified by BLASTN, version 2.2.15 (e value <1e^-45^, see Table S1 for probe IDs). For each microarray experiment, raw data were normalized for further analysis. The CEL files were normalized with RMA (Robust Multi-Array Average) [32] using the affy R package [33]. For genes with more than one probe set, the median of the expression values was considered. To calculate the fold change in each experiment, normalized expression values of each experimental condition were compared with their control. In addition, with the aim of identifying *VvBURP* genes showing similar expression profiles, average-linkage hierarchical clustering was performed using the Cluster 2.11 software as described previously [34].

### Isolation of Vv*RD22* genes

The mRNA sequences (including partial 5′ and 3′UTR regions) of two Vv*RD22* genes were isolated. These were named Vv*RD22b* (*VvBURP18*) and Vv*RD22c* (*VvBURP06*). Their sequences were amplified from mature seed and green berry skin cDNAs, respectively, using the primers VvRD22b-5′utr (5′-TAGCTTTTGAGCTTGAGTCCTT-3′) and VvRD22b-3′utr (5′-GAATAACCCACATCTCCAGCC-3′) and VvRD22c-5′utr (5′-AGCAAGCAAAGGTTCCAGTT-3′) and VvRD22c-3′utr (5′-TTTCAGCATGCTTCAACAT-3′). PCR products were cloned into pTOPO-SD (Invitrogen). Six clones for each gene were sequenced using the universal M13 forward and reverse primers. Vv*RD22b* and Vv*RD22c* sequences were deposited in Genbank, with the accession numbers FJ869893 and FJ869894, respectively.

### Grapevine developmental samples

Reproductive grapevine organs (*Vitis vinifera* L. cv. Cabernet Sauvignon) were collected from a commercial vineyard in the Maipo Valley (Chile). Inflorescence clusters from two developmental stages (eight and twelve weeks post bud break, WPBB) were included. A total of nine grape clusters were sampled from three plants every two weeks throughout fruit development, beginning two-three weeks after fruit set (four weeks before véraison) and ending at eight weeks after véraison. Berries were immediately peeled and deseeded. Seeds and skins were frozen in liquid nitrogen, and stored at -80 °C until required for RNA extraction.

### ABA and salinity treatments in grapevine seedlings

Two month old Cabernet Sauvignon seedlings grown *in vitro* on Murashige-Skoog (MS) medium [35] were transferred to a hydroponic system using half-strength MS (1/2MS) medium supplemented with or without 100 µM ABA (Sigma). Seedlings were maintained in a culture chamber with a long-day cycle at 25 ± 1°C. After 12 h of treatment, leaves were collected and frozen in liquid nitrogen.

Cabernet Sauvignon nodal explants (young shoots with three nodes) were placed vertically on sterile MS media and propagated for a month in a growth chamber (23 ± 2°C; 16 h photoperiod), under three experimental conditions: MS with 3 mM (control), 100 mM or 200 mM NaCl. At the end of the experiment, samples were photographed, collected and frozen at -80°C until total RNA was extracted from complete plantlets. Three biological replicates for each experiment were performed.

### Virus infection and *Botrytis cinerea* inoculation

Healthy and virus infected *V. vinifera* cv. Cabernet Sauvignon plants were selected from an experimental field (P. Universidad Católica de Chile). For this, viral screening was carried out in leaf samples of the medial segment of main shoots. Leaf and fruit at maturation stage were frozen in liquid nitrogen and then stored at −80°C until RNA extraction. Virus detection was performed by RT-PCR. Ten viruses (the most prevalent grapevine viruses worldwide) were assayed in each sample using appropriate primers as described by Vega et al [36]: Grapevine Virus A (GVA), Grapevine virus B (GVB), Grapevine Fanleaf Virus (GFLV), Grapevine Fleck Virus (GFkV), Tomato Ringspot Virus (ToRSV), and Grapevine Leaf-Roll-Associated Viruses (GLRaV) 1, 2, 3, 4 and 7. After this analysis, negative plants for all tested viruses were considered as healthy, while only GLRaV3 positive plants were considered as infected plants for experimental purposes. Healthy and virus-infected plants were kept separately in the field with similar growth conditions and handling practices.

*Botrytis cinerea* B05.10 spores were grown as previously described by Mengiste et al [37]. Detached grapevine Cabernet Sauvignon fully expanded leaves and mature grapes were infected with a 50 µL drop containing 5×10^6^ spores/mL in water and control tissues were inoculated with 50 µL of water. Both inoculated and control tissues were placed over a wet filter paper in square petri dishes (leaves) or in 24-well plates (grapes) to maintain high humidity and kept in a Percival growth chamber at 21°C day and 18°C night temperatures. Samples were collected at 96 h post inoculation, frozen in liquid nitrogen and stored at −80°C until RNA extraction.

### Nucleic acid extraction and quantitative comparison of gene expression

Total RNA was isolated from all organs and treated tissues, according to the procedure of Reid et al [38] using a CTAB-Spermidine extraction buffer. For cDNA synthesis, one µg of total RNA was reverse transcribed with random hexamer primers using Superscript II™ First-strand Synthesis™ (Invitrogen) according to the manufacturer's instructions. Relative transcript quantification of *VvRD22-a*, *b* and *c* genes was achieved by RT-qPCR, using the SensiMix SYBR kit (Bioline) and the Mx3000P detection system (Stratagene) as described in the manufacturer's manual. Considering the high sequence similarity between their coding regions, primers for quantitative PCR analysis were designed to amplify 3′UTR fragments. The primers used for quantitative PCR were: qVvRD22aF (5′-GCACATCATTCGGTGTATCG-3′), qVvRD22aR (5′-GCAATGGGGTTTGAAGTATTA-3′), qVvRD22bF (5′-TGCCCGACCCAAAACCACTGCTTC-3′), qVvRD22bR (5′-GAATAACCCACATCTCCAGCC-3′), qVvRD22cF (5′-GTATTTCAACCTTCAGCACA-3′) and qVvRD22cR (5′-TTTCAGCATGCTTCAACAT-3′).

PCR conditions and standard quantification curves were conducted according to Matus et al [39]. Gene expression levels were normalized differentially for each experiment, against control sample or a specific developmental stage, in order to obtain a ΔCt for each gene. Amplification of the *UBIQUITIN1* [38], *GLYCERALDEHYDE PHOSPHATE DEHYDROGENASE (GPDH)* [36] and *ACTIN* [40] genes was used for calibrating gene expression. Experiments were performed with three biological replicates and three technical replicates. Reaction specificities were further confirmed with melting gradient dissociation curves, electrophoresis gels and cloning and sequencing of each PCR product. All data were statistically analyzed with MINITAB v14 software (Minitab Inc., PA, USA). One-Way ANOVA and Tukey's media comparison analyses were conducted. Statistical differences between means were based on p<0.05 values.

## Results and Discussion

### Genome-wide identification of BURP genes in grapevine

The genome of the near-homozygous PN40024 genotype of *V. vinifera* cv Pinot Noir was screened for BURP gene sequences. BURP domains possess conserved features: two N-terminal phenylalanine residues, two cysteine residues and four repeated cysteine-histidine motifs: CH-X(10)–CH–X(25–27)–CH–X(25–26)–CH, where X can be any amino acid [41]. A consensus BURP sequence was obtained from the alignment of previously-isolated genes from *Arabidopsis thaliana* and rice (*Oryza sativa*). This was later used as a BLASTP query against the 8X (Genoscope) and 12X V1 (CRIBI) genome assemblies, allowing the identification of over 20 BURP-like proteins. The number of annotations between both genome versions varied, requiring the use of further bioinformatic data for accurate gene discovery.

**Figure 1:**
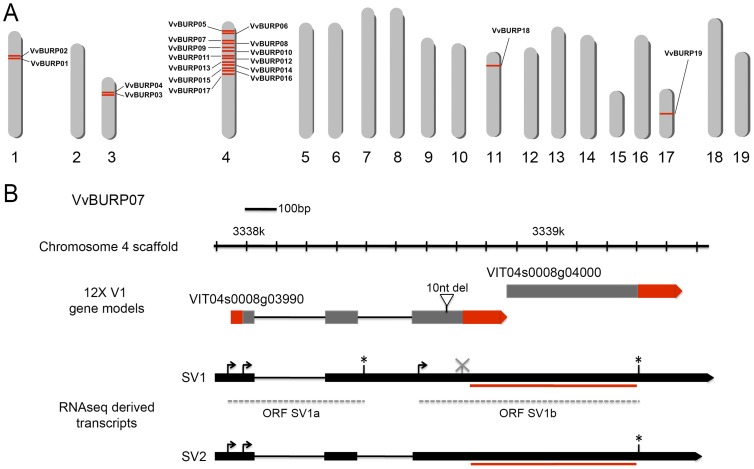
BURP domain-containing gene models in the grapevine genome. A) Gene positions in chromosomes 1, 3, 4, 11 and 17. An expanded cluster of closely related BURP genes (from the RD22 family) was found in chromosome 4. B) Assistance in gene model verification and re-annotation through the use of RNA-Seq data. The case for *VvBURP07* is shown as an example, where two incomplete BURP models from the 12X V1 prediction are no longer supported as independent units. The RNA-Seq data shows that a 10-nucleotide deletion was present in the 12X genome version, possibly corresponding to an error in the sequencing, as this deletion was not present in the 8X genome version. The two models actually represent one single annotation containing a complete BURP domain. SV: splicing variant. Red line under each SV represents the region coding for the BURP domain. Asterisks: stop codons in a corresponding ORF. Grey X: abolished stop codon in the SV ORF compared to the 12X annotation. For further inspection of this and other gene model re-annotations, see Table S1 and Figure S1.

As observed in gene models belonging to other protein families (e.g. MYB genes [39]), the *ab initio* prediction algorithm used to define gene annotations may split a single gene into two gene models or include intron sequences as exonic regions, among other annotation errors. To overcome these issues, we searched for mRNA sequences within a comprehensive RNA-Seq dataset from *Vitis vinifera* cv. Corvina [24], [25]. The careful inspection of these sequences supports the existence of 19 gene identities (Figure 1, Table S1). The chromosomal distribution of redefined grapevine BURP genes is presented in Figure 1A, showing that paralog genes are present in chromosomes 1, 3, 4, 11 and 17, with 13 of them in a cluster on chromosome 4. We compared the number of grapevine BURP genes to those found in other available angiosperm genomes. In soybean and rice, BURP families are composed of 16 and 17 members, respectively [14], [42], while in sorghum, maize and poplar there are 11, 15 and 18 members respectively [16], [17]. This data suggests that, even though the number of BURP genes between species is similar, the grape superfamily may have undergone a specific expansion of a particular group within chromosome 4. Family-specific expansions and diversification have been suggested previously, as in the case of genes related to wine characteristics [39] and grape expansins [30]. Additional molecular features of each BURP gene model and their predicted proteins are listed in Table S1, while DNA and protein alignments between the 8X, 12X V1 and RNA-Seq derived transcripts are shown in Figure S1. Nucleotide and amino acid polymorphisms may reflect cultivar differences between the reference genome and the RNA-Seq data (cv. Pinot Noir and cv. Corvina, respectively).

Previous comparisons between genome predicted sequences and available RNA-Seq data allowed the isolation of two sirtuin grape genes [43]. The use of such information has been important for building gene models *de novo* [44]. As an exemplifying case of the use of the RNA-Seq data for the re-interpretation of BURP gene model annotations, we show *VvBURP07* re-annotation in Figure 1B (also in Figure S1). The 12X V1 genome version denoted two independent gene models (VIT_04s0008g03990 and VIT_04s0008g04000), whose deduced amino acid sequences in fact represented incomplete BURP proteins. Two RNA-Seq derived transcripts (SV1 and SV2) allowed us to re-define and fuse these two gene models into one single BURP gene.

### Phylogenetic analyses of the grapevine BURP proteins

**Figure 2:**
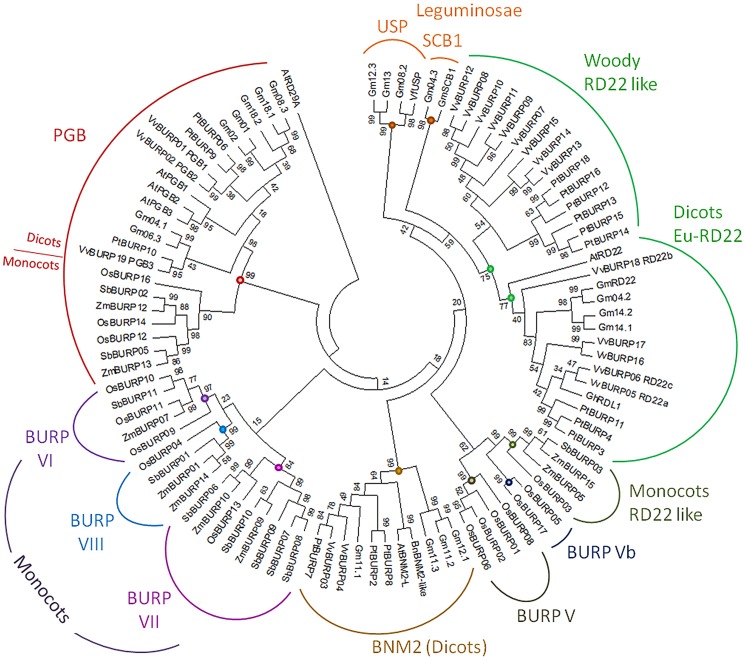
Phylogenetic tree of grapevine BURP proteins and their homologues in mono and dicotyledonous plant species. Highly resolutive NJ tree constructed under the “no differences” model, with uniform rates among sites and partial deletion gap treatment. At: *Arabidopsis thaliana*, Bn: *Brassica napus*, Gh: *Gossypium hirsutum*, Gm: *Glycine max*, Os: *Oryza sativa*, Pt: *Populus trichocarpa*, Sb: *Sorghum bicolour*, Vf: *Vicia faba*, Vv: *Vitis vinifera*, Zm: *Zea mays*. Protein IDs and descriptions for each gene are found in Table S1 and Table S2. Evolutionary distances are represented as amino acid substitutions per site. Clades with high bootstrap values (>75) that hold distinctive structural features are shown with coloured circles.

To gain insight into the relationships between the grape BURP genes, we first constructed a phylogenetic tree including the 19 grapevine proteins identified in this work based on their deduced amino acid sequences together with the five Arabidopsis BURP proteins (Figure S2 and S3). BURP proteins have been grouped into different categories according to each genome-wide study that has been performed so far. In dicotyledonous species they are classified in: 1) The beta subunit of polygalacturonases (PGβ); 2) Brassica BNM2-like proteins; 3) RD22 proteins and 4) USP seed embryo abundant proteins [45] [46] [47]. The RD22, PGβ and BNM2 groups were found in Arabidopsis and grape. The grape RD22 family is clearly expanded, but up to this point it was not possible to distinguish if any of these models belonged to a USP lineage. We therefore constructed a phylogenetic tree containing the entire grapevine BURP superfamily and additional proteins from all previously surveyed BURP families that represented different monocot and eudicotclades (list of proteins in Table S2). We found that the previous classifications were maintained, as no Arabidopsis or grape models were present in the USP family (Figure 2). In fact, this clade may only form part of leguminous genomes. Arabidopsis AtUSPL1 (AtBNM2-L) belongs to the BNM2 family rather than the USP family although it is localized, as is VfUSP, in cellular compartments essential for seed protein synthesis and storage [47].

Polygalacturonases are responsible for cell wall pectin degradation in expanding tissues throughout development. They have being largely studied in tomato fruit ripening and flower development. They are formed by a catalytic subunit and a highly glycosylated β-subunit (PGβ). PGβs are known to regulate polygalacturonase enzymatic activity and thermostability [48]. Our phylogenetic analysis showed that the PGβ family was more conserved than other families, with fewer variations in gene number (Figure 2). With the exception of soybean (seven genes), between two and three PGβs were found in grape, poplar, Arabidopsis, rice, maize and sorghum. These grouped separately within the monocot and eudicot lineages.

A high number of *RD22* genes were found in soybean, grape and poplar, but only members from the last two species formed an additional RD22-like cluster, more distantly related with AtRD22 and GhRDL1, the α-expansin-interacting RD22 protein from cotton [12]. This massive duplication of *RD22* genes appears to be a woody-specific event within eudicots. Possibly as for many other gene families, tandem and segmental duplications in different plant lineages may lead to species-dependent expansion of BURPs. The BURP superfamily has clearly undergone a monocot-specific expansion, leading to groups VI, VII and VIII (Figure 2). However, none of these appeared closely related to the RD22 family. The rice-exclusive Group V, here represented as subgroups V and Vb, shows a closer relationship with the monocot RD22 group, though while inspecting their protein sequences for domain distributions, this group lacks some of the motifs found in the RD22 family.

**Figure 3:**
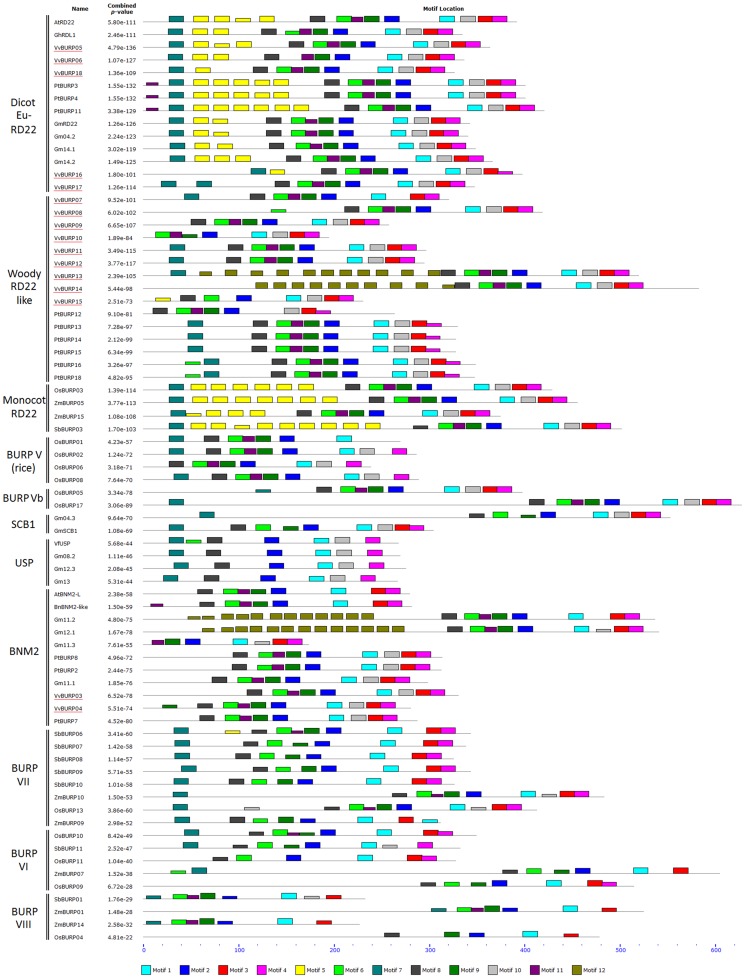
Motifs identified in the BURP proteins by MEME software. Polygalacturonase sequences were excluded from the analysis. Combined *p*-values are shown on the left of the figure.

To test and validate the phylogenetic relationships of Figure 2, we further analyzed the protein sequences of BURP members by using the online MEME Suite. Conserved motif identification with this tool allowed us to determine the number and consensus sequence of motifs constituting the BURP domain, as well as other motifs outside this region, some of which were specific to each group (Figure 3). As described by Ding et al [14], PGβs possess a series of exclusive motifs with few changes between species. For this reason, these sequences were not included in the analysis. The BURP domain generally consists of 8–9 motifs distributed mainly at the C-terminal of each protein. These motifs have been named differently in previous studies [14], [16]. Here we present a standardized nomenclature and sequence description, as seen in Figure S4 (taking the AtRD22 protein as an example). The BURP domain is composed of motifs m8, m6, m11, m9, m2, m1, m10, m3 and m4. Eudicot RD22 and Monocot RD22 groups possess these nine motifs. In addition, they possess motif m7 at the N-terminal region. Motif m5 was also found outside of the BURP domain, showing variable (from one to nine) repetitions. Most of the proteins from the woody RD22 like clade presented the nine motifs from the BURP domain and m7 at the N-terminus. Only one m5 repetition was found in the case of VvBURP15. In the case of VvBURP13 and VvBURP14, more than ten repetitions of an m12 motif were found. Group V and Vb presented only six to eight motifs from the BURP domain, m7 and no repeated m5 motifs. OsBURP05 and OsBURP17 shared a long sequence between m7 and the BURP domain but no motif was found here.

Groups V to VIII were previously described [14], [16], [17] but in all of these cases, classification errors were assumed, which may have lead to misinterpretations of the extent of each one of these monocot clades. In general, few species were considered in the construction of these phylogenetic trees, which could have caused the inconsistencies observed in the members of each clade. The most common way to estimate the reliability of a phylogeny is through a bootstrap test, as it estimates the consistency of each node. However, bootstrap values are absent in several phylogenetic trees [16], [17]. Additionally, we consider that poplar proteins that truly belong to the woody RD22 like cluster were erroneously assigned in the BURP V classification, as this group is monocot-specific [17]. Furthermore, two rice BURP genes (*OsBURP05* and *OsBURP17*) were mistakenly assigned as *VfUSP* like genes [16] and were later described as part of the RD22 group [14]; while we propose that both form part of group Vb as seen in Figure 2 and Figure 3. In our study, different combinations of models and algorithms were used to achieve the best phylogenetic relationships between BURP homologs. Another difference with previous studies was the use of the MUSCLE alignment algorithm instead of CLUSTALW, which applies a combination of both global and local alignments, re-optimizes the work as it progresses and performs better when sequence lengths are quite different even though they are from the same protein family [49]. Different trees were constructed and then compared by their bootstrap values. The best tree topologies were obtained from the Neighbor Joining Method under different substitution, rates and gap treatment models. The use of a large set of protein sequences from different species allowed us to construct a reliable tree, containing more accurate relationships. Finally, the use of protein domain search tools such as MEME allowed the validation of these associations.

### Global expression features of the BURP domain superfamily

**Figure 4:**
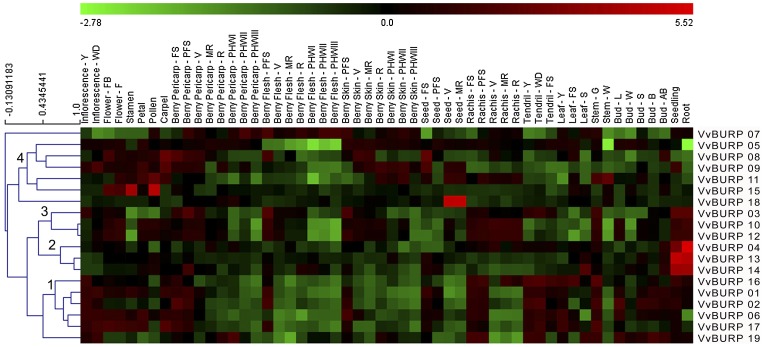
Expression features of the grapevine BURP superfamily. Log_2_ fluorescence intensity values from the *V. vinifera* cv. Corvina ATLAS were normalized based on the mean expression value of each gene in all tissues/organs. Different organs/tissues are displayed vertically above each column. Gene names are displayed to the right of each row. The colour scheme used to represent expression level is red/green: black boxes indicate a low variation in expression, green boxes indicate a fold decrease and red boxes indicate a fold increase with respect to the mean value. Samples and genes were hierarchically clustered based on the average Pearson's distance. Abbreviations after organ names indicate the developmental stage. FS, fruit set; PFS, post fruit set; V, véraison; MR, mid-ripening; R, ripening; PHWI, post-harvest withering (1st month); PHWII, post-harvest withering (2nd month); PHWIII, post-harvest withering (3rd month), Bud - L, latent bud; Bud - W, winter bud; Bud - S, bud swell; Bud - B, bud burst; Bud - AB, bud after burst; Inflorescence - Y, young inflorescence with single flowers separated; Inflorescence - WD, well developed inflorescence; Flower - FB, flowering begins; Flower - F, flowering; Tendril - Y, young tendril; Tendril - WD, well developed tendril; Tendril - FS, mature tendril; Leaf - Y, young leaf; Leaf - FS, mature leaf; Leaf - S, senescing leaf; Stem - G, green stem; Stem - W, woody stem.

The *V. vinifera* cv. Corvina gene expression ATLAS [28] was screened to characterize the expression profiles of the BURP superfamily. This microarray dataset comprises both vegetative and reproductive tissues, as well as berries that have undergone post-harvest withering for up to three months. We retrieved the fluorescence intensity values of the 19 BURP transcripts, generating a bi-clustered heat map (Figure 4). The family's expression profiles were divided in four main clusters: 1) mainly expressed in floral organs, buds, tendrils and early stages of rachis development, with a significant reduction of expression in berry (pericarp, skin and flesh) development from véraison (V) onwards; 2) high expression in seedlings and roots (*VvBURP04*, *VvBURP013* and *VvBURP014*); 3) expressed in seedlings, roots, berry tissues at early stages close to fruit set (FS or PFS) and throughout rachis development (*VvBURP10*, *VvBURP12* and more distantly *VvBURP03*) and 4) floral tissues and some cases of berry (pericarp, skin) development. Certain BURP genes were characterized by unique expression profiles in certain tissues. Within cluster 4, *VvBURP15* shows high expression in stamen and pollen just before flowering. The genes with most distant expressions were *VvBURP07* and *VvBURP18 (VvRD22b)*, with the latter mainly expressed in mature seed stages. In general terms, and with the exception of *VvBURP07*, the BURP superfamily shared a common repression profile throughout flesh morphogenesis.

Despite the contradictory hypotheses regarding PGβ roles in polygalacturonase function [50], [51], one certainty is that their expression is high in flower tissues and in ripening fruits. Grape *PGβ1* and *PGβ2* formed part of expression cluster 1. *PGβ3* (*VvBURP19*), closely related to cluster 1, had a similar expression profile in early flower development, tendrils, rachis and buds, but was not found in early stages of berry development. Instead it was expressed at pericarp ripening stages and in post harvest withering.

As suggested in all previous genome-wide studies, BURP genes may have a role in the response to abiotic stresses. However, their participation in biotic plant-pathogen interactions remains uncharacterized. As a second approach to study the expression profile of the BURP superfamily, we searched for public grape Affymetrix microarray data in PLEXdb [31]. One or two probe sets were found for some members of the family, either in the 16K or the *Vitis vinifera* GeneChip custom array (Table S1). As seen in Figure S5, different responses were found depending on the temporal extent of the stress and on the type of organ sampled. *VvBURP05* (*VvRD22a*) was induced at late stages after osmotic stress imposition in vegetative tissues (24 h after PEG or salt stress and 16 days after salt stress and water deficit, Figure S5A). *VvBURP12* was induced at different time points in response to osmotic stress. *VvBURP06*, *09* and *17* were mainly induced in heat stress recovered tissues and *VvBURP18* was only induced at 24 h after PEG treatment. In berry tissues (Figure S5B), *VvBURP18* (*VvRD22b*) was highly induced by water deficit in cv. Cabernet-Sauvignon berries while *VvBURP06* (*VvRD22c*) was repressed in these samples, especially towards the last ripening stages. On the other hand, expression of *VvBURP05* (*VvRD22a*) was relatively stable in these samples, except for its induction in one late ripening stage of water stressed cv. Chardonnay berries. *VvBURP06* was induced by heat. As seen in Figure S5C (biotic stress in vegetative tissues), Bois Noir phytoplasma infection generally repressed BURP gene expression with the exception of *VvBURP05, VvBURP12* and *VvBURP18*. This observation is in agreement with the fact that infection increased physical barriers to limit phytoplasma spread, with the repression of genes responsible for cell wall degradation (e.g. *VvPGβ1*) and the induction of genes involved in cell wall reinforcement [52]. *VvBURP05* and *VvBURP12* were induced in powdery mildew-resistant grapevines while *VvBURP06* was induced in downy mildew-infected samples. Finally, virus infections repressed the expression of *VvBURP06* and *VvBURP09*. All these findings revealed that the BURP superfamily is differentially regulated by a variety of biotic stresses.

### Analysis of three *VvRD22* genes with remarkably different expression profiles

As seen in the transcriptomic analysis, members of the grapevine RD22 family show several differences in their expression profiles, suggesting complementary or opposite roles in different organs. We further evaluated the expression of three Eudicot RD22 genes belonging to different expression clusters (Figure 4). These three closely-related genes (*VvBURP05*, *VvBURP06* and *VvBURP18*) were studied by means of Quantitative Real Time PCR (qPCR).

*VvBURP18* and *VvBURP06* were isolated and named *VvRD22b* and *VvRD22c*, respectively. The grapevine RD22 gene *VvBURP05*, previously isolated by Hanana et al [19], is referenced here as *VvRD22a*. The three putative protein products possess between 50 and 70% similarity in their complete protein sequences, and over 90% similarity in their BURP domains. The conserved sequences and motifs found in and around the BURP domain are shown in Figure S6. These three genes share the same distance between the CH dipeptides within the BURP domain (X5–CH–X10–CH–X25–CH–X25–CH–X8–W). VvRD22a, VvRD22b and VvRD22c harbour 3, 1 and 2 motif m5 repeats, respectively, while the Arabidopsis homolog possesses four repeats. Although RD22 proteins share the consensus repeat sequence described by Hanana et al [19] (VGVGKGTGVNVHAGKGKPGGGTT), the most different features correspond to the number of these repetitions outside the BURP domain.

**Figure 5:**
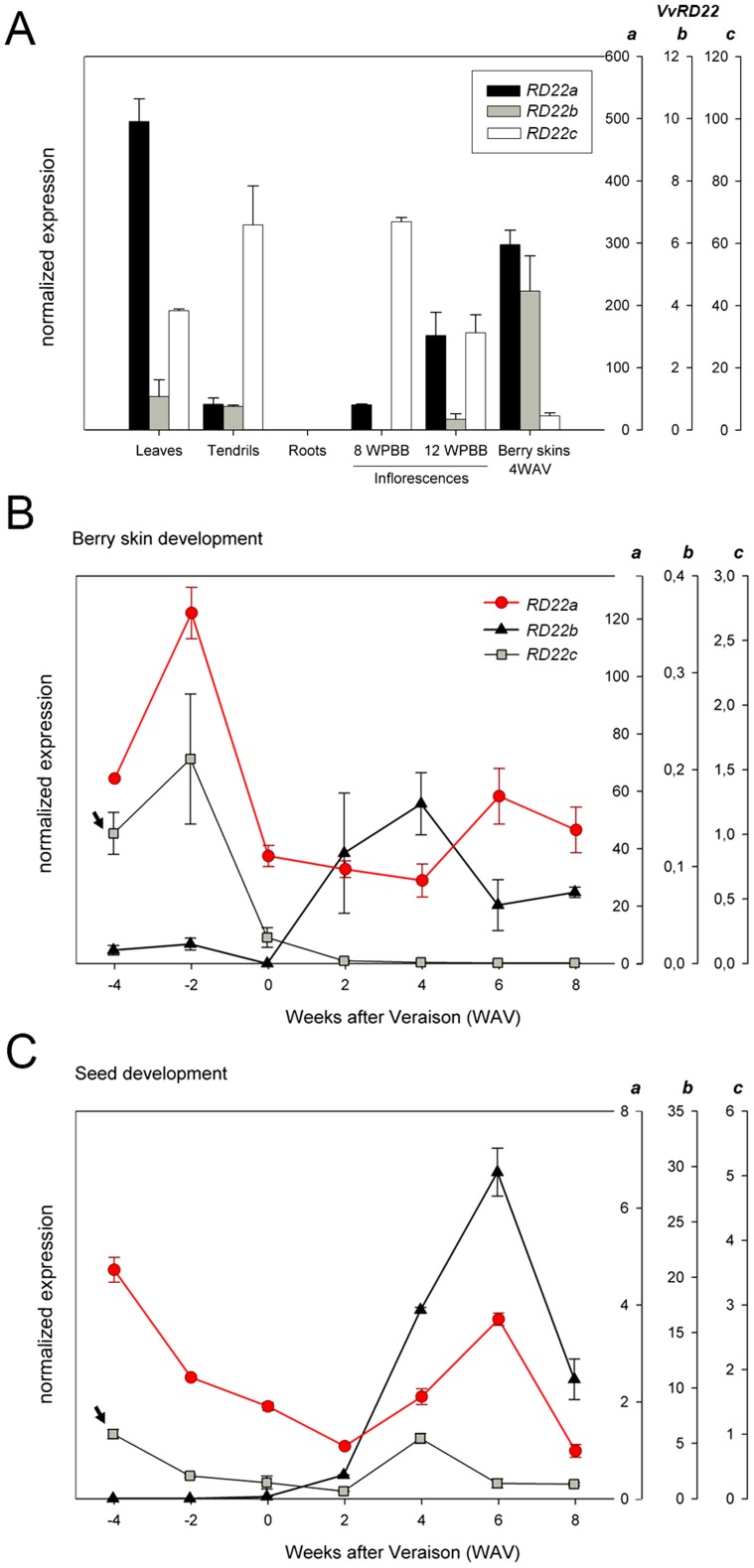
Expression profiles of RD22 genes in different grape organs and throughout berry development. A) Organ collection. All expression levels were normalized against *VvRD22b* expression in leaves. WPBB (weeks post-bud break) refers to inflorescence development. 4WAV: four weeks after véraison. Véraison: onset of ripening, when clusters are 30–50% coloured and the sugar concentration reaches 5° Brix (5% w/w soluble solids). Standard deviations (SD) are the result of three independent replicates. B–C) Expression of *VvRD22* genes in green and ripening stages of B) berry skin and C) seed development, beginning at -4WAV and ending at 8WAV. All expression levels were calibrated with the *VvUBIQUITIN1* housekeeping gene and normalized against *VvRD22c* expression at -4WAV. Stage 0WAV corresponds to véraison (onset of ripening). Standard deviations (SD) are the result of three independent replicates.

Our results revealed that these three grape *RD22* genes possess differential expression patterns in vegetative and reproductive organs (Figure 5A). None of these genes were expressed in roots, just like their homologues in Arabidopsis [6], soybean [42], and rice [14]. *VvRD22a* was the most expressed gene in all organs surveyed (seeds were not assessed). *VvRD22a* was highly expressed in leaves, late stages of inflorescence development and berry skins. Although *VvRD22b* was expressed at much lower levels than *VvRD22a*, it showed a four-fold induction in berry skins compared to leaves or other organs. Interestingly, *VvRD22c* showed the most distinct pattern of the three genes, being repressed in ripened berry skins when compared to all other organs.

We further analyzed the expression profiles of each gene throughout skin and seed development (Figure 5B–C). The expression of *VvRD22a* and *VvRD22c* was higher in berry skins before the onset of ripening (−4 and −2 weeks after véraison, WAV). From véraison onwards, the expression of *VvRD22c* was practically undetectable, while the expression of *VvRD22a* was maintained during the early ripening stages (0–4 WAV), slightly increasing again at 6 and 8 WAV (Figure 5B). On the other hand, *VvRD22b* was highly expressed after véraison, although at much lower levels than the other two RD22 genes. *VvRD22a* and *VvRD22c* were expressed differently in berry skins compared to seeds (Figure 5C). Their expression declined from −4 WAV to 2 WAV and slightly increased at 6 and 4 WAV, respectively, but both were much less expressed than *VvRD22b*. At 6 WAV, *VvRD22b* transcript abundance was 30 times higher than at véraison. The opposite expression profiles of *VvRD22b* and *VvRD22c* confirm the Affymetrix-derived data found in PLEXdb (Figure S5).

### *VvRD22* gene expression is differentially regulated by ABA and abiotic stress

ABA governs several berry ripening processes in grape (reviewed by Kuhn et al [18]). The level of this hormone in berries decreases after anthesis and then increases significantly at véraison, as measured by its concentration or by the expression of genes related to its synthesis [53], [54]. If ABA accumulates in berries at véraison, and if RD22 genes respond to ABA, as in diverse plant species [6], it is possible that the different expression patterns found for these *RD22* genes in berry tissues may be due to different concentrations of ABA in grape organs.

**Figure 6:**
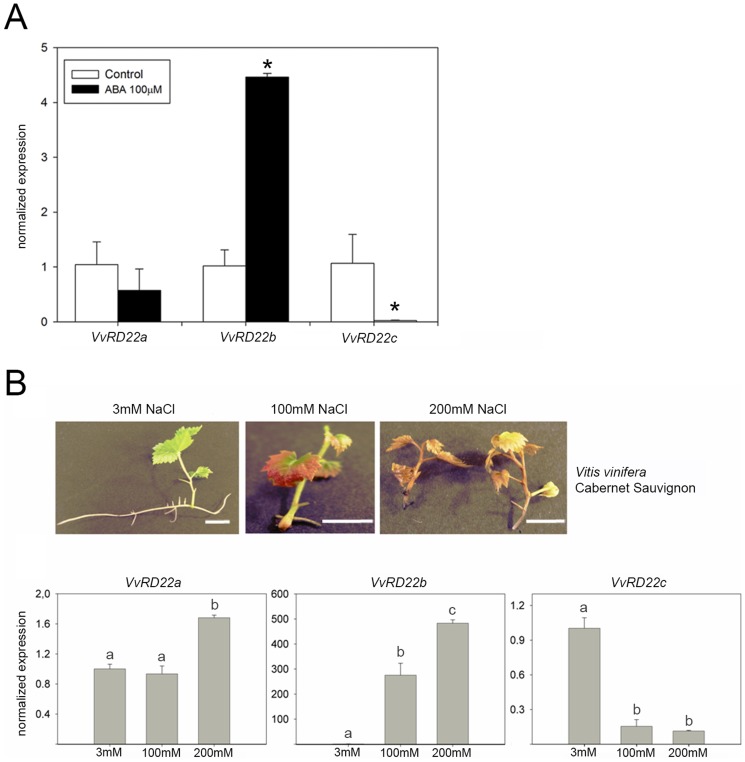
Expression of *VvRD22* genes in response to ABA and in grapevine plantlets exposed to salinity. A) Grapevine seedlings were treated with 100 µM ABA in a hydroponic solution. Gene expression was measured by RT-qPCR and data were normalized against the control (without ABA). B) Plantlets were grown for 30 days in standard MS or MS supplemented with 100 mM or 200 mM NaCl. Representative salinity stress phenotypes in Cabernet Sauvignon plantlets are shown in the upper part of the figure. Scale bar: 1 cm. qPCR expression levels were calibrated with the *VvGPDH* (for ABA samples) and *VvUBIQUITIN1* (for salt samples). Expression of each gene was normalized independently against the MS standard condition (3 mM NaCl). Standard deviations (SD) are the result of three independent replicates. Asterisks and lower case letters indicate significant differences between treatments as calculated by Tukey statistical analysis (p<0.05).

In order to evaluate whether ABA regulates the expression of grape *RD22* genes, seedlings cultured in hydroponic media were treated with this hormone and gene expression was analyzed 12 h after treatment. Figure 6A shows that after ABA treatment *VvRD22b* was induced by ABA, while *VvRD22c* was repressed; *VvRD22a* expression remained unaffected. These results confirm that both *VvRD22b* and *VvRD22c* genes are regulated by ABA. The expression of *VvRD22a* under our experimental condition seems not to be affected by ABA. However, Hanana et al [19] previously described that *VvRD22a* was indeed responsive to ABA, although those measurements were conducted in berry cell cultures, which may explain the difference in the ABA-responsiveness of this gene between these studies.

*RD22* genes have been described as reliable ABA reporter genes in response to drought and salinity. As a first attempt to characterize these *VvRD22* genes in response to abiotic stress, we studied *VvRD22* expression in leaves from plantlets subjected to high salt in their culture medium. For this, Cabernet Sauvignon nodal segments were grown in different NaCl concentrations (3 mM, 100 mM and 200 mM) until roots and/or leaves were visible and fully expanded. After several weeks, leaves had emerged from all explants while roots had developed only in MS containing 3 mM NaCl. At the end of the experiment, plantlets from the 100 mM NaCl treatment had a small radicle and high leaf anthocyanin accumulation, as a clear signal of stress in the plant (Figure 6B). When explants were grown on 200 mM NaCl, they became necrotic, indicating a strong toxic effect of high salt stress. Under this condition, roots did not develop.

As expected, the three grape *VvRD22* genes responded to salt treatments (Figure 6B), but with different tendencies. The expression of *VvRD22a* was only induced at 200 mM, while *VvRD22b* was strongly induced in both salt concentrations. The expression of *VvRD22c* was inhibited by salt stress. Considering that salt stress responses are mediated by ABA as described in many plant species, the contrasting behaviour of *VvRD22a/*b and *VvRD22*c in response to salt stress directly correlates with the ABA responsiveness observed for these *RD22* genes. In addition, Hanana et al [19] reported that *VvRD22a* was rapidly induced under salt stress in a tolerant variety when compared to a sensitive one.

In order to understand global responses to drought, Deluc et al [55] performed transcriptomic analyses during cv. Cabernet Sauvignon berry development comparing normal irrigated and water deficit conditions in field vineyards. The general profiles of RD22 expression derived from those data (which can be viewed in the PLEX database) correlated with our results obtained by qPCR. Taken together, there is thus strong evidence that the opposite expression patterns of *VvRD22b* and *VvRD22c* during fruit development may be due to opposite ABA responsiveness.

### Insights into the biotic stress responsiveness of grape *RD22* genes

**Figure 7:**
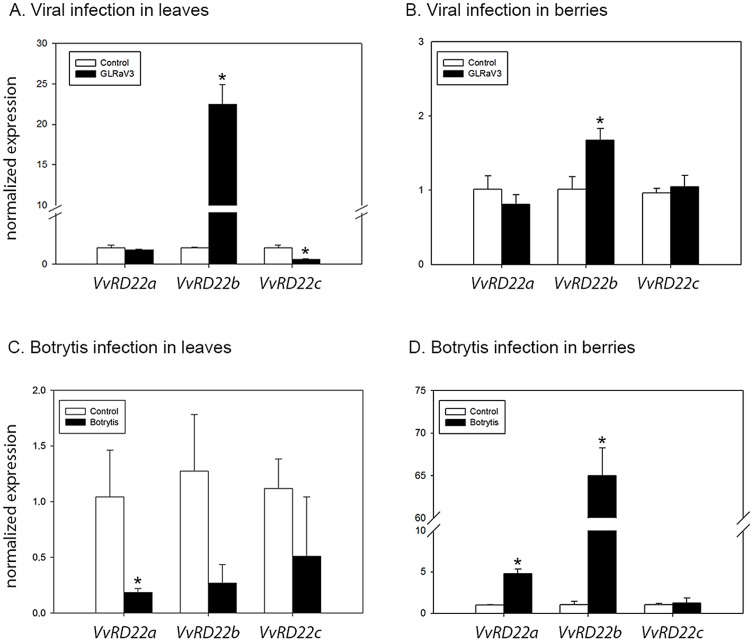
Expression of *VvRD22* genes in response to biotic stress. A) Vv*RD22* expression during viral infection in leaves. B) Vv*RD22* expression during viral infection in berries at maturity stage. Infected grapevine plants were positive for presence of GLRaV-3 virus only, while healthy plants were negative for all viruses tested. C) Expression analysis of Vv*RD22* genes in grapevine leaves inoculated with *Botrytis cinerea* mycelium. D) Expression analysis of Vv*RD22* genes in berries inoculated with *Botrytis cinerea* mycelium. Expression levels were calibrated with the *VvGPDH* and *VvACTIN* housekeeping genes for virus and Botrytis infected samples, respectively. Expression of each gene was normalized independently against its corresponding control. Standard deviations (SD) are the result of three independent replicates. Asterisks indicate significant differences between treatments as calculated by Tukey statistical analysis (p<0.05).

Recent evidence suggests the existence of a significant overlap between signalling networks that control abiotic stress tolerance and disease resistance. On the basis of experiments with exogenous application of ABA, inhibition of ABA biosynthesis and/or the use of ABA-deficient mutants, it has been shown that enhanced ABA levels correlate with increased pathogen susceptibility, and that a reduction below normal levels increases resistance to many pathogens [56], [57]. In order to evaluate the response of *VvRD22* genes to biotic stress, quantitative experiments were carried out in Grapevine Leaf Roll associated Virus 3 (GLRaV-3) infected and Botrytis inoculated grapevine leaves and fruits (Figure 7).

Under virus infection, the *UBIQUITIN* housekeeping gene does not behave homogenously between infected and non-infected samples [36]. For this reason, we used the *GPDH* gene as an internal control, as it does not show any significant variation between healthy and infected samples [22], [36]. The expression of *VvRD22a* did not change when comparing leaf or ripened berries from healthy and virus-infected plants (Figure 7A-B). The expression of *VvRD22b* was strongly induced by virus presence in leaves while it was slightly induced in fruits. *VvRD22c* was inhibited in virus-infected leaves whereas no change was observed in infected berries. Affymetrix-derived analysis confirmed the expression of *VvRD22c* found in infected leaves but not the induction of *VvRD22b* (Figure S5). In compatible infections, such as those established between virus and grapevine, it has been reported that stress-related genes are affected in infected leaves [22]. *VvRD22b* and *VvRD22c* presented an opposite regulation in virus infected-leaves, similar to our experiment of salt stress, suggesting a regulation of these genes during general stress conditions.

In the case of Botrytis infection, we selected *ACTIN* as reference for RT-qPCR [40]. Grapevine response to Botrytis in terms of *RD22* gene expression varied depending on the organ studied (Figure 7C–D). The expression of *VvRD22a* was negatively affected during Botrytis infection in leaves whilst the expression of *VvRD22b* and *VvRD22c* did not change significantly. Unlike in leaves, Botrytis infection in berries significantly triggered the expression of *VvRD22a* and *VvRD22b*, and again *VvRD22c* expression was not modified by the pathogen (Figure 7D). Together, these results confirm the differential regulation of *RD22* genes in a stimulus and tissue-specific manner, suggesting different/complementary functions in response to biotic stress. To our knowledge, this is the first report that associates *RD22* genes with biotic stresses.

The changes observed in viral and Botrytis infections could be a consequence of altered ABA levels following infection. ABA levels decreased in beans upon inoculation with rust [58]. In soybeans inoculated with Phytophthora, a decrease in ABA concentration occurred only during an incompatible interaction [59]. In contrast, viral infection in tobacco led to an increase in ABA levels [60]. These changes in hormone concentration were, however, modest compared to the dramatic changes in salicylate, jasmonate and/or ethylene production during pathogenesis, so these other hormones may also regulate RD22 expression. Recent findings suggest that there is an impact of viral diseases in ABA concentrations and in the expression of its biosynthetic genes, but also on the effects of the hormone in the accumulation of viruses [61], [62].

### Final remarks on the ABA responsiveness of RD22 genes

ABA-induced transcriptional activation is mediated by the presence of different cis-acting sequences in the promoter regions of stress responsive genes. The promoter of *AtRD22* is activated by RD22-BP1 (AtMYC2) and AtMYB2 transcription factors [8]. In Arabidopsis, these proteins interact and bind to a 67 bp region (located between -207 and −149 bp from the transcription initiation site) responsible for the desiccation-induced transcription of *RD22* [7]. It is possible that different combinations of regulatory MYB and MYC elements in the RD22 promoters could explain the distinct patterns of transcription that are observed. However, it was not possible to establish a direct correlation between the presence of these elements and the different expression responses of the three grape *RD22* genes studied here (*data not shown*). Additional factors have been recently related to the *RD22* gene. In Arabidopsis, the overexpression of MYB15 conferred ABA hypersensitivity, improved abiotic stress tolerance and increased RD22 expression [63]. The MYC (bHLH) transcription factor AtAIG1 was able to bind the DNA E-box sequence and when abolished, RD22 expression was reduced [64]. Finding new elements or new factors in grape may help to understand additional forms of regulation for these *RD22* genes. Post-translational regulatory mechanisms may also impact total abundance and/or activity of RD22 proteins in response to stress. As an example, the soybean GmRD22 protein is processed and localized in the apoplast, and the presence of its BURP domain is required for this localization [10].

Carra et al [65] found several grape BURP genes from chromosome 4 as potential targets of four small interfering RNAs (siRNA), suggesting that *VvRD22*-derived siRNAs were components of a regulatory mechanism based on RNA silencing in grapevine. Among these gene models, *VvRD22c* and *VvBURP16* were predicted *trans* targets of the siRNA id12, which in addition matched the *VvBURP07* gene model. Since we show that *VvRD22c* suffers more dramatic differences compared to *VvRD22a* and *VvRD22b* in terms of expression and stress responsiveness, this possible siRNA-induced control may be crucial for *VvRD22c* expression.

## Conclusions

This work reports the search for BURP genes in the *Vitis vinifera* genome. By using RNA-Seq data we re-defined those models originally found. Our study establishes new and improved phylogenetic classifications based on a big set of plant sequences and the search for protein motif occurrence. Our findings suggest that the expansion of *RD22* genes occurred with an increased rate within woody plant lineages. Although *RD22* genes have not been completely characterized, their expression is used as a direct indicator of an ABA-mediated response. Some evidence suggests that the specific induction of this gene in Arabidopsis (on a rhythmic basis) may be related to a process underlying memory functions of plants in response to ABA stimulus and light pulses in ABA-entrained plants [26]. From another perspective, *RD22* genes may be related to the maintenance of cell integrity, enhancing lignin polymerization and allowing plant cell to endure stressful conditions [10]. Each member of the grape *VvRD22* group presented a different expression pattern during organ development and in response to abiotic stress. Interestingly, we show they are induced still after long periods of stress and also in response to biotic stresses. Our expression analyses suggest that *VvRD22a* may respond to ABA while *VvRD22b* presumably responds to a higher degree. Nevertheless, these two genes show important differences in basal expression in non-stress conditions, where RD22a may have a predominant role in vegetative tissues. In contrast, *VvRD22c* was repressed by ABA, abiotic stresses and in berry development after the onset of ripening. These observations imply additional post-transcriptional regulation processes, such as the recently proposed siRNA mechanism, in addition to changes in protein processing and localization as described for other BURP superfamily members.

## Supporting Information

Figure S1 **DNA and protein alignments for each of the corresponding BURP gene models between the 8X and 12X genome versions and the RNA-Seq data.**
(PDF)

Figure S2 **Phylogenetic relationships of BURP homologues from Vitis and Arabidopsis.** Colour bar represents bootstrap values for each node. Protein IDs and descriptions for each Arabidopsis gene are found in Table S2. Evolutionary distances are represented as amino acid substitutions per site.
(TIF)

Figure S3 **Protein alignment between the grape and Arabidopsis BURP domains.** Residues highlighted in yellow correspond to conserved aminoacids from all grape and Arabidopsis proteins. Coloured bars correspond to highly conserved segments, which were found by MEME software (Figure S4).
(PDF)

Figure S4 **Schematic representation for AtRD22 motifs.** A) A comparison of motif identification between this work and those conducted by Ding et al [14] and Gan et al [16]. B) Consensus sequences for each of the motifs found by MEME software. Adjusted *p*-values for each motif are shown in parenthesis. Motif 12 is only present in VvBURP13, VvBURP14, Gm 11.2 and Gm 12.1.
(PNG)

Figure S5 **Heatmap clustering of** ***BURP*** **gene expressions for abiotic and biotic stress conditions in grapevine organs, obtained from the Affymetrix Plant Expression database (PLEXdb).** A-B) short and long-term abiotic stress conditions in A) vegetative organs and B) berry tissues, C) biotic stress in vegetative tissues. h: hours, d: days, P: pulp, Sd: seed, Sk: skin. R means recovery (25 °C for 5 h) after exposure to 45 °C. Numbers ranging from 31 to 38 represent stages of the Modified Eichhorn-Lorenz system for pericarp samples taken at different developmental stages (35: véraison). CH: cv. Chardonnay, IM: cv. Incrocio Manzoni, B: genotype Rpv1(+)Rpv2(-), C: genotype Rpv1(-)Rpv2(+), D: genotype Rpv1(-)Rpv2(-), CA: cv. Carmenere, CS: cv. Cabernet-Sauvignon.
(PNG)

Figure S6 **Protein alignment and motifs found in isolated grape RD22 proteins.** Residues highlighted in yellow correspond to conserved aminoacids. Orange segments represent the repeated motif 5 identified by MEME. A discontinuous box shows an incomplete motif 6 in RD22c.
(PNG)

Table S1 **Grapevine BURP genes identified in the PN40024 8X and 12X V1 predictions. **A) List of BURP genes. The final protein sequence for each gene was defined from the DNA and amino acid alignments between the 8X and 12X gene models, together with the RNA-Seq derived sequences (Figure S1). Fernandez et al. [66] studied a partial cDNA from VvBURP03 and named it BURP1 (EST Accession BQ799859). B) Other grapevine models in the Chromosome 4 cluster which are not BURP genes or do not possess a complete BURP domain. C) Sequences from RNA-Seq derived splicing variants (SV).
(XLS)

Table S2 **List of BURP domain genes from other genome-wide studied species used in our phylogenetic analyses (shaded in grey).** Gene models corresponding to truncated splicing variants and models with less than five motifs in the BURP domain were not included in the phylogenetic trees (in white).
(XLSX)
