# Supplementary material for: Inspection of the Grapevine BURP Superfamily Highlights an Expansion of RD22 Genes with Distinctive Expression Features in Berry Development and ABA-Mediated Stress Responses
Source: PLoS One. 2014 Oct 16;9(10):e110372. doi: 10.1371/journal.pone.0110372 (PMC4199669; doi:10.1371/journal.pone.0110372)
Supplement: Figure S1 — DNA and protein alignments for each of the corresponding BURP gene models between the 8X and 12X genome versions and the RNA-Seq data. (PDF) [file pone.0110372.s001.pdf]

VvBURP01
Protein alignment

```
                                   1                                                50
     GSVIVP00002686001      (1)   ---------------------MQVALGGAGEESGKPVAGENPFTPRASLL
     VIT_01s0127g00870      (1)   MHRQHKLLSHLFLIFLFSASFPNVALGGAGEESGKPVAGENPFTPRASLL
 VIT_01s0127g00870_SV1      (1)   --------------------------------------------------
             Consensus      (1)                         NVALGGAGEESGKPVAGENPFTPRASLL
                                   51                                              100
     GSVIVP00002686001     (30)   RYWNKQIGSGLPKSTFLLSKASPLSAVESATFTKLAAQNALSDNLPAFCK
     VIT_01s0127g00870     (51)   RYWNKQIGSGLPKSTFLLSKASPLSAVESATFTKLAAQNALSDNLPAFCK
 VIT_01s0127g00870_SV1      (1)   --------------------------------------------------
             Consensus     (51)   RYWNKQIGSGLPKSTFLLSKASPLSAVESATFTKLAAQNALSDNLPAFCK
                                   101                                             150
     GSVIVP00002686001     (80)   SANLLCFPDLGQSLEKHDASSNFAVYSNKNFTNYGTDGLGGTDAFTKYSE
     VIT_01s0127g00870    (101)   SANLLCFPDLGQSLEKHDASSNFAVYSNKNFTNYGTDGLGGTDAFTKYSE
 VIT_01s0127g00870_SV1      (1)   --------------------------------------------------
             Consensus    (101)   SANLLCFPDLGQSLEKHDASSNFAVYSNKNFTNYGTDGLGGTDAFTKYSE
                                   151                                             200
     GSVIVP00002686001    (130)   GVNLPVDAFKRYSRDSVGNNDKFDNYGREGNVVDQSFSGYGAGATGGSGE
     VIT_01s0127g00870    (151)   GVNLPVDAFKRYSRDSVGNNDKFDNYGREGNVVDQSFSGYGAGATGGSGE
 VIT_01s0127g00870_SV1      (1)   --------------------------------------------------
             Consensus    (151)   GVNLPVDAFKRYSRDSVGNNDKFDNYGREGNVVDQSFSGYGAGATGGSGE
                                   201                                             250
     GSVIVP00002686001    (180)   FKKYNEEVNVPNLRFNSYTDDGNGRQQSFTSYTNETNSGDESFSSYGKNG
     VIT_01s0127g00870    (201)   FKKYNEEVNVPNLRFNSYTDDGNGRQQSFTSYTNETNSGDESFSSYGKNG
 VIT_01s0127g00870_SV1      (1)   --------------------------------------------------
             Consensus    (201)   FKKYNEEVNVPNLRFNSYTDDGNGRQQSFTSYTNETNSGDESFSSYGKNG
                                   251                                             300
     GSVIVP00002686001    (230)   NGSPNEFTSYGSSSNVIGSTFTGYGETGNAANDTFKSYGFDGNVPENNFK
     VIT_01s0127g00870    (251)   NGSPNEFTSYGSSSNVIGSTFTGYGETGNAANDTFKSYGFDGNVPENNFK
 VIT_01s0127g00870_SV1      (1)   --------------------------------------------------
             Consensus    (251)   NGSPNEFTSYGSSSNVIGSTFTGYGETGNAANDTFKSYGFDGNVPENNFK
                                   301                                             350
     GSVIVP00002686001    (280)   KYGDGGNAGTDTFISYRDQSNVGDDSFKSYAKNSNSAEVDFVNYGKSFNE
     VIT_01s0127g00870    (301)   KYGDGGNAGTDTFISYRDQSNVGDDSFKSYAKNSNSAEVDFVNYGKSFNE
 VIT_01s0127g00870_SV1      (1)   --------------------------------------------------
             Consensus    (301)   KYGDGGNAGTDTFISYRDQSNVGDDSFKSYAKNSNSAEVDFVNYGKSFNE
                                   351                                             400
     GSVIVP00002686001    (330)   GTDTFAGYGKGATNHKINFKIYGVNNTFTDYAKKGISFSRYTNKSSETMT
     VIT_01s0127g00870    (351)   GTDTFAGYGKGATNHKINFKIYGVNNTFTDYAKKGISFSRYTNKSSETMT
 VIT_01s0127g00870_SV1      (1)   ------------------------------------------------MT
             Consensus    (351)   GTDTFAGYGKGATNHKINFKIYGVNNTFTDYAKKGISFSRYTNKSSETMT
                                   401                                             450
     GSVIVP00002686001    (380)   SMAVSGSSVNRWVEPGKFFRESMLKKGTVMPMPDIRDKMPKRSFLPRTIS
     VIT_01s0127g00870    (401)   SMAVSGSSVNRWVEPGKFFRESMLKKGTVMPMPDIRDKMPKRSFLPRTIS
 VIT_01s0127g00870_SV1      (3)   SMAVSGSSVNRWVEPGKFFRESMLKKGTVMPMPDIRDKMPKRSFLPRTIS
             Consensus    (401)   SMAVSGSSVNRWVEPGKFFRESMLKKGTVMPMPDIRDKMPKRSFLPRTIS
                                   451                                             500
     GSVIVP00002686001    (430)   SKFPFSTSKLEEMKKIFHAADNSSMEHMFTEALDDCERAPSKGETKRCVP
     VIT_01s0127g00870    (451)   SKFPFSTSKLEEMKKIFHAADNSSMEHMFTEALDDCERAPSKGETKRCVP
 VIT_01s0127g00870_SV1     (53)   SKLPFSTSKLEEMKKIFHAADNSSMEHMFTEALDDCERAPSKGETKRCVP
             Consensus    (451)   SKFPFSTSKLEEMKKIFHAADNSSMEHMFTEALDDCERAPSKGETKRCVP
                                   501                                             550
     GSVIVP00002686001    (480)   SIEDMIDFATTVLGRNVVVRTTQSVEGSKQNLMIGSVKGINGGQVTKSVS
     VIT_01s0127g00870    (501)   SIEDMIDFATTVLGRNVVVRTTQSVEGSKQNLMIGSVKGINGGQVTKSVS
 VIT_01s0127g00870_SV1    (103)   SIEDMIDFATTVLGRNVVVRTTQSVEGSKQNVMIGSVKGINGGQVTKSVS
             Consensus    (501)   SIEDMIDFATTVLGRNVVVRTTQSVEGSKQNLMIGSVKGINGGQVTKSVS
                                   551                                             600
     GSVIVP00002686001    (530)   CHQSLFPYLLYYCHSVPKVRVYEADLLDPKTKANVNHGVAICHLDTSDWS
     VIT_01s0127g00870    (551)   CHQSLFPYLLYYCHSVPKVRVYEADLLDPKTKANVNHGVAICHLDTSDWS
 VIT_01s0127g00870_SV1    (153)   CHQSLFPYLLYYCHSVPKVRVYEADLLDPKTKANVNHGVAICHLDTSDWS
             Consensus    (551)   CHQSLFPYLLYYCHSVPKVRVYEADLLDPKTKANVNHGVAICHLDTSDWS
```

```
                                601                             633
     GSVIVP00002686001  (580) AGHGAFVALGSGPGRIEVCHWIFENDMTWTIAD
     VIT_01s0127g00870  (601) AGHGAFVALGSGPGRIEVCHWIFENDMTWTIAD
 VIT_01s0127g00870_SV1  (203) AGHGAFVALGSGPGRIEVCHWIFENDMTWTIVD
             Consensus  (601) AGHGAFVALGSGPGRIEVCHWIFENDMTWTIAD
```

VvBURP01

DNA alignment. Black box: Translation start site. Red box: insertion/deletion in sequence

```
                                1                                                50
VIT_01s0127g00870_CRIBI     (1) TGAAGGAGATAGAGAGAGAAATGCATAGACAACATAAGCTTTTGAGCCAT
  VIT_01s0127g00870_SV1     (1) --------------------------AGACAACATAAGCTTTTGAGCCAT
              Consensus     (1)                           AGACAACATAAGCTTTTGAGCCAT
                                51                                              100
VIT_01s0127g00870_CRIBI    (51) CTCTTTCTCATTTTCTTATTCTCAGCTTCGTTTCCCAATGTTGCTTTGGG
  VIT_01s0127g00870_SV1    (25) CTCTTTCTCATTTTCTT--TCTCAGCTTCGTTTCCCAATGTTGCTTTGGG
              Consensus    (51) CTCTTTCTCATTTTCTT  TCTCAGCTTCGTTTCCCAATGTTGCTTTGGG
                                101                                             150
VIT_01s0127g00870_CRIBI   (101) CGGAGCCGGTGAGGAATCGGGCAAACCGGTGGCTGGAGAAAACCCATTCA
  VIT_01s0127g00870_SV1    (73) CGGAGCCGGTGAGGAATCGGGCAAACCGGTGGCTGGAGAAAACCCATTCA
              Consensus   (101) CGGAGCCGGTGAGGAATCGGGCAAACCGGTGGCTGGAGAAAACCCATTCA
                                151                                             200
VIT_01s0127g00870_CRIBI   (151) CACCCAGAGCGTCTTTGCTTCGGTATTGGAATAAGCAGATCGGCAGTGGC
  VIT_01s0127g00870_SV1   (123) CACCCAGAGCGTCTTTGCTTCGGTATTGGAATAAGCAGATCGGCAGTGGC
              Consensus   (151) CACCCAGAGCGTCTTTGCTTCGGTATTGGAATAAGCAGATCGGCAGTGGC
                                201                                             250
VIT_01s0127g00870_CRIBI   (201) TTGCCAAAGTCCACCTTTCTTCTCTCCAAGGCATCCCCATTGAGCGCGGT
  VIT_01s0127g00870_SV1   (173) TTGCCAAAGTCCACCTTTCTTCTCTCCAAGGCATCCCCATTGAGCGCGGT
              Consensus   (201) TTGCCAAAGTCCACCTTTCTTCTCTCCAAGGCATCCCCATTGAGCGCGGT
                                251                                             300
VIT_01s0127g00870_CRIBI   (251) TGAGTCGGCCACCTTTACTAAACTTGCCGCTCAAAATGCGCTTTCCGACA
  VIT_01s0127g00870_SV1   (223) TGAGTCGGCCACCTTTACTAAACTTGCCGCTCAAAATGCGCTTTCCGACA
              Consensus   (251) TGAGTCGGCCACCTTTACTAAACTTGCCGCTCAAAATGCGCTTTCCGACA
                                301                                             350
VIT_01s0127g00870_CRIBI   (301) ATCTGCCGGCGTTCTGTAAGTCTGCGAACCTGCTCTGTTTTCCCGATTTG
  VIT_01s0127g00870_SV1   (273) ATCTGCCGGCGTTCTGTAAGTCTGCGAACCTGCTCTGTTTTCCCGATTTG
              Consensus   (301) ATCTGCCGGCGTTCTGTAAGTCTGCGAACCTGCTCTGTTTTCCCGATTTG
                                351                                             400
VIT_01s0127g00870_CRIBI   (351) GGGCAGAGCTTGGAGAAGCATGACGCTAGTTCCAACTTCGCCGTGTATTC
  VIT_01s0127g00870_SV1   (323) GGGCAGAGCTTGGAGAAGCATGACGCTAGTTCCAACTTCGCCGTGTATTC
              Consensus   (351) GGGCAGAGCTTGGAGAAGCATGACGCTAGTTCCAACTTCGCCGTGTATTC
                                401                                             450
VIT_01s0127g00870_CRIBI   (401) GAACAAGAATTTCACCAATTACGGAACGGATGGACTCGGGGGAACGGACG
  VIT_01s0127g00870_SV1   (373) GAACAAGAATTTCACCAATTACGGAACGGATGGACTCGGGGGAACGGACG
              Consensus   (401) GAACAAGAATTTCACCAATTACGGAACGGATGGACTCGGGGGAACGGACG
                                451                                             500
VIT_01s0127g00870_CRIBI   (451) CCTTCACAAAATACTCGGAGGGGGTCAATCTTCCTGTCGACGCATTCAAG
  VIT_01s0127g00870_SV1   (423) CCTTCACAAAATACTCGGAGGGGGTCAATCTTCCTGTCGACGCATTCAAG
              Consensus   (451) CCTTCACAAAATACTCGGAGGGGGTCAATCTTCCTGTCGACGCATTCAAG
                                501                                             550
VIT_01s0127g00870_CRIBI   (501) CGCTATAGCCGTGACTCCGTCGGCAACAATGATAAATTCGATAATTACGG
  VIT_01s0127g00870_SV1   (473) CGCTATAGCCGTGACTCCGTCGGCAACAATGATAAATTCGATAATTACGG
              Consensus   (501) CGCTATAGCCGTGACTCCGTCGGCAACAATGATAAATTCGATAATTACGG
                                551                                             600
VIT_01s0127g00870_CRIBI   (551) CCGCGAGGGCAACGTCGTGGATCAGAGCTTCAGCGGCTACGGTGCCGGGG
  VIT_01s0127g00870_SV1   (523) CCGCGAGGGCAACGTCGTGGATCAGAGCTTCAGCGGCTACGGTGCCGGGG
              Consensus   (551) CCGCGAGGGCAACGTCGTGGATCAGAGCTTCAGCGGCTACGGTGCCGGGG
                                601                                             650
VIT_01s0127g00870_CRIBI   (601) CTACCGGCGGCTCTGGAGAGTTCAAGAAGTACAACGAAGAGGTTAATGTT
  VIT_01s0127g00870_SV1   (573) CTACCGGCGGCTCTGGAGAGTTCAAGAAGTACAACGAAGAGGTTAATGTT
              Consensus   (601) CTACCGGCGGCTCTGGAGAGTTCAAGAAGTACAACGAAGAGGTTAATGTT
                                651                                             700
VIT_01s0127g00870_CRIBI   (651) CCCAACCTTCGATTCAACTCCTACACAGACGATGGCAATGGCCGTCAACA
  VIT_01s0127g00870_SV1   (623) CCCAACCTTCGATTCAACTCCTACACAGACGATGGCAATGGCCGTCAACA
              Consensus   (651) CCCAACCTTCGATTCAACTCCTACACAGACGATGGCAATGGCCGTCAACA
                                701                                             750
VIT_01s0127g00870_CRIBI   (701) ATCATTCACGAGTTACACAAATGAGACCAATTCTGGAGATGAGTCCTTCA
  VIT_01s0127g00870_SV1   (673) ATCATTCACGAGTTACACAAATGAGACCAATTCTGGAGATGAGTCCTTCA
              Consensus   (701) ATCATTCACGAGTTACACAAATGAGACCAATTCTGGAGATGAGTCCTTCA
```

```
                                  751                                               800
VIT_01s0127g00870_CRIBI    (751)  GTAGCTACGGCAAGAACGGCAACGGATCTCCGAACGAGTTCACGAGTTAC
  VIT_01s0127g00870_SV1    (723)  GTAGCTACGGCAAGAACGGCAACGGATCTCCGAACGAGTTCACGAGTTAC
              Consensus    (751)  GTAGCTACGGCAAGAACGGCAACGGATCTCCGAACGAGTTCACGAGTTAC
                                  801                                               850
VIT_01s0127g00870_CRIBI    (801)  GGCAGCAGCTCCAACGTCATCGGCTCCACGTTCACCGGCTACGGTGAAAC
  VIT_01s0127g00870_SV1    (773)  GGCAGCAGCTCCAACGTCATCGGCTCCACATTCACAGGCTACAGTGAAAC
              Consensus    (801)  GGCAGCAGCTCCAACGTCATCGGCTCCAC TTCAC GGCTAC GTGAAAC
                                  851                                               900
VIT_01s0127g00870_CRIBI    (851)  CGGCAATGCCGCCAACGATACGTTCAAGAGCTACGGCTTTGACGGCAACG
  VIT_01s0127g00870_SV1    (823)  CGGCAACGCCGCCAACGATACGTTCAAGAGCTACGGCTTTGACGGCAACG
              Consensus    (851)  CGGCAA GCCGCCAACGATACGTTCAAGAGCTACGGCTTTGACGGCAACG
                                  901                                               950
VIT_01s0127g00870_CRIBI    (901)  TGCCAGAGAACAACTTCAAGAAATACGGTGATGGTGGTAATGCGGGGACC
  VIT_01s0127g00870_SV1    (873)  TGCCAGAGAACAATTTCAAGAACTACGGTGATGGTGGTAATGCGGGGACC
              Consensus    (901)  TGCCAGAGAACAA TTCAAGAA TACGGTGATGGTGGTAATGCGGGGACC
                                  951                                              1000
VIT_01s0127g00870_CRIBI    (951)  GATACTTTCATAAGTTACAGAGATCAATCCAATGTAGGAGACGACTCCTT
  VIT_01s0127g00870_SV1    (923)  GATACTTTCATAAGTTACAGAGATCAATCCAATGTAGGAGACGACTCCTT
              Consensus    (951)  GATACTTTCATAAGTTACAGAGATCAATCCAATGTAGGAGACGACTCCTT
                                  1001                                             1050
VIT_01s0127g00870_CRIBI   (1001)  CAAATCCTACGCCAAGAATTCCAATTCCGCAGAGGTGGATTTCGTCAACT
  VIT_01s0127g00870_SV1    (973)  CAAATCCTACGCCAAGAATTCCAATTCCGCAGAGGTGGATTTCGTCAACT
              Consensus   (1001)  CAAATCCTACGCCAAGAATTCCAATTCCGCAGAGGTGGATTTCGTCAACT
                                  1051                                             1100
VIT_01s0127g00870_CRIBI   (1051)  ATGGGAAATCGTTCAACGAAGGTACAGACACTTTCGCTGGGTACGGCAAG
  VIT_01s0127g00870_SV1   (1023)  ATGGGAAATCGTTCAACGAAGGTACAGACACTTTCGCTGGGTACGGCAAG
              Consensus   (1051)  ATGGGAAATCGTTCAACGAAGGTACAGACACTTTCGCTGGGTACGGCAAG
                                  1101                                             1150
VIT_01s0127g00870_CRIBI   (1101)  GGAGCGACGAACCACAAAATTAATTTCAAGATCTACGGCGTCAACAACAC
  VIT_01s0127g00870_SV1   (1073)  GGAGCGACGAACCACAAAATTAATTTCAAGATCTACGGCGTCAACAACAC
              Consensus   (1101)  GGAGCGACGAACCACAAAATTAATTTCAAGATCTACGGCGTCAACAACAC
                                  1151                                             1200
VIT_01s0127g00870_CRIBI   (1151)  ATTCACAGACTACGCTAAAAAGGGCATCTCCTTTTCAAGATACACCAACA
  VIT_01s0127g00870_SV1   (1123)  ATTCACAGACTACGCTAAAAAGGGCATCTCCTTTTCAAGATACACTAACA
              Consensus   (1151)  ATTCACAGACTACGCTAAAAAGGGCATCTCCTTTTCAAGATACAC AACA
                                  1201                                             1250
VIT_01s0127g00870_CRIBI   (1201)  AAAGCTCTGAAACAATGACTTCCATGGCGGTCAGTGGCAGTTCAGTAAAT
  VIT_01s0127g00870_SV1   (1173)  AAAGCTCTGAAACAATGACTTCCATGGCGGTCAGTGGCAGTTCAGTAAAT
              Consensus   (1201)  AAAGCTCTGAAACAATGACTTCCATGGCGGTCAGTGGCAGTTCAGTAAAT
                                  1251                                             1300
VIT_01s0127g00870_CRIBI   (1251)  AGGTGGGTTGAGCCGGGTAAATTCTTCCGCGAGTCGATGCTGAAGAAAGG
  VIT_01s0127g00870_SV1   (1223)  AGGTGGGTTGAGCCGGGTAAATTCTTCCGCGAGTCGATGCTGAAGAAAGG
              Consensus   (1251)  AGGTGGGTTGAGCCGGGTAAATTCTTCCGCGAGTCGATGCTGAAGAAAGG
                                  1301                                             1350
VIT_01s0127g00870_CRIBI   (1301)  GACTGTGATGCCAATGCCGGACATTAGGGATAAAATGCCCAAAAGGTCGT
  VIT_01s0127g00870_SV1   (1273)  GACTGTGATGCCAATGCCGGACATTAGGGATAAAATGCCCAAAAGGTCGT
              Consensus   (1301)  GACTGTGATGCCAATGCCGGACATTAGGGATAAAATGCCCAAAAGGTCGT
                                  1351                                             1400
VIT_01s0127g00870_CRIBI   (1351)  TTTTGCCCCGCACTATTTCCTCCAAATTTCCGTTTTCAACCTCCAAACTT
  VIT_01s0127g00870_SV1   (1323)  TTTTGCCCCGCACTATTTCCTCCAAATTACCGTTTTCAACCTCCAAACTT
              Consensus   (1351)  TTTTGCCCCGCACTATTTCCTCCAAATT CCGTTTTCAACCTCCAAACTT
                                  1401                                             1450
VIT_01s0127g00870_CRIBI   (1401)  GAGGAGATGAAGAAAATATTCCATGCCGCGGATAACTCAAGCATGGAGCA
  VIT_01s0127g00870_SV1   (1373)  GAGGAGATGAAGAAAATATTCCATGCCGCGGATAACTCAAGCATGGAGCA
              Consensus   (1401)  GAGGAGATGAAGAAAATATTCCATGCCGCGGATAACTCAAGCATGGAGCA
                                  1451                                             1500
VIT_01s0127g00870_CRIBI   (1451)  CATGTTCACAGAGGCATTAGATGACTGCGAGAGAGCACCCAGCAAAGGCG
  VIT_01s0127g00870_SV1   (1423)  CATGTTCACAGAGGCATTAGATGACTGCGAGAGAGCACCCAGCAAAGGCG
              Consensus   (1451)  CATGTTCACAGAGGCATTAGATGACTGCGAGAGAGCACCCAGCAAAGGCG
                                  1501                                             1550
VIT_01s0127g00870_CRIBI   (1501)  AGACCAAGCGCTGCGTCCCCTCCATCGAGGACATGATCGACTTCGCCACC
  VIT_01s0127g00870_SV1   (1473)  AGACCAAGCGCTGCGTCCCCTCCATCGAGGACATGATCGACTTCGCCACC
              Consensus   (1501)  AGACCAAGCGCTGCGTCCCCTCCATCGAGGACATGATCGACTTCGCCACC
                                  1551                                             1600
VIT_01s0127g00870_CRIBI   (1551)  ACCGTCCTCGGCCGCAACGTTGTGGTTCGCACCACCCAATCCGTCGAAGG
  VIT_01s0127g00870_SV1   (1523)  ACCGTCCTCGGCCGCAACGTTGTGGTTCGCACCACCCAATCCGTCGAAGG
              Consensus   (1551)  ACCGTCCTCGGCCGCAACGTTGTGGTTCGCACCACCCAATCCGTCGAAGG
```

```
                                1601                                              1650
VIT_01s0127g00870_CRIBI  (1601) GTCAAAACAAAACTTAATGATCGGGTCGGTCAAAGGGATCAACGGTGGAC
  VIT_01s0127g00870_SV1  (1573) GTCAAAACAAAACGTAATGATCGGGTCGGTCAAAGGGATCAACGGTGGAC
              Consensus  (1601) GTCAAAACAAAAC TAATGATCGGGTCGGTCAAAGGGATCAACGGTGGAC
                                1651                                              1700
VIT_01s0127g00870_CRIBI  (1651) AAGTCACCAAATCGGTGTCCTGCCACCAGAGCCTGTTCCCTTATCTACTC
  VIT_01s0127g00870_SV1  (1623) AAGTCACCAAATCGGTGTCCTGCCACCAGAGCCTGTTCCCTTATCTACTC
              Consensus  (1651) AAGTCACCAAATCGGTGTCCTGCCACCAGAGCCTGTTCCCTTATCTACTC
                                1701                                              1750
VIT_01s0127g00870_CRIBI  (1701) TATTACTGCCACTCCGTTCCCAAGGTTCGGGTCTATGAAGCGGACCTCCT
  VIT_01s0127g00870_SV1  (1673) TATTACTGCCACTCCGTTCCCAAGGTTCGGGTCTATGAAGCGGACCTCCT
              Consensus  (1701) TATTACTGCCACTCCGTTCCCAAGGTTCGGGTCTATGAAGCGGACCTCCT
                                1751                                              1800
VIT_01s0127g00870_CRIBI  (1751) GGACCCGAAAACCAAAGCCAACGTTAACCATGGTGTTGCCATCTGTCACT
  VIT_01s0127g00870_SV1  (1723) GGACCCGAAAACCAAAGCCAACGTTAACCATGGTGTTGCCATCTGTCACT
              Consensus  (1751) GGACCCGAAAACCAAAGCCAACGTTAACCATGGTGTTGCCATCTGTCACT
                                1801                                              1850
VIT_01s0127g00870_CRIBI  (1801) TGGACACCTCCGATTGGAGCGCGGGTCATGGTGCATTCGTGGCATTGGGT
  VIT_01s0127g00870_SV1  (1773) TGGACACCTCCGATTGGAGCGCGGGTCATGGTGCATTCGTGGCATTGGGT
              Consensus  (1801) TGGACACCTCCGATTGGAGCGCGGGTCATGGTGCATTCGTGGCATTGGGT
                                1851                                              1900
VIT_01s0127g00870_CRIBI  (1851) TCGGGCCCTGGCCGGATTGAAGTTTGTCACTGGATTTTCGAGAATGATAT
  VIT_01s0127g00870_SV1  (1823) TCGGGCCCTGGCCGGATTGAAGTTTGTCACTGGATCTTCGAGAATGATAT
              Consensus  (1851) TCGGGCCCTGGCCGGATTGAAGTTTGTCACTGGAT TTCGAGAATGATAT
                                1901                                              1950
VIT_01s0127g00870_CRIBI  (1901) GACCTGGACCATTGCAGACTGAGCCATGACCCACCTTCCCGTCTGGGTTC
  VIT_01s0127g00870_SV1  (1873) GACCTGGACCATTGTAGACTGATTCATGACCCACCTTGTCAGCTCAGTTC
              Consensus  (1901) GACCTGGACCATTG AGACTGA  CATGACCCACCTT  C  CT  GTTC
                                1951                                              2000
VIT_01s0127g00870_CRIBI  (1951) GAACCACTTGGTCAACAAAGCTGGCTCGACCCCAACTTCCGCTTATGTAT
  VIT_01s0127g00870_SV1  (1923) GAACCACTTGGTCAACCAAGCTGGCGCGACCCCGACATCTGCTTGTTCGT
              Consensus  (1951) GAACCACTTGGTCAAC AAGCTGGC CGACCCC AC TC GCTT T   T
                                2001                                              2050
VIT_01s0127g00870_CRIBI  (2001) TTCCTTCATAAGATGTCATGGTGAAATATTT-AAGGCATGTGTTTCACCA
  VIT_01s0127g00870_SV1  (1973) TTCCTTCATAACGAGTCATGGTGAAATATTTCAAGGCATTTGCTTCATCT
              Consensus  (2001) TTCCTTCATAA   GTCATGGTGAAATATTT AAGGCAT TG TTCA C
                                2051                                              2100
VIT_01s0127g00870_CRIBI  (2050) GGCTAAGGATGGTAATTTATTGTATGTTATCATTATCCCAGTGTTGAGAG
  VIT_01s0127g00870_SV1  (2023) ATGTAAGGATGGTAATTTATCTCAG-------TTGTGCGAGAGCTAAG-G
              Consensus  (2051)    TAAGGATGGTAATTTAT   A        TT T C AG G T AG G
                                2101                                              2150
VIT_01s0127g00870_CRIBI  (2100) CGTGTCTTTGATGGCTTCCTCATTGGTAGGAGGGTGAAATGCAATGAATG
  VIT_01s0127g00870_SV1  (2065) AAGGATTAAAATG--TTGAAAATTATAAAAT-------------------
              Consensus  (2101)    G  T   ATG  TT    ATT   A  A
                                2151          2168
VIT_01s0127g00870_CRIBI  (2150) AATGGCTTGTTCTTCTTT
  VIT_01s0127g00870_SV1  (2095) ------------------
              Consensus  (2151)
```

VvBURP02
Protein alignment

```
                                        1                                                50
        GSVIVP00002688001      (1)   --------------MQVALGGGSEVAGESRDGANPFTPKAALVRYWNKQI
VIT_01s0127g00850_CRIBI      (1)   MSHLFLIFLYSALAFNVALGGGSEVAGESRDGANPFTPKAALVRYWNKQI
  VIT_01s0127g00850_SV1      (1)   MSHLFLIFLYSALAFNVALGGGSEVAGESRDGANPFTPKAALVRYWNKQI
                                        51                                              100
        GSVIVP00002688001     (37)   GSDLQKSTFLLSRASPLSAVESATFTKLAAQNALSNYLPAFCKSANLFCF
VIT_01s0127g00850_CRIBI     (51)   GSDLQKSTFLLSRASPLSAVESATFTKLAAQNALSNYLPAFCKSANLFCF
  VIT_01s0127g00850_SV1     (51)   GSDLQKSTFLLSRASPLSAVESATFTKLAAQNALSNYLPAFCKSANLFCF
                                        101                                             150
        GSVIVP00002688001     (87)   PDLGQSLEKHGPNSNFAVYSNKNFTNYGTGGHGGTDSFKNYTDYFPVDSF
VIT_01s0127g00850_CRIBI    (101)   PDLGQSLEKHGPNSNFAVYSNKNFTNYGTGGHGGTDSFKNYTDYFPVDSF
  VIT_01s0127g00850_SV1    (101)   PDLGQSLEKHGPNSNFAVYSNKNFTNYGTGGHGGTDSFKNYTDYFPVDSF
                                        151                                             200
        GSVIVP00002688001    (137)   RRYSRDSAGHNDNFDNYGPEGNTMDLSFNGYGSGATRGVGEFKKYSEESN
VIT_01s0127g00850_CRIBI    (151)   RRYSRDSAGHNDNFDNYGPEGNTMDLSFNGYGSGATRGVGEFKKYSEESN
  VIT_01s0127g00850_SV1    (151)   RRYSRDSAGHNDNFDNYGPEGNTMDLSFNGYGSGATRGVGEFKKYSEESN
                                        201                                             250
        GSVIVP00002688001    (187)   VPNVRFNSYSSDGNTRKQSFRSYSTESNAGDQYFTSYGKNSQGSPNEFTS
VIT_01s0127g00850_CRIBI    (201)   VPNVRFNSYSSDGNTRKQSFRSYSTESNAGDQYFTSYGKNSQGSPNEFTS
  VIT_01s0127g00850_SV1    (201)   VPNVRFNSYSSDGNTRKQSFRSYSTESNAGDQYFTSYGKNSQGSPNEFTS
                                        251                                             300
        GSVIVP00002688001    (237)   YGENTNVIGSTFTGYGRTANAANDKFTSYGFGGNVPVNNFKSYGDGGNSG
VIT_01s0127g00850_CRIBI    (251)   YGENTNVIGSTFTGYGRTANAANDKFTSYGFGGNVPVNNFKSYGDGGNSG
  VIT_01s0127g00850_SV1    (251)   YGENTNVIGSTFTGYGRTANAANDKFTSYGFGGNVPVNNFKSYGDGGNSG
                                        301                                             350
        GSVIVP00002688001    (287)   IDTFKSYRNQSNVGDDSFRSYAKNSHSAEVNFANYGQSFNEGTDTFTGYG
VIT_01s0127g00850_CRIBI    (301)   IDTFKSYRNQSNVGDDSFRSYAKNSHSAEVNFANYGQSFNEGTDTFTGYG
  VIT_01s0127g00850_SV1    (301)   IDTFKSYRNQSNVGDDSFRSYAKNSHSAEVNFANYGQSFNEGTDTFTGYG
                                        351                                             400
        GSVIVP00002688001    (337)   KGATGHKIGFKFYGVNNTFTDYAKKGISFSRYTNKSSETMISTAANGSSV
VIT_01s0127g00850_CRIBI    (351)   KGATGHKIGFKFYGVNNTFTDYAKKGISFSRYTNKSSETMISTAANGSSV
  VIT_01s0127g00850_SV1    (351)   KGATGHKIGFKFYGVNNTFTDYAK--------------------------
                                        401                                             450
        GSVIVP00002688001    (387)   NRWIEPGKFFRESMLKKGTVMPMPDIRDKMPKRSFLPRSISSKLPFSTSK
VIT_01s0127g00850_CRIBI    (401)   NRWIEPGKFFRESMLKKGTVMPMPDIRDKMPKRSFLPRSISSKLPFSTSK
  VIT_01s0127g00850_SV1    (375)   --------------------------------------------------
                                        451                                             500
        GSVIVP00002688001    (437)   LEEMKKIFHAADNSSMEHMFTEALDDCERAPSKGETRRCVPSIEDMIDFA
VIT_01s0127g00850_CRIBI    (451)   LEEMKKIFHAADNSSMEHMFTEALDDCERAPSKGETRRCVPSIEDMIDFA
  VIT_01s0127g00850_SV1    (375)   --------------------------------------------------
                                        501                                             550
        GSVIVP00002688001    (487)   TTVLGRNVVVRTTQSVEGSKQNVMIGSVKGINGGQVTKSVSCHQSLFPYL
VIT_01s0127g00850_CRIBI    (501)   TTVLGRNVVVRTTQSVEGSKQNVMIGSVKGINGGQVTKSVSCHQSLFPYL
  VIT_01s0127g00850_SV1    (375)   --------------------------------------------------
                                        551                                             600
        GSVIVP00002688001    (537)   LYYCHFVPKVRVYEADLLDPKTKANINHGVAICHLDTSDWSAGHGAFVAL
VIT_01s0127g00850_CRIBI    (551)   LYYCHFVPKVRVYEADLLDPKTKANINHGVAICHLDTSDWSAGHGAFVAL
  VIT_01s0127g00850_SV1    (375)   --------------------------------------------------
                                        601                   624
        GSVIVP00002688001    (587)   GSGPGRIEVCHWIFENDMTWTIVD
VIT_01s0127g00850_CRIBI    (601)   GSGPGRIEVCHWIFENDMTWTIVD
  VIT_01s0127g00850_SV1    (375)   ------------------------
```

VvBURP03
Protein alignment

```
                                   1                                                50
     GSVIVP00036411001     (1)   ----------------------------------------MDDFPEGVNV
     VIT_03s0063g02340     (1)   MNLRFSFWSIFLHFLAVMCFHGSGAREMPREELMVAPKFPMDDFPEGVNV
 VIT_03s0063g02340_SV1     (1)   MNLRFSFWSIFLHFLAVMCFHGSGAREVPREELMVAPEFPMDDFPEGVNV
 VIT_03s0063g02340_SV2     (1)   ---------------------------------MVAPEFPMDDFPEGVNV
             Consensus     (1)   MNLRFSFWSIFLHFLAVMCFHGSGAREMPREELMVAPEFPMDDFPEGVNV
                                 51                                               100
     GSVIVP00036411001    (11)   LQLHSMDGSEKKDGGQHAMEDRLHGHDKRGEETRKREDTEHVHGHSSSHM
     VIT_03s0063g02340    (51)   LQLHSMDGSEKKDGGQHAMEDRLHGHDKRGEETRKREDTEHVHGHSSSHM
 VIT_03s0063g02340_SV1    (51)   LQLHSMDGSEKKDGGQHAMEDRLHGHDKRGEETRKREDTEHVHGHSSSHM
 VIT_03s0063g02340_SV2    (18)   LQLHSMDGSEKKDGGQHAMEDRLHGHDKRGEETRKREDTEHVHGHSSSHM
             Consensus    (51)   LQLHSMDGSEKKDGGQHAMEDRLHGHDKRGEETRKREDTEHVHGHSSSHM
                                 101                                              150
     GSVIVP00036411001    (61)   DHLDPSVVVFFTMKDLKVGKTMPIYFAKTDPASSPRMLPKEEADSIPFSF
     VIT_03s0063g02340   (101)   DHLDPSVVVFFTMKDLKVGKTMPIYFAKTDPASSPRMLPKEEADSIPFSF
 VIT_03s0063g02340_SV1   (101)   DHLDPSVVVFFTMKDLKVGKTMPIYFAKTDPASSPRMLPKEEADSIPFSF
 VIT_03s0063g02340_SV2    (68)   DHLDPSVVVFFTMKDLKVGKTMPIYFAKTDPASSPRMLPKEEADSIPFSF
             Consensus   (101)   DHLDPSVVVFFTMKDLKVGKTMPIYFAKTDPASSPRMLPKEEADSIPFSF
                                 151                                              200
     GSVIVP00036411001   (111)   AQLPHLLEFFSFSQGSPQARAMENTLRECGLKPIRGETKFCATSLESLLD
     VIT_03s0063g02340   (151)   AQLPHLLEFFSFSQGSPQARAMENTLRECGLKPIRGETKFCATSLESLLD
 VIT_03s0063g02340_SV1   (151)   AQLPHLLEFFSFSQGSPQARAMENTLRECELKPIKGETKFCATSLESLLD
 VIT_03s0063g02340_SV2   (118)   AQLPHLLEFFSFSQGSPQARAMENTLRECELKPIKGETKFCATSLESLLD
             Consensus   (151)   AQLPHLLEFFSFSQGSPQARAMENTLRECGLKPIKGETKFCATSLESLLD
                                 201                                              250
     GSVIVP00036411001   (161)   FVHSIFGLESHFQVLTTSYLTKSSTLFQNYTFLEVPTEIPAPKMVACHTM
     VIT_03s0063g02340   (201)   FVHSIFGLESHFQVLTTSYLTKSSTLFQNYTFLEVPTEIPAPKMVACHTM
 VIT_03s0063g02340_SV1   (201)   FVHSIFGLESHFQVLTTSYLTKSSTLFQNYTFLEVPTEIPAPKMVACHTM
 VIT_03s0063g02340_SV2   (168)   FVHSIFGLESHFQVLTTSYLTKSSTLFQNYTFLEVPTEIPAPKMVACHTM
             Consensus   (201)   FVHSIFGLESHFQVLTTSYLTKSSTLFQNYTFLEVPTEIPAPKMVACHTM
                                 251                                              300
     GSVIVP00036411001   (211)   PYPYAIFYCHSQVSENKVFKVSLEGQNGDRVEAFAVCHLDTSAWSRDHVS
     VIT_03s0063g02340   (251)   PYPYAIFYCHSQVSENKVFKVSLEGQNGDRVEAFAVCHLDTSAWSRDHVS
 VIT_03s0063g02340_SV1   (251)   PYPYAIFYCHSQASENKVFKVSLEGQNGDRVEAFAVCHLDTSAWSRDHVS
 VIT_03s0063g02340_SV2   (218)   PYPYAIFYCHSQASENKVFKVSLEGQNGDRVEAFAVCHLDTSAWSRDHVS
             Consensus   (251)   PYPYAIFYCHSQVSENKVFKVSLEGQNGDRVEAFAVCHLDTSAWSRDHVS
                                 301                           331
     GSVIVP00036411001   (261)   FRVLGIEPGTSPVCHFFPASNLIWVPRPTLN
     VIT_03s0063g02340   (301)   FRVLGIEPGTSPVCHFFPASNLIWVPRPTLN
 VIT_03s0063g02340_SV1   (301)   FRVLGIEPGTSPVCHFFPASNLIWVPRPTLN
 VIT_03s0063g02340_SV2   (268)   FRVLGIEPGTSPVCHFFPASNLIWVPRPTLN
             Consensus   (301)   FRVLGIEPGTSPVCHFFPASNLIWVPRPTLN
```

VvBURP03
DNA alignment

```
                                   1                                                50
    GSVIVT00036411001      (1)  AATTTAAAAACCCCACTTTCTGTTTCCTTGAATTATCAAAAGCTTCCGTG
    VIT_03s0063g02340      (1)  AATTTCAAAACCCCACTTTCTGTTTCCTTGAATTATCAAAAGCTTCCGTG
VIT_03s0063g02340_SV1      (1)  --------------------------------------------------
VIT_03s0063g02340_SV2      (1)  --------------------------------------------------
            Consensus      (1)  AATTT AAAACCCCACTTTCTGTTTCCTTGAATTATCAAAAGCTTCCGTG
                                51                                               100
    GSVIVT00036411001     (51)  GATTTAATTTCATCTCGTAGAATGAGTGGATTGTTTAAGTTTTGCCTAAT
    VIT_03s0063g02340     (51)  GATTTAATTTCATATTGTAGAATCTATGGATTGTTTAAGTTTTGCCTAAT
VIT_03s0063g02340_SV1      (1)  --------------------------------------------------
VIT_03s0063g02340_SV2      (1)  --------------------------------------------------
            Consensus     (51)  GATTTAATTTCAT T GTAGAAT   TGGATTGTTTAAGTTTTGCCTAAT
                                101                                              150
    GSVIVT00036411001    (101)  TGGCTTTGGCCTAGGTGATCAAAGGCATCATTTCTTTGAGGAAGTCGTCT
    VIT_03s0063g02340    (101)  TGGCTTTGGCCTAGGTGATCAAAGGCATCATTTCTTTGAGGAAGCCGTCT
VIT_03s0063g02340_SV1      (1)  ----------------TCAGGTTCATTTTTCTTCTTTTCTGGGAAATCCT
VIT_03s0063g02340_SV2      (1)  ----------------TCAGGTTCATTTTTCTTCTTTTCTGGGAAATCCT
            Consensus    (101)  TGGCTTTGGCCTAGGTTCTGGTTGGTTTTTTTTCTTTTCTGGGGACTTCT
                                151                                              200
    GSVIVT00036411001    (151)  GACTTCAACTCCTTACTACTCTCAGATGTAATATGAATTTGCCCTTGTGG
    VIT_03s0063g02340    (151)  GGCTTCAACTCCTTACTACTCTCAGAT-----------------------
VIT_03s0063g02340_SV1     (35)  TTCTTCAACTCCTTACTACTCTCAGAT-----------------------
VIT_03s0063g02340_SV2     (35)  TTCTTCAACTCCTTACTACTCTCAGAT-----------------------
            Consensus    (151)  TTCTTCAACTCCTTACTACTCTCAGAT
                                201                                              250
    GSVIVT00036411001    (201)  TATTCGATTGAATGCTCCTTTGTTTGCTGACTAGTTCTGTTGGTCTCTTT
    VIT_03s0063g02340    (178)  --------------------------------------------------
VIT_03s0063g02340_SV1     (62)  --------------------------------------------------
VIT_03s0063g02340_SV2     (62)  --------------------------------------------------
            Consensus    (201)
                                251                                              300
    GSVIVT00036411001    (251)  GTTGTCTTACCAGAAGGAGAAGATAAAGAAGAAGAAGAAAGGATTTCCCA
    VIT_03s0063g02340    (178)  -------------AAGGAGAAGATAAAGAAGAAGAAGAAAGGATTTCCCA
VIT_03s0063g02340_SV1     (62)  -------------AAGGAGAAGATAAAGAAGAAGAAGAAAGGATTTCCCA
VIT_03s0063g02340_SV2     (62)  -------------AAGGAGAAGATAAAGAAGAAGAAGAAAGGATTTCCCA
            Consensus    (251)               AAGGAGAAGATAAAGAAGAAGAAGAAAGGATTTCCCA
                                301                                              350
    GSVIVT00036411001    (301)  GAAAAGAAGAAAAATGAACCTGAGATTTTCCTTTTGGAGCATCTTCCTTC
    VIT_03s0063g02340    (215)  GAAAAGAAGAAAAATGAACCTGAGATTTTCCTTTTGGAGCATCTTCCTTC
VIT_03s0063g02340_SV1     (99)  GAAAAGAAGAAAAATGAACCTGAGATTTTCCTTTTGGAGCATCTTCCTTC
VIT_03s0063g02340_SV2     (99)  GAAAAGAAGAAAAATGAACCTGAGATTTTCCTTTTGGAGCATCTTCCTTC
            Consensus    (301)  GAAAAGAAGAAAAATGAACCTGAGATTTTCCTTTTGGAGCATCTTCCTTC
                                351                                              400
    GSVIVT00036411001    (351)  ACTTTCTGGCTGTGATGGTAACCTCTCCACTCTTCAATTTATTCATATAG
    VIT_03s0063g02340    (265)  ACTTTCTGGCTGTGATG---------------------------------
VIT_03s0063g02340_SV1    (149)  ACTTTCTGGCTGTGATG---------------------------------
VIT_03s0063g02340_SV2    (149)  ACTTTCTGGCTGTGATGGTAAACTCTCCACTCTTCAATTTATTCATACAG
            Consensus    (351)  ACTTTCTGGCTGTGATGGTAA CTCTCCACTCTTCAATTTATTCATA AG
                                401                                              450
    GSVIVT00036411001    (401)  CATGCATACATCTTTGCAGTCTCTTGGAACTTCTATGCCCCTGCAGTTCA
    VIT_03s0063g02340    (282)  --------------------------------------------------
VIT_03s0063g02340_SV1    (166)  --------------------------------------------------
VIT_03s0063g02340_SV2    (199)  CATGCATACATCTTTGCAGTCTCTTGGAACTTCTATGCCCCTGCAGTTCA
            Consensus    (401)  CATGCATACATCTTTGCAGTCTCTTGGAACTTCTATGCCCCTGCAGTTCA
```

```
                                    451                                               500
     GSVIVT00036411001     (451) AGACGATGATGATGATTAAATACTGGTATAATTGTTATTATTTGATTGAC
     VIT_03s0063g02340     (282) --------------------------------------------------
 VIT_03s0063g02340_SV1     (166) --------------------------------------------------
 VIT_03s0063g02340_SV2     (249) AGACGATGATGATGATTAAATACTGGTATAATTGTTATTATTTGATTGAC
             Consensus     (451) AGACGATGATGATGATTAAATACTGGTATAATTGTTATTATTTGATTGAC
                                    501                                               550
     GSVIVT00036411001     (501) ATAATCTGTGGAAGCTGGAAATTTTGATGTTCATGGTAAAGTACAAAGAT
     VIT_03s0063g02340     (282) --------------------------------------------------
 VIT_03s0063g02340_SV1     (166) --------------------------------------------------
 VIT_03s0063g02340_SV2     (299) ATAATCTGTGGAAGCTGGAAATTTTGATGTTCATGGTAAAGTACAAAGAT
             Consensus     (501) ATAATCTGTGGAAGCTGGAAATTTTGATGTTCATGGTAAAGTACAAAGAT
                                    551                                               600
     GSVIVT00036411001     (551) TTTAGCTGGTAATATCATTTTCCATTTGAATGGAAATATAAGTAGTTCTA
     VIT_03s0063g02340     (282) --------------------------------------------------
 VIT_03s0063g02340_SV1     (166) --------------------------------------------------
 VIT_03s0063g02340_SV2     (349) TTTAGCTGGTAATATCATTTTCCATTTGAATGGAAATATAAGTAGTTCTA
             Consensus     (551) TTTAGCTGGTAATATCATTTTCCATTTGAATGGAAATATAAGTAGTTCTA
                                    601                                               650
     GSVIVT00036411001     (601) AATTTCATCATAATGGATGACGCATTTTTAGTTTTCTTTTCAAGGGTTTT
     VIT_03s0063g02340     (282) --------------------------------------------------
 VIT_03s0063g02340_SV1     (166) --------------------------------------------------
 VIT_03s0063g02340_SV2     (399) AATTTCATCATAATGGATGAC-CATTTTTAGTTTTCTTTTCAAGGGTTTT
             Consensus     (601) AATTTCATCATAATGGATGAC CATTTTTAGTTTTCTTTTCAAGGGTTTT
                                    651                                               700
     GSVIVT00036411001     (651) CGCATGGTTTTAGACAAATTTTCTTAGAAACCTTGTTTACTAAGTCATGT
     VIT_03s0063g02340     (282) --------------------------------------------------
 VIT_03s0063g02340_SV1     (166) --------------------------------------------------
 VIT_03s0063g02340_SV2     (448) CGCATGGTTTTAGACAAATTTTCTTAGAAACCTTGTTTACTAAGTCATGT
             Consensus     (651) CGCATGGTTTTAGACAAATTTTCTTAGAAACCTTGTTTACTAAGTCATGT
                                    701                                               750
     GSVIVT00036411001     (701) TACAGATCTCCATATTGTAAGCTGCAGACATTTCTCCCCATTTTTGTAGT
     VIT_03s0063g02340     (282) -------------------------------------------------T
 VIT_03s0063g02340_SV1     (166) -------------------------------------------------T
 VIT_03s0063g02340_SV2     (498) TACAGATCTCCATATTCTAAG----------------------------T
             Consensus     (701) TACAGATCTCCATATT TAAG                            T
                                    751                                               800
     GSVIVT00036411001     (751) GTTTCCATGGAAGTGGAGCCAGGGAGATGCCTAGAGAGGAGTTGATGGTG
     VIT_03s0063g02340     (283) GTTTCCATGGAAGTGGAGCCAGGGAGATGCCTAGAGAGGAGTTGATGGTG
 VIT_03s0063g02340_SV1     (167) GTTTCCATGGAAGTGGAGCCAGGGAGGTGCCTAGAGAGGAGTTGATGGTG
 VIT_03s0063g02340_SV2     (520) GTTTCCATGGAAGTGGAGCCAGGGAGGTGCCTAGAGAGGAGTTGATGGTG
             Consensus     (751) GTTTCCATGGAAGTGGAGCCAGGGAGGTGCCTAGAGAGGAGTTGATGGTG
                                    801                                               850
     GSVIVT00036411001     (801) GCTCCAAAATTTCCCATGGATGATTTCCCAGAAGGGGTTAATGTATTGCA
     VIT_03s0063g02340     (333) GCTCCAAAATTTCCCATGGATGATTTCCCAGAAGGGGTTAATGTATTGCA
 VIT_03s0063g02340_SV1     (217) GCTCCAGAATTTCCCATGGATGATTTCCCAGAAGGGGTTAATGTATTGCA
 VIT_03s0063g02340_SV2     (570) GCTCCAGAATTTCCCATGGATGATTTCCCAGAAGGGGTTAATGTATTGCA
             Consensus     (801) GCTCCAGAATTTCCCATGGATGATTTCCCAGAAGGGGTTAATGTATTGCA
                                    851                                               900
     GSVIVT00036411001     (851) ACTCCATAGCATGGATGGAAGTGAGAAAAAGGATGGTGGGCAACATGCCA
     VIT_03s0063g02340     (383) ACTCCATAGCATGGATGGAAGTGAGAAAAAGGATGGTGGGCAACATGCCA
 VIT_03s0063g02340_SV1     (267) ACTCCATAGCATGGATGGAAGTGAGAAAAAGGATGGTGGGCAACATGCCA
 VIT_03s0063g02340_SV2     (620) ACTCCATAGCATGGATGGAAGTGAGAAAAAGGATGGTGGGCAACATGCCA
             Consensus     (851) ACTCCATAGCATGGATGGAAGTGAGAAAAAGGATGGTGGGCAACATGCCA
                                    901                                               950
     GSVIVT00036411001     (901) TGGAGGACAGATTGCATGGTCATGATAAGCGAGGCGAAGAAACCCGAAAA
     VIT_03s0063g02340     (433) TGGAGGACAGATTGCATGGTCATGATAAGCGAGGCGAAGAAACCCGAAAA
 VIT_03s0063g02340_SV1     (317) TGGAAGACAGATTGCATGGTCATGATAAGCGAGGCGAAGAAACCCGAAAA
 VIT_03s0063g02340_SV2     (670) TGGAAGACAGATTGCATGGTCATGATAAGCGAGGCGAAGAAACCCGAAAA
             Consensus     (901) TGGAGGACAGATTGCATGGTCATGATAAGCGAGGCGAAGAAACCCGAAAA
```

```
                                   951                                           1000
     GSVIVT00036411001    (951)  AGGGAAGACACAGAGCATGTTCATGGTCATTCATCATCCCACATGGATCA
      VIT_03s0063g02340    (483)  AGGGAAGACACAGAGCATGTTCATGGTCATTCATCATCCCACATGGATCA
  VIT_03s0063g02340_SV1    (367)  AGGGAAGACACAGAGCATGTTCATGGTCATTCATCATCCCACATGGATCA
  VIT_03s0063g02340_SV2    (720)  AGGGAAGACACAGAGCATGTTCATGGTCATTCATCATCCCACATGGATCA
              Consensus    (951)  AGGGAAGACACAGAGCATGTTCATGGTCATTCATCATCCCACATGGATCA
                                   1001                                          1050
     GSVIVT00036411001   (1001)  CTTGGACCCTTCAGTTGTTGTCTTCTTCACTATGAAGGATCTGAAGGTTG
      VIT_03s0063g02340    (533)  CTTGGACCCTTCAGTTGTTGTCTTCTTCACTATGAAGGATCTGAAGGTTG
  VIT_03s0063g02340_SV1    (417)  CTTGGACCCTTCAGTTGTTGTCTTCTTCACTATGAAGGATCTGAAGGTTG
  VIT_03s0063g02340_SV2    (770)  CTTGGACCCTTCAGTTGTTGTCTTCTTCACTATGAAGGATCTGAAGGTTG
              Consensus   (1001)  CTTGGACCCTTCAGTTGTTGTCTTCTTCACTATGAAGGATCTGAAGGTTG
                                   1051                                          1100
     GSVIVT00036411001   (1051)  GGAAAACAATGCCCATCTACTTTGCCAAAACGGACCCTGCTTCTTCTCCT
      VIT_03s0063g02340    (583)  GGAAAACAATGCCCATCTACTTTGCCAAAACGGACCCTGCTTCTTCTCCT
  VIT_03s0063g02340_SV1    (467)  GGAAAACAATGCCCATCTACTTTGCCAAAACGGACCCTGCTTCTTCTCCT
  VIT_03s0063g02340_SV2    (820)  GGAAAACAATGCCCATCTACTTTGCCAAAACGGACCCTGCTTCTTCTCCT
              Consensus   (1051)  GGAAAACAATGCCCATCTACTTTGCCAAAACGGACCCTGCTTCTTCTCCT
                                   1101                                          1150
     GSVIVT00036411001   (1101)  CGCATGTTACCTAAAGAAGAAGCTGATTCCATTCCTTTCTCATTTGCCCA
      VIT_03s0063g02340    (633)  CGCATGTTACCTAAAGAAGAAGCTGATTCCATTCCTTTCTCATTTGCCCA
  VIT_03s0063g02340_SV1    (517)  CGCATGTTACCTAAAGAAGAAGCTGATTCCATTCCTTTCTCATTTGCCCA
  VIT_03s0063g02340_SV2    (870)  CGCATGTTACCTAAAGAAGAAGCTGATTCCATTCCTTTCTCATTTGCCCA
              Consensus   (1101)  CGCATGTTACCTAAAGAAGAAGCTGATTCCATTCCTTTCTCATTTGCCCA
                                   1151                                          1200
     GSVIVT00036411001   (1151)  ACTCCCACACCTCCTTGAATTCTTCTCCTTTTCTCAAGGCTCCCCCCAAG
      VIT_03s0063g02340    (683)  ACTCCCACACCTCCTTGAATTCTTCTCCTTTTCTCAAGGCTCCCCCCAAG
  VIT_03s0063g02340_SV1    (567)  ACTCCCACACCTCCTTGAATTCTTCTCCTTTTCTCAAGGCTCCCCCCAAG
  VIT_03s0063g02340_SV2    (920)  ACTCCCACACCTCCTTGAATTCTTCTCCTTTTCTCAAGGCTCCCCCCAAG
              Consensus   (1151)  ACTCCCACACCTCCTTGAATTCTTCTCCTTTTCTCAAGGCTCCCCCCAAG
                                   1201                                          1250
     GSVIVT00036411001   (1201)  CCAGAGCCATGGAAAATACACTTAGAGAATGTGGACTTAAGCCCATCAGA
      VIT_03s0063g02340    (733)  CCAGAGCCATGGAAAATACACTTAGAGAATGTGGACTTAAGCCCATCAGA
  VIT_03s0063g02340_SV1    (617)  CCAGAGCCATGGAAAATACACTTAGAGAATGTGAACTTAAGCCCATCAAA
  VIT_03s0063g02340_SV2    (970)  CCAGAGCCATGGAAAATACACTTAGAGAATGTGAACTTAAGCCCATCAAA
              Consensus   (1201)  CCAGAGCCATGGAAAATACACTTAGAGAATGTGGACTTAAGCCCATCAGA
                                   1251                                          1300
     GSVIVT00036411001   (1251)  GGAGAGACCAAGTTCTGTGCTACTTCCCTAGAATCCTTGCTTGATTTTGT
      VIT_03s0063g02340    (783)  GGAGAGACCAAGTTCTGTGCTACTTCCCTAGAATCCTTGCTTGATTTTGT
  VIT_03s0063g02340_SV1    (667)  GGAGAGACCAAGTTCTGTGCTACTTCCCTAGAATCCTTGCTTGATTTTGT
  VIT_03s0063g02340_SV2   (1020)  GGAGAGACCAAGTTCTGTGCTACTTCCCTAGAATCCTTGCTTGATTTTGT
              Consensus   (1251)  GGAGAGACCAAGTTCTGTGCTACTTCCCTAGAATCCTTGCTTGATTTTGT
                                   1301                                          1350
     GSVIVT00036411001   (1301)  GCATAGCATCTTTGGGCTGGAGTCCCATTTCCAAGTCTTAACAACTTCCT
      VIT_03s0063g02340    (833)  GCATAGCATCTTTGGGCTGGAGTCCCATTTCCAAGTCTTAACAACTTCCT
  VIT_03s0063g02340_SV1    (717)  GCATAGCATCTTTGGGCTGGAGTCCCATTTCCAAGTCTTAACAACTTCCT
  VIT_03s0063g02340_SV2   (1070)  GCATAGCATCTTTGGGCTGGAGTCCCATTTCCAAGTCTTAACAACTTCCT
              Consensus   (1301)  GCATAGCATCTTTGGGCTGGAGTCCCATTTCCAAGTCTTAACAACTTCCT
                                   1351                                          1400
     GSVIVT00036411001   (1351)  ATCTCACAAAGTCAAGTACCCTTTTCCAAAACTACACCTTCTTGGAAGTG
      VIT_03s0063g02340    (883)  ATCTCACAAAGTCAAGTACCCTTTTCCAAAACTACACCTTCTTGGAAGTG
  VIT_03s0063g02340_SV1    (767)  ATCTCACAAAGTCAAGTACCCTTTTCCAAAACTACACCTTCTTGGAAGTG
  VIT_03s0063g02340_SV2   (1120)  ATCTCACAAAGTCAAGTACCCTTTTCCAAAACTACACCTTCTTGGAAGTG
              Consensus   (1351)  ATCTCACAAAGTCAAGTACCCTTTTCCAAAACTACACCTTCTTGGAAGTG
                                   1401                                          1450
     GSVIVT00036411001   (1401)  CCTACAGAGATACCAGCTCCCAAAATGGTAGCTTGTCATACTATGCCCTA
      VIT_03s0063g02340    (933)  CCTACAGAGATACCAGCTCCCAAAATGGTAGCTTGTCATACTATGCCCTA
  VIT_03s0063g02340_SV1    (817)  CCTACAGAGATACCAGCTCCCAAAATGGTAGCTTGTCATACTATGCCCTA
  VIT_03s0063g02340_SV2   (1170)  CCTACAGAGATACCAGCTCCCAAAATGGTAGCTTGTCATACTATGCCCTA
              Consensus   (1401)  CCTACAGAGATACCAGCTCCCAAAATGGTAGCTTGTCATACTATGCCCTA
```

```
                                  1451                                             1500
     GSVIVT00036411001   (1451)  CCCTTATGCAATTTTCTACTGCCATTCCCAAGTGAGTGAGAACAAGGTGT
     VIT_03s0063g02340    (983)  CCCTTATGCAATTTTCTACTGCCATTCCCAAGTGAGTGAGAACAAGGTGT
 VIT_03s0063g02340_SV1    (867)  CCCTTATGCAATTTTCTACTGCCATTCCCAAGCGAGTGAGAACAAGGTGT
 VIT_03s0063g02340_SV2   (1220)  CCCTTATGCAATTTTCTACTGCCATTCCCAAGCGAGTGAGAACAAGGTGT
             Consensus   (1451)  CCCTTATGCAATTTTCTACTGCCATTCCCAAGTGAGTGAGAACAAGGTGT
                                  1501                                             1550
     GSVIVT00036411001   (1501)  TTAAGGTGTCACTAGAAGGCCAAAATGGAGATAGGGTGGAAGCCTTTGCT
     VIT_03s0063g02340   (1033)  TTAAGGTGTCACTAGAAGGCCAAAATGGAGATAGGGTGGAAGCCTTTGCT
 VIT_03s0063g02340_SV1    (917)  TTAAGGTGTCACTAGAAGGCCAAAATGGAGATAGGGTGGAAGCCTTTGCT
 VIT_03s0063g02340_SV2   (1270)  TTAAGGTGTCACTAGAAGGCCAAAATGGAGATAGGGTGGAAGCCTTTGCT
             Consensus   (1501)  TTAAGGTGTCACTAGAAGGCCAAAATGGAGATAGGGTGGAAGCCTTTGCT
                                  1551                                             1600
     GSVIVT00036411001   (1551)  GTTTGCCATTTGGATACCTCTGCCTGGAGCCGTGATCATGTGTCGTTCCG
     VIT_03s0063g02340   (1083)  GTTTGCCATTTGGATACCTCTGCCTGGAGCCGTGATCATGTGTCGTTCCG
 VIT_03s0063g02340_SV1    (967)  GTTTGCCATTTGGATACCTCTGCCTGGAGCCGTGATCATGTGTCATTCCG
 VIT_03s0063g02340_SV2   (1320)  GTTTGCCATTTGGATACCTCTGCCTGGAGCCGTGATCATGTGTCATTCCG
             Consensus   (1551)  GTTTGCCATTTGGATACCTCTGCCTGGAGCCGTGATCATGTGTCGTTCCG
                                  1601                                             1650
     GSVIVT00036411001   (1601)  TGTGCTCGGGATCGAGCCTGGGACATCCCCTGTGTGTCATTTCTTCCCTG
     VIT_03s0063g02340   (1133)  TGTGCTCGGGATCGAGCCTGGGACATCCCCTGTGTGTCATTTCTTCCCTG
 VIT_03s0063g02340_SV1   (1017)  TGTGCTCGGGATCGAGCCTGGGACATCCCCTGTGTGTCATTTCTTCCCTG
 VIT_03s0063g02340_SV2   (1370)  TGTGCTCGGGATCGAGCCTGGGACATCCCCTGTGTGTCATTTCTTCCCTG
             Consensus   (1601)  TGTGCTCGGGATCGAGCCTGGGACATCCCCTGTGTGTCATTTCTTCCCTG
                                  1651                                             1700
     GSVIVT00036411001   (1651)  CATCCAACCTCATATGGGTTCCGAGGCCTACTCTGAACTAGTCATGTAAC
     VIT_03s0063g02340   (1183)  CATCCAACCTCATATGGGTTCCGAGGCCTACTCTGAACTAGTCATGTAAC
 VIT_03s0063g02340_SV1   (1067)  CATCCAACCTCATATGGGTTCCGAGGCCTACTCTGAACTAGTCATGTAAC
 VIT_03s0063g02340_SV2   (1420)  CATCCAACCTCATATGGGTTCCGAGGCCTACTCTGAACTAGTCATGTAAC
             Consensus   (1651)  CATCCAACCTCATATGGGTTCCGAGGCCTACTCTGAACTAGTCATGTAAC
                                  1701                                             1750
     GSVIVT00036411001   (1701)  TGCAGTGTAATCACTGAAGCTATTACTATGATCTGGTATTCCACTTGGTG
     VIT_03s0063g02340   (1233)  TGCAGTGTAATCACTGAAGCTATTACTATGATCTGGTATTCCACTTGGTG
 VIT_03s0063g02340_SV1   (1117)  TGCAGTGTAATCACTGAAGCTATTACTATGATCTGGTATTCCACTTGGTG
 VIT_03s0063g02340_SV2   (1470)  TGCAGTGTAATCACTGAAGCTATTACTATGATCTGGTATTCCACTTGGTG
             Consensus   (1701)  TGCAGTGTAATCACTGAAGCTATTACTATGATCTGGTATTCCACTTGGTG
                                  1751                                             1800
     GSVIVT00036411001   (1751)  TTTGTTTTTCAACTGAAGGGCTCATAAATATAGAATAAATGAGTAGGTTT
     VIT_03s0063g02340   (1283)  TTTGTTTTTCAACTGAAGGGCTCATAAATATAGAATAAATGAGTAGGTTT
 VIT_03s0063g02340_SV1   (1167)  TTTGTTTTTCAACTGAAGGGCTCATAAATATAGAATAAATGAGTAGGTTT
 VIT_03s0063g02340_SV2   (1520)  TTTGTTTTTCAACTGAAGGGCTCATAAATATAGAATAAATGAGTAGGTTT
             Consensus   (1751)  TTTGTTTTTCAACTGAAGGGCTCATAAATATAGAATAAATGAGTAGGTTT
                                  1801                                             1850
     GSVIVT00036411001   (1801)  TGAATGTTGCTATATAAATAAGGGGTCTATTCAGAAGGTCAAGCAATCAA
     VIT_03s0063g02340   (1333)  TGAATGTTGCTATATAAATAAGGGGTCTATTCAGAAGGTCAAGCAATCAA
 VIT_03s0063g02340_SV1   (1217)  TGAATGTTGCTATATAAATAAGGGGTCTATTCAGAAGGTCAAGCAATCAA
 VIT_03s0063g02340_SV2   (1570)  TGAATGTTGCTATATAAATAAGGGGTCTATTCAGAAGGTCAAGCAATCAA
             Consensus   (1801)  TGAATGTTGCTATATAAATAAGGGGTCTATTCAGAAGGTCAAGCAATCAA
                                  1851                                             1900
     GSVIVT00036411001   (1851)  TAGTGACTCACCAGGTTTACTACTACAAGTTTTTATCAGTGGTAACATCT
     VIT_03s0063g02340   (1383)  TAGTGACTCACCAGGTTTACTACTACAAGTTTTTATCAGTGGTAACATCT
 VIT_03s0063g02340_SV1   (1267)  TAGTGACTCACCAGGTTTACTACTACAAGTTTTTATCAGTGGTAACATCT
 VIT_03s0063g02340_SV2   (1620)  TAGTGACTCACCAGGTTTACTACTACAAGTTTTTATCAGTGGTAACATCT
             Consensus   (1851)  TAGTGACTCACCAGGTTTACTACTACAAGTTTTTATCAGTGGTAACATCT
                                  1901                                             1950
     GSVIVT00036411001   (1901)  CATGCTGAATAGTTTT----------------------------------
     VIT_03s0063g02340   (1433)  CATGCTGAATAGTTTTGTGCAAG---------------------------
 VIT_03s0063g02340_SV1   (1317)  CATGCTGAATAGTTTTGTGCAAGAAATTTCTCTGCTTCTTGATTTTCAGT
 VIT_03s0063g02340_SV2   (1670)  CATGCTGAATAGTTTTGTGCAAGAAATTTCTCTGCTTCTTGATTTTCAGT
             Consensus   (1901)  CATGCTGAATAGTTTTGTGCAAGAAATTTCTCTGCTTCTTGATTTTCAGT
```

```
                               1951                           1982
     GSVIVT00036411001  (1917) --------------------------------
     VIT_03s0063g02340  (1456) --------------------------------
 VIT_03s0063g02340_SV1  (1367) TTCTTTGATGAATCATTTTATGTCAACAGGCG
 VIT_03s0063g02340_SV2  (1720) TTCTTTGATGAATCATTTTATGTCAACAGGCG
             Consensus  (1951) TTCTTTGATGAATCATTTTATGTCAACAGGCG
```

VvBURP04

Protein alignment

```
                              1                                                50
     GSVIVP00036412001    (1) -----------------------------------------MARKHIHTH
     VIT_03s0063g02320    (1) MGLGFSSSWSLSFCVLLVLMSAEASKGEYSLQEHEDGGEEAMARKHIHTH
 VIT_03s0063g02320_SV1    (1) MGLGFSSSWSLSFCVLLVLMSAEASKGEYSLQEHEDGGEEAMARKHIHTH
             Consensus    (1) MGLGFSSSWSLSFCVLLVLMSAEASKGEYSLQEHEDGGEEAMARKHIHTH
                              51                                              100
     GSVIVP00036412001   (10) MDMSMRIFFTITELKVGKRIPVYFSKRDPATSPHLLPREEVESIPFSSAQ
     VIT_03s0063g02320   (51) MDMSMRIFFTITELKVGKRIPVYFSKRDPATSPHLLPREEVESIPFSSAQ
 VIT_03s0063g02320_SV1   (51) MDMSMRIFFTITELKVGKRIPVYFSKRDPATSPHLLPREEVESIPFSSAQ
             Consensus   (51) MDMSMRIFFTITELKVGKRIPVYFSKRDPATSPHLLPREEVESIPFSSAQ
                              101                                             150
     GSVIVP00036412001   (60) LPYLLQFFGFSQGSPQAIAMENTLRHCETEPIEGETKSCVTSLESMLDFS
     VIT_03s0063g02320  (101) LPYLLQFFGFSQGSPQAIAMENTLRHCETEPIEGETKSCVTSLESMLDFS
 VIT_03s0063g02320_SV1  (101) LPYLLQFFGFSQGSPQAIAMENTLRHCETEPIEGETKSCVTSLESMLDFS
             Consensus  (101) LPYLLQFFGFSQGSPQAIAMENTLRHCETEPIEGETKSCVTSLESMLDFS
                              151                                             200
     GSVIVP00036412001  (110) QKIFGLKASFEVISTKLGEKTTSLLQNYTILKLPKPISAPKMVACHTLPY
     VIT_03s0063g02320  (151) QKIFGLKASFEVISTKLGEKTTSLLQNYTILKLPKPISAPKMVACHTLPY
 VIT_03s0063g02320_SV1  (151) RKIFGLKASFEVISTKLGEKTTSLLQNYTILKLPKPISAPKMVACHTLPY
             Consensus  (151) QKIFGLKASFEVISTKLGEKTTSLLQNYTILKLPKPISAPKMVACHTLPY
                              201                                             250
     GSVIVP00036412001  (160) PYAVFYCHFQEGENKVFEVSLGGENGDRVEAVAVCHMDTSQWNQDHVSFR
     VIT_03s0063g02320  (201) PYAVFYCHFQEGENKVFEVSLGGENGDRVEAVAVCHMDTSQWNQDHVSFR
 VIT_03s0063g02320_SV1  (201) PYAVFYCHFQEGENKVFEVSLGGENGDRVEAVAVCHMDTSQWNQDHVSFR
             Consensus  (201) PYAVFYCHFQEGENKVFEVSLGGENGDRVEAVAVCHMDTSQWNQDHVSFR
                              251                            281
     GSVIVP00036412001  (210) LLGVQPGASPVCHFFPADNLIWVPSPALIQD
     VIT_03s0063g02320  (251) LLGVQPGASPVCHFFPADNLIWVPSPALIQD
 VIT_03s0063g02320_SV1  (251) LLGVQPGASPVCHFFPADNLIWVPSPALIQD
             Consensus  (251) LLGVQPGASPVCHFFPADNLIWVPSPALIQD
```

VvBURP07

DNA alignment (SV2: 1 ORF; SV1 has two ORFs producing the same protein as SV1 but Split in 2)

```
                                    1                                                50
GSVIVT00032490001-32491001     (1)  --------------------------------------------ATACCA
          VIT_04s0008g03990    (1)  ---------------------------------------------TACCA
          VIT_04s0008g04000    (1)  --------------------------------------------------
      VIT_04s0008g03990_SV1    (1)  CTAACTTGCCTCTCTCTGCTGGTGGTTGTAAGCAATGCTTACAACCACCA
      VIT_04s0008g03990_SV2    (1)  CTAACTTGCCTCTCTCTGCTGGTGGTTGTAAGCAATGCTTACAACCACCA
                  Consensus    (1)                                              ACCA
                                    51                                              100
GSVIVT00032490001-32491001     (7)  ACGTACAGCTTAGCCTGCGGTTCATAGCTTCTATGGAGCTTCATCTTCTT
          VIT_04s0008g03990    (6)  ACGTACAGCTTAGCCTGCGGTTCACAGCTTCTATGGAGCTTCATCTTCTT
          VIT_04s0008g04000    (1)  --------------------------------------------------
      VIT_04s0008g03990_SV1   (51)  ACGTACAGCTTAGCCTGCGGTTCATAGCTTCTATGGAGCTTCATCTTCTT
      VIT_04s0008g03990_SV2   (51)  ACGTACAGCTTAGCCTGCGGTTCATAGCTTCTATGGAGCTTCATCTTCTT
                  Consensus   (51)  ACGTACAGCTTAGCCTGCGGTTCATAGCTTCTATGGAGCTTCATCTTCTT
                                    101                                             150
GSVIVT00032490001-32491001    (57)  CCCATTCTAACTTGCCTCTCTCTGCTGGTGGTTGTAAGCAATGCTTCTCT
          VIT_04s0008g03990   (56)  CCCATTCTAACCTGCCTCTCTCTGCTGGTGGTTGTAAACAATGCTTCTCT
          VIT_04s0008g04000    (1)  --------------------------------------------------
      VIT_04s0008g03990_SV1  (101)  CCCATTCTAACTTGCCTCTCTCTGCTGGTGGTTGTAAGCAATGCTTCTCT
      VIT_04s0008g03990_SV2  (101)  CCCATTCTAACTTGCCTCTCTCTGCTGGTGGTTGTAAGCAATGCTTCTCT
                  Consensus  (101)  CCCATTCTAACTTGCCTCTCTCTGCTGGTGGTTGTAAGCAATGCTTCTCT
                                    151                                             200
GSVIVT00032490001-32491001   (107)  ACCTTCTGAGGTCTACTGGAAGTTGGCTTTGCCGTATACTCCGGTGCCCA
          VIT_04s0008g03990  (106)  ACCTTCTGAGGTCTACTGGAAGTTGGCTTTGCCGTATACTCGGATGCCCA
          VIT_04s0008g04000    (1)  --------------------------------------------------
      VIT_04s0008g03990_SV1  (151)  ACCTTCTGAGGTCTACTGGAAGTTGGCTTTGCCGTATACTCCGATGCCCA
      VIT_04s0008g03990_SV2  (151)  ACCTTCTGAGGTCTACTGGAAGTTGGCTTTGCCGTATACTCCGATGCCCA
                  Consensus  (151)  ACCTTCTGAGGTCTACTGGAAGTTGGCTTTGCCGTATACTCCGATGCCCA
                                    201                                             250
GSVIVT00032490001-32491001   (157)  AAGCCATGCGAGATCTCTTGCAGCTGA-----------------------
          VIT_04s0008g03990  (156)  AAGTCGTGCGAGATCTCTTGCAGCTGA-----------------------
          VIT_04s0008g04000    (1)  --------------------------------------------------
      VIT_04s0008g03990_SV1  (201)  AAGCCATGCGAGATCTCTTGCAGCTGAGTTAGTTTCAATGAATCCCTAAC
      VIT_04s0008g03990_SV2  (201)  AAGCCATGCGAGATCTCTTGCAGCTGA-----------------------
                  Consensus  (201)  AAGCCATGCGAGATCTCTTGCAGCTGA
                                    251                                             300
GSVIVT00032490001-32491001   (184)  --------------------------------------------------
          VIT_04s0008g03990  (183)  --------------------------------------------------
          VIT_04s0008g04000    (1)  --------------------------------------------------
      VIT_04s0008g03990_SV1  (251)  TCATATGCAAATTTTTTTTTTCTTTTTTGAAATTTGGTGAAGAAAATAAT
      VIT_04s0008g03990_SV2  (228)  --------------------------------------------------
                  Consensus  (251)
                                    301                                             350
GSVIVT00032490001-32491001   (184)  --------------------------------------------------
          VIT_04s0008g03990  (183)  --------------------------------------------------
          VIT_04s0008g04000    (1)  --------------------------------------------------
      VIT_04s0008g03990_SV1  (301)  TTACTGTTAAATATTAGTTAGGCAACTGATTCTTAAGTAGCCACCTTGGC
      VIT_04s0008g03990_SV2  (228)  --------------------------------------------------
                  Consensus  (301)
                                    351                                             400
GSVIVT00032490001-32491001   (184)  --------------------------------------------------
          VIT_04s0008g03990  (183)  --------------------------------------------------
          VIT_04s0008g04000    (1)  --------------------------------------------------
      VIT_04s0008g03990_SV1  (351)  ATAATTGTAATAGCTAGTAAGGGGATATTATTCATATACGTTTCAATGAG
      VIT_04s0008g03990_SV2  (228)  --------------------------------------------------
                  Consensus  (351)
                                    401                                             450
GSVIVT00032490001-32491001   (184)  -------ACTCAATGGAAGGTGGAAGTTCAATCAATGTGAGCAAGGTTGT
          VIT_04s0008g03990  (183)  -------ACTCAATGGAAGGTGGAAGTTCAATCAATGTGAGCAAGGTTAT
          VIT_04s0008g04000    (1)  --------------------------------------------------
      VIT_04s0008g03990_SV1  (401)  TTTGCAGACTCAATGGAAGGTGGAAGTTCAATCAATGTGAGCAAGGTTGT
      VIT_04s0008g03990_SV2  (228)  -------ACTCAATGGAAGGTGGAAGTTCAATCAATGTGAGCAAGGTTGT
                  Consensus  (401)         ACTCAATGGAAGGTGGAAGTTCAATCAATGTGAGCAAGGTTGT
```

```
                                       451                                              500
GSVIVT00032490001-32491001     (227)  CCTAAATGCCATCCCAGGACCTGTCTATATCAATTGCAGAGTCCCACCGG
         VIT_04s0008g03990     (226)  ACTAAATGCCATCCCAGCGGATGTCTTTATCAAATACCAAAACCCATCGG
         VIT_04s0008g04000       (1)  --------------------------------------------------
     VIT_04s0008g03990_SV1     (451)  ACTAAATGCCATCCCAGGACCTGTCTATATCAATTGCAGAGTCCCACCGG
     VIT_04s0008g03990_SV2     (271)  ACTAAATGCCATCCCAGGACCTGTCTATATCAATTGCAGAGTCCCACCGG
                 Consensus     (451)  ACTAAATGCCATCCCAGGACCTGTCTATATCAATTGCAGAGTCCCACCGG
                                       501                                              550
GSVIVT00032490001-32491001     (277)  CTGCTGACACCCCCACTAAAGACCAACCTCAAGACACCTCCAGAAAAGGT
         VIT_04s0008g03990     (276)  CTGCTGACACCCCCACTGAAGACCAACCTC----------CAGAAAAGGT
         VIT_04s0008g04000       (1)  --------------------------------------------------
     VIT_04s0008g03990_SV1     (501)  CTGTTGACACCCCCACTAAAGACCAACCTCAAGACACCTCCAGAAAAGGT
     VIT_04s0008g03990_SV2     (321)  CTGTTGACACCCCCACTAAAGACCAACCTCAAGACACCTCCAGAAAAGGT
                 Consensus     (501)  CTG TGACACCCCCACTAAAGACCAACCTCAAGACACCTCCAGAAAAGGT
                                       551                                              600
GSVIVT00032490001-32491001     (327)  TACTTCTTGGAAAAAGACCTGCATTCCACCACAAAAATGAAGATGCACTT
         VIT_04s0008g03990     (316)  TACTTCTTGGAAAAAGACCTGCATTCCACCACAAAAATGAAGATGCACTT
         VIT_04s0008g04000       (1)  --------------------------------------------------
     VIT_04s0008g03990_SV1     (551)  TACTTCTTGGAAAAAGACCTGCATTCCACCACAAAAATGAAGATGCACTT
     VIT_04s0008g03990_SV2     (371)  TACTTCTTGGAAAAAGACCTGCATTCCACCACAAAAATGAAGATGCACTT
                 Consensus     (551)  TACTTCTTGGAAAAAGACCTGCATTCCACCACAAAAATGAAGATGCACTT
                                       601                                              650
GSVIVT00032490001-32491001     (377)  CAAAAAAACTACAAATGAAGCCACTTTCTTACCCCGTCAAGTGGCCGACT
         VIT_04s0008g03990     (366)  TAGAAAAACTACAAATGAAGCCACTTTGTTACCCCGTCAAGTGGCCGACT
         VIT_04s0008g04000       (1)  --------------------------------------------------
     VIT_04s0008g03990_SV1     (601)  CAAAAAAACTACAAATGAAGCCACTTTCTTACCCCGTCAAGTGGCCGACT
     VIT_04s0008g03990_SV2     (421)  CAAAAAAACTACAAATGAAGCCACTTTCTTACCCCGTCAAGTGGCCGACT
                 Consensus     (601)  CAAAAAAACTACAAATGAAGCCACTTTCTTACCCCGTCAAGTGGCCGACT
                                       651                                              700
GSVIVT00032490001-32491001     (427)  CCATACCCTTTTCATCTGACAAGTTCCCAGAAATTCTAAACCGGTTTTCA
         VIT_04s0008g03990     (416)  CCATACCCTTTTCATCTGACAAGTTCCCAGAAATTCTAAATCGGTTTTCA
         VIT_04s0008g04000       (1)  --------------------------------------------------
     VIT_04s0008g03990_SV1     (651)  CCATACCCTTTTCATCTGACAAGTTCCCAGAAATTCTAAACCGGTTTTCA
     VIT_04s0008g03990_SV2     (471)  CCATACCCTTTTCATCTGACAAGTTCCCAGAAATTCTAAACCGGTTTTCA
                 Consensus     (651)  CCATACCCTTTTCATCTGACAAGTTCCCAGAAATTCTAAACCGGTTTTCA
                                       701                                              750
GSVIVT00032490001-32491001     (477)  CTGAAACAAGATTCCGAGGAAGCTGAAATAATGAAGGAAACGATACGAGA
         VIT_04s0008g03990     (466)  CTTAAACAAGAGTCCAAGGAAGCTGAAATA--------------------
         VIT_04s0008g04000       (1)  ------------------------------ATGAAGAAAACGATACAAGA
     VIT_04s0008g03990_SV1     (701)  CTGAAACAAGATTCCGAGGAAGCTGAAATAATGAAGGAAACGATACAAGA
     VIT_04s0008g03990_SV2     (521)  CTGAAACAAGATTCCGAGGAAGCTGAAATAATGAAGGAAACGATACAAGA
                 Consensus     (701)  CTGAAACAAGATTCCGAGGAAGCTGAAATAATGAAGGAAACGATACAAGA
                                       751                                              800
GSVIVT00032490001-32491001     (527)  CTGTGAACAACCAGCCCTGGAAGGATATTCCAGGCTCTGTGCCACATCCT
         VIT_04s0008g03990     (496)  --------------------------------------------------
         VIT_04s0008g04000      (21)  TTGCGAACAACCAGCCCTGGAAGGAGATTCCAGGTTTTGTGCCACATCCT
     VIT_04s0008g03990_SV1     (751)  CTGTGAACAACCAGCCCTGGAAGGATATTCCAGGTTCTGTGCCACATCCT
     VIT_04s0008g03990_SV2     (571)  CTGTGAACAACCAGCCCTGGAAGGATATTCCAGGTTCTGTGCCACATCCT
                 Consensus     (751)  CTGTGAACAACCAGCCCTGGAAGGATATTCCAGGTTCTGTGCCACATCCT
                                       801                                              850
GSVIVT00032490001-32491001     (577)  TGGAATCCTTAATTGATTTCAGCATTTCAAAGCTTGGAAAAAACATCAAA
         VIT_04s0008g03990     (496)  --------------------------------------------------
         VIT_04s0008g04000      (71)  TGGAATCCTTAATTGATTTAAGCATTTCAAAGCTTGGAAAAAACATCAAA
     VIT_04s0008g03990_SV1     (801)  TGGAATCCTTAATTGATTTCAGCATTTCAAAGCTTGGAAAAAACATCAAA
     VIT_04s0008g03990_SV2     (621)  TGGAATCCTTAATTGATTTCAGCATTTCAAAGCTTGGAAAAAACATCAAA
                 Consensus     (801)  TGGAATCCTTAATTGATTTCAGCATTTCAAAGCTTGGAAAAAACATCAAA
                                       851                                              900
GSVIVT00032490001-32491001     (627)  CTAATCTCAAATGGAGTTGAAATGGGAAGCCAGGAATACGAACTCGGAGT
         VIT_04s0008g03990     (496)  --------------------------------------------------
         VIT_04s0008g04000     (121)  CTAATCTCAAATGGAGTTGAAATGGGAAGCCAAGAATACGAACTCGGAGT
     VIT_04s0008g03990_SV1     (851)  CTAATCTCAAATGGAGTTGAAATGGGAAGCCAGGAATACGAACTCGGAGT
     VIT_04s0008g03990_SV2     (671)  CTAATCTCAAATGGAGTTGAAATGGGAAGCCAGGAATACGAACTCGGAGT
                 Consensus     (851)  CTAATCTCAAATGGAGTTGAAATGGGAAGCCAGGAATACGAACTCGGAGT
                                       901                                              950
GSVIVT00032490001-32491001     (677)  GGGAGTGAAGGTGGTTGCAGACAAATCAGTGGTGTGCCATAAGCCAAAGT
         VIT_04s0008g03990     (496)  --------------------------------------------------
         VIT_04s0008g04000     (171)  GGGAGTGAAGGTGGTTGCAGACAAATCAGTGGTGTGCCATAAGCAGAAGT
     VIT_04s0008g03990_SV1     (901)  GGGAGTGAAGGTGGTTGCAGACAAATCAGTGGTGTGCCATAAGCAGAAGT
     VIT_04s0008g03990_SV2     (721)  GGGAGTGAAGGTGGTTGCAGACAAATCAGTGGTGTGCCATAAGCAGAAGT
                 Consensus     (901)  GGGAGTGAAGGTGGTTGCAGACAAATCAGTGGTGTGCCATAAGCAGAAGT
```

```
                                        951                                              1000
GSVIVT00032490001-32491001     (727)  ATCCATATGCTGTGTTTTACTGCCATGCAACCCCAAAAAAGAGGGTTTAC
         VIT_04s0008g03990     (496)  --------------------------------------------------
         VIT_04s0008g04000     (221)  ATCCATACGCTGTGTTTTACTGCCATGCAATCCATAAGACGAGGGTTTAC
     VIT_04s0008g03990_SV1     (951)  ATCCATACGCTGTGTTTTACTGCCATGCAACCCATAAGACGAGGGTTTAC
     VIT_04s0008g03990_SV2     (771)  ATCCATACGCTGTGTTTTACTGCCATGCAACCCATAAGACGAGGGTTTAC
                 Consensus     (951)  ATCCATACGCTGTGTTTTACTGCCATGCAACCCATAAGACGAGGGTTTAC
                                      1001                                             1050
GSVIVT00032490001-32491001     (777)  CCCCTTCCATTTGTGGGAACCGAGGATGGAACAAAAACTGAAGGTGTGGC
         VIT_04s0008g03990     (496)  --------------------------------------------------
         VIT_04s0008g04000     (271)  ACACTTCCATTTGTGGGAACCGAGGATGGAACCAAAGCTGAGGTTGTGGC
     VIT_04s0008g03990_SV1    (1001)  ACACTTCCATTTGTGGGAACCGAGGATGGAACCAAAGTTGAGGTTGTGGC
     VIT_04s0008g03990_SV2     (821)  ACACTTCCATTTGTGGGAACCGAGGATGGAACCAAAGTTGAGGTTGTGGC
                 Consensus    (1001)  ACACTTCCATTTGTGGGAACCGAGGATGGAACCAAAG TGAGGTTGTGGC
                                      1051                                             1100
GSVIVT00032490001-32491001     (827)  TTCTTGCCCTATAAATACCTTGGCTTGGAACCCCAAACCAGCGGCCTTTC
         VIT_04s0008g03990     (496)  --------------------------------------------------
         VIT_04s0008g04000     (321)  TTCTTGCCATATAGATATGTCGGCTTGGAACCCGAAGCATGCGACCTTTC
     VIT_04s0008g03990_SV1    (1051)  TTCTTGCCATATAGATACGTCGGCTTGGAACCCGAAACATGCGGCCTTTC
     VIT_04s0008g03990_SV2     (871)  TTCTTGCCATATAGATACGTCGGCTTGGAACCCGAAACATGCGGCCTTTC
                 Consensus    (1051)  TTCTTGCCATATAGATACGTCGGCTTGGAACCCGAAACATGCGGCCTTTC
                                      1101                                             1150
GSVIVT00032490001-32491001     (877)  AAGTGCCGAAAATTTAAACCGGAAACTGGCCCTGGTTTCCCATTTCCTTC
         VIT_04s0008g03990     (496)  --------------------------------------------------
         VIT_04s0008g04000     (371)  AAGTGCTGAAAG-TTAAACCAGGAACTGTCCCTG-TTTGCCACTTCCTTC
     VIT_04s0008g03990_SV1    (1101)  AAGTGCTGAAAG-TTAAACCAGGAACTGTCCCTG-TTTGCCACTTCCTTC
     VIT_04s0008g03990_SV2     (921)  AAGTGCTGAAAG-TTAAACCAGGAACTGTCCCTG-TTTGCCACTTCCTTC
                 Consensus    (1101)  AAGTGCTGAAAG-TTAAACCAGGAACTGTCCCTG TTTGCCACTTCCTTC
                                      1151                                             1200
GSVIVT00032490001-32491001     (927)  CCCCGGAAAAATCCAACCGGGGTTCCAAAATAGCCGGGAATACTGA----
         VIT_04s0008g03990     (496)  --------------------------------------------------
         VIT_04s0008g04000     (419)  CTCGTGATGA-TCTCATCTAGGTTCCTAAATAACTAGAAAT-CTGAAAGA
     VIT_04s0008g03990_SV1    (1149)  CTCGTGATGA-TCTCATCTGGGTTCCTAAATAGCTAGAAAT-CTGAAAGA
     VIT_04s0008g03990_SV2     (969)  CTCGTGATGA-TCTCATCTGGGTTCCTAAATAGCTAGAAAT-CTGAAAGA
                 Consensus    (1151)  CTCGTGATGA TCTCATCTGGGTTCCTAAATAGCTAGAAAT CTGAAAGA
                                      1201                                             1250
GSVIVT00032490001-32491001     (973)  --------------------------------------------------
         VIT_04s0008g03990     (496)  --------------------------------------------------
         VIT_04s0008g04000     (467)  AAAAAAAAAGCCTCAAGCCCAATCGCATAAGTCGATCTTGTATTTTCTAA
     VIT_04s0008g03990_SV1    (1197)  AAAAGAAAAACCTCAAGCCCAGTTGCATAAGTCGATCTTATATTTTCTAA
     VIT_04s0008g03990_SV2    (1017)  AAAAGAAAAACCTCAAGCCCAGTTGCATAAGTCGATCTTATATTTTCTAA
                 Consensus    (1201)  AAAA AAAA CCTCAAGCCCA T GCATAAGTCGATCTT TATTTTCTAA
                                      1251                                             1300
GSVIVT00032490001-32491001     (973)  --------------------------------------------------
         VIT_04s0008g03990     (496)  --------------------------------------------------
         VIT_04s0008g04000     (517)  TATCCATATCTGATGTGCTTTGAATAAGTGTTGGTTAGAGGAATGTATGT
     VIT_04s0008g03990_SV1    (1247)  TATCCATATCTGATGTGCTTTGAATAAGTGCTGGTTAGAGGAATGTATGT
     VIT_04s0008g03990_SV2    (1067)  TATCCATATCTGATGTGCTTTGAATAAGTGCTGGTTAGAGGAATGTATGT
                 Consensus    (1251)  TATCCATATCTGATGTGCTTTGAATAAGTG TGGTTAGAGGAATGTATGT
                                      1301                                             1350
GSVIVT00032490001-32491001     (973)  --------------------------------------------------
         VIT_04s0008g03990     (496)  --------------------------------------------------
         VIT_04s0008g04000     (567)  ATGTCTATGGGTACTATTTGC-----------------------------
     VIT_04s0008g03990_SV1    (1297)  ATGTCTATGGGCACTATTTGCAATGTAATTCGGGTTATGCCATGACCATT
     VIT_04s0008g03990_SV2    (1117)  ATGTCTATGGGCACTATTTGCAATGTAATTCGGGTTATGCCATGACCATT
                 Consensus    (1301)  ATGTCTATGGG ACTATTTGC
                                      1351                                             1400
GSVIVT00032490001-32491001     (973)  --------------------------------------------------
         VIT_04s0008g03990     (496)  --------------------------------------------------
         VIT_04s0008g04000     (588)  --------------------------------------------------
     VIT_04s0008g03990_SV1    (1347)  TTCTTGGTTTTTATCCATGTTTTCATAACTGGACTGGTCATTGAATGTGA
     VIT_04s0008g03990_SV2    (1167)  TTCTTGGTTTTTATCCATGTTTTCATAACTGGACTGGTCATTGAATG---
                 Consensus    (1351)
                                      1401       1417
GSVIVT00032490001-32491001     (973)  -----------------
         VIT_04s0008g03990     (496)  -----------------
         VIT_04s0008g04000     (588)  -----------------
     VIT_04s0008g03990_SV1    (1397)  AAAATTATCAATTTATG
     VIT_04s0008g03990_SV2    (1214)  -----------------
```

VvBURP07
Protein alignment

```
                                          1                                                50
  GSVIVT00032490001-32491001    (1)  ----------------MELHLLPILTCLSLLVVVSNASLPSEVYWKLALP
          VIT_04s0008g03990    (1)  ----------------MELHLLPILTCLSLLVVVNNASLPSEVYWKLALP
          VIT_04s0008g04000    (1)  --------------------------------------------------
   VIT_04s0008g03990_4000_SV2    (1)  MLTTTNVQLSLRFIASMELHLLPILTCLSLLVVVSNASLPSEVYWKLALP
  VIT_04s0008g03990_4000_SV1a    (1)  MLTTTNVQLSLRFIASMELHLLPILTCLSLLVVVSNASLPSEVYWKLALP
  VIT_04s0008g03990_4000_SV1b    (1)  --------------------------------------------------
                    Consensus    (1)                  MELHLLPILTCLSLLVVVSNASLPSEVYWKLALP
                                      51                                              100
  GSVIVT00032490001-32491001   (35)  YTPVPKAMRDLLQLNSMEGGSSINVSKVVLNAIPGPVYINCRVPPAADTP
          VIT_04s0008g03990   (35)  YTRMPKVVRDLLQLNSMEGGSSINVSKVILNAIPADVFIKYQNPSAADTP
          VIT_04s0008g04000    (1)  --------------------------------------------------
   VIT_04s0008g03990_4000_SV2   (51)  YTPMPKAMRDLLQLNSMEGGSSINVSKVVLNAIPGPVYINCRVPPAVDTP
  VIT_04s0008g03990_4000_SV1a   (51)  YTPMPKAMRDLLQLS-----------------------------------
  VIT_04s0008g03990_4000_SV1b    (1)  ----------------MEGGSSINVSKVVLNAIPGPVYINCRVPPAVDTP
                    Consensus   (51)  YTPMPKAMRDLLQLNSMEGGSSINVSKVVLNAIPGPVYINCRVPPA DTP
                                      101                                             150
  GSVIVT00032490001-32491001   (85)  TKDQPQDTSRKGYFLEKDLHSTTKMKMHFKKTTNEATFLPRQVADSIPFS
          VIT_04s0008g03990   (85)  TEDQPPEKVTSWKKTCIPPQK-----------------------------
          VIT_04s0008g04000    (1)  --------------------------------------------------
   VIT_04s0008g03990_4000_SV2  (101)  TKDQPQDTSRKGYFLEKDLHSTTKMKMHFKKTTNEATFLPRQVADSIPFS
  VIT_04s0008g03990_4000_SV1a   (66)  --------------------------------------------------
  VIT_04s0008g03990_4000_SV1b   (35)  TKDQPQDTSRKGYFLEKDLHSTTKMKMHFKKTTNEATFLPRQVADSIPFS
                    Consensus  (101)  TKDQPQDTSRKGYFLEKDLHSTTKMKMHFKKTTNEATFLPRQVADSIPFS
                                      151                                             200
  GSVIVT00032490001-32491001  (135)  SDKFPEILNRFSLKQDSEEAEIMKETIRDCEQPALEGYSRLCATSLESLI
          VIT_04s0008g03990  (106)  --------------------------------------------------
          VIT_04s0008g04000    (1)  ----------------------MKKTIQDCEQPALEGDSRFCATSLESLI
   VIT_04s0008g03990_4000_SV2  (151)  SDKFPEILNRFSLKQDSEEAEIMKETIQDCEQPALEGYSRFCATSLESLI
  VIT_04s0008g03990_4000_SV1a   (66)  --------------------------------------------------
  VIT_04s0008g03990_4000_SV1b   (85)  SDKFPEILNRFSLKQDSEEAEIMKETIQDCEQPALEGYSRFCATSLESLI
                    Consensus  (151)  SDKFPEILNRFSLKQDSEEAEIMKETIQDCEQPALEGYSRFCATSLESLI
                                      201                                             250
  GSVIVT00032490001-32491001  (185)  DFSISKLGKNIKLISNGVEMGSQEYELGVGVKVVADKSVVCHKPKYPYAV
          VIT_04s0008g03990  (106)  --------------------------------------------------
          VIT_04s0008g04000   (29)  DLSISKLGKNIKLISNGVEMGSQEYELGVGVKVVADKSVVCHKQKYPYAV
   VIT_04s0008g03990_4000_SV2  (201)  DFSISKLGKNIKLISNGVEMGSQEYELGVGVKVVADKSVVCHKQKYPYAV
  VIT_04s0008g03990_4000_SV1a   (66)  --------------------------------------------------
  VIT_04s0008g03990_4000_SV1b  (135)  DFSISKLGKNIKLISNGVEMGSQEYELGVGVKVVADKSVVCHKQKYPYAV
                    Consensus  (201)  DFSISKLGKNIKLISNGVEMGSQEYELGVGVKVVADKSVVCHKQKYPYAV
                                      251                                             300
  GSVIVT00032490001-32491001  (235)  FYCHATPKKRVYPLPFVGTEDGTKTEGVASCPINTLAWNPKPAAFQVPKI
          VIT_04s0008g03990  (106)  --------------------------------------------------
          VIT_04s0008g04000   (79)  FYCHAIHKTRVYTLPFVGTEDGTKAEVVASCHIDMSAWNPKHATFQVLKV
   VIT_04s0008g03990_4000_SV2  (251)  FYCHATHKTRVYTLPFVGTEDGTKVEVVASCHIDTSAWNPKHAAFQVLKV
  VIT_04s0008g03990_4000_SV1a   (66)  --------------------------------------------------
  VIT_04s0008g03990_4000_SV1b  (185)  FYCHATHKTRVYTLPFVGTEDGTKVEVVASCHIDTSAWNPKHAAFQVLKV
                    Consensus  (251)  FYCHATHKTRVYTLPFVGTEDGTK EVVASCHIDTSAWNPKHAAFQVLKV
                                      301                 321
  GSVIVT00032490001-32491001  (285)  ---------------------
          VIT_04s0008g03990  (106)  ---------------------
          VIT_04s0008g04000  (129)  KPGTVPVCHFLPRDDLI----
   VIT_04s0008g03990_4000_SV2  (301)  KPGTVPVCHFLPRDDLIWVPK
  VIT_04s0008g03990_4000_SV1a   (66)  ---------------------
  VIT_04s0008g03990_4000_SV1b  (235)  KPGTVPVCHFLPRDDLIWVPK
                    Consensus  (301)  KPGTVPVCHFLPRDDLI
```

VvBURP08
Protein alignment

```
                                  1                                                50
        GSVIVP00032493001    (1) --------------------------------------------------
VIT_04s0008g04010_CRIBI      (1) MPIKAILIEGQDIIPSCGLKTRPLNGWGGYGLLVSLALICSFSRMRSHLN
  VIT_04s0008g04010_SV1      (1) --------------------------------------------------
              Consensus      (1)
                                  51                                              100
        GSVIVP00032493001    (1) --------------------------------------------------
VIT_04s0008g04010_CRIBI     (51) LGDVKPEDVSEDTLKAVAEILRNSTTLKVSEDGKKIGRATELLKPEEVVE
  VIT_04s0008g04010_SV1      (1) --------------------------------------------------
              Consensus     (51)
                                  101                                             150
        GSVIVP00032493001    (1) --------------------------------------------------
VIT_04s0008g04010_CRIBI    (101) QVNIRTIAASPLEYDAKLEDVEAYFGQIAKVNSVRLPRHVAEKRVFCGTA
  VIT_04s0008g04010_SV1      (1) --------------------------------------------------
              Consensus    (101)
                                  151                                             200
        GSVIVP00032493001    (1) --------------------------------------------------
VIT_04s0008g04010_CRIBI    (151) LIQYSTEEDAAKVLQQSLVYAEGLISVDALAPSCIGVHVHFDKFSQPAGE
  VIT_04s0008g04010_SV1      (1) --------------------------------------------------
              Consensus    (151)
                                  201                                             250
        GSVIVP00032493001    (1) --------------------------------------------------
VIT_04s0008g04010_CRIBI    (201) EAQVDDSKIGNFFLETDLHPGKKMKLNLATTTNGAVFLPHQVAESMPFSS
  VIT_04s0008g04010_SV1      (1) -----------------------MKLNLATTTNGAVFLPHQVAESMPFSS
              Consensus    (201)                        MKLNLATTTNGAVFLPHQVAESMPFSS
                                  251                                             300
        GSVIVP00032493001    (1) --------------------MMLHSTWLLLPWQAPHRPLAPSLLSQLLRI
VIT_04s0008g04010_CRIBI    (251) NKLPEILNRFS---------------------------------------
  VIT_04s0008g04010_SV1     (28) NKLPEILNRFSLKENSAEAEIIKKELEECEEPAMEGEARYCATSLQSLIH
              Consensus    (251) NKLPEILNRFS         II                   A   Q L
                                  301                                             350
        GSVIVP00032493001   (31) FLSGKIGSRFWVACWMKRAWIQQLDSQEYEFGVGMKKVADKSVVCHKMNY
VIT_04s0008g04010_CRIBI    (262) --------------------------------------------------
  VIT_04s0008g04010_SV1     (78) FSTSKLGRNVNVLTNEVKTG-----SQEYEFGVGMKRVADKSVVCHKMNY
              Consensus    (301) F S KIG    V     K       SQEYEFGVGMKKVADKSVVCHKMNY
                                  351                                             400
        GSVIVP00032493001   (81) PYAVFYCHTFTKTRTYMIPLVGVDGSKSKAMAACHSDTSAWHPQHVAFQV
VIT_04s0008g04010_CRIBI    (262) --------------------------------------------------
  VIT_04s0008g04010_SV1    (123) PYAVFYCHTFTKTRTYMIPLVGADGSKAKAMAACHSDTSAWHPQHVAFQV
              Consensus    (351) PYAVFYCHTFTKTRTYMIPLVG DGSKAKAMAACHSDTSAWHPQHVAFQV
                                  401                     424
        GSVIVP00032493001  (131) LKIKPGTVPVCHFLHNNAMVWIPK
VIT_04s0008g04010_CRIBI    (262) ------------------------
  VIT_04s0008g04010_SV1    (173) LKIKPGTVAVYHFLHNNAMVWIPK
              Consensus    (401) LKIKPGTV V HFLHNNAMVWIPK
```

VvBURP09
DNA alignment

```
                                  1                                                 50
VIT_04s0008g04020_CRIBI     (1) --------------------------------------------------
  VIT_04s0008g04020_SV1     (1) CAAGCTTCTTGTCTCAATTTCTGAGTTTCTGAGCTTTTACCATTAATGGA
  VIT_04s0008g04020_SV2     (1) CAAGCTTCTTGTCTCAATTTCTGAGTTTCTGAGCTTTTACCATTAATGGA
  VIT_04s0008g04020_SV3     (1) --------------------------------------------------
              Consensus     (1) CAAGCTTCTTGTCTCAATTTCTGAGTTTCTGAGCTTTTACCATTAATGGA
                                51                                               100
VIT_04s0008g04020_CRIBI     (1) ----CTCCATCCTCTTCCCATTCTGGCTTTTCTTTCGGAGGTGGTGGTAG
  VIT_04s0008g04020_SV1    (51) GTTCCTTCGTCCTCTTCCCATTCTGGCTTTTCTTTCAGTGGTGGTGGTAG
  VIT_04s0008g04020_SV2    (51) GTTCCTTCGTCCTCTTCCCATTCTGGCTTTTCTTTCAGTGGTGGTGGTAG
  VIT_04s0008g04020_SV3     (1) --------------------------------------------------
              Consensus    (51) GTTCCTTCGTCCTCTTCCCATTCTGGCTTTTCTTTCAGTGGTGGTGGTAG
                                101                                              150
VIT_04s0008g04020_CRIBI    (47) TAAGCCATGCTTCTCTACCTTCTGAAGGTTACTGGAAGTTGGTTTTGTCT
  VIT_04s0008g04020_SV1   (101) TAAGCCATGCTTCTCTACCTTCTGAGGGTTACTGGAAGTTGGTTTTGCCT
  VIT_04s0008g04020_SV2   (101) TAAGCCATGCTTCTCTACCTTCTGAGGGTTACTGGAAGTTGGTTTTGCCT
  VIT_04s0008g04020_SV3     (1) --------------------------------------------------
              Consensus   (101) TAAGCCATGCTTCTCTACCTTCTGAGGGTTACTGGAAGTTGGTTTTGCCT
                                151                                              200
VIT_04s0008g04020_CRIBI    (97) CATACTCCCATGCCCAAAGCTGTGAAGGATCACTTGCAGCCTGGCGTA--
  VIT_04s0008g04020_SV1   (151) CACACTCCCATGCCCAAAGCTGTGAAGGATCACTTGCAGCCTGGTGTA--
  VIT_04s0008g04020_SV2   (151) CACACTCCCATGCCCAAAGCTGTGAAGGATCACTTGCAGCCTGGTGTA--
  VIT_04s0008g04020_SV3     (1) -----TCCCATGCCCAAAGCTGTGAAGGATCACTTGCAGCCTGGTTTGTT
              Consensus   (151) CACACTCCCATGCCCAAAGCTGTGAAGGATCACTTGCAGCCTGGTGTA
                                201                                              250
VIT_04s0008g04020_CRIBI   (145) -----ACTTCTTT-----CAATG----CTTC--TGCTGCCAGATGTTTTG
  VIT_04s0008g04020_SV1   (199) -----ACTTCTTT-----CAATG-CTTCTGC--TGCTGCTTTTGTTTGG
  VIT_04s0008g04020_SV2   (199) -----ACTTCTTT-----CAATG-CTTCTGC--TGCTGCTTTTGTTTGG
  VIT_04s0008g04020_SV3    (46) TCAATACATCTCTAGACTCAGTGGCAACATCCTTTCTTTTAGATGTTTTG
              Consensus   (201)      ACTTCTTT     CAATG CTTCT C  TGCTGCT   TGTTT G
                                251                                              300
VIT_04s0008g04020_CRIBI   (179) GGATTCACAATCAGTTCAACAAAATCCCTCAACCATTCCATGCAAAGCAG
  VIT_04s0008g04020_SV1   (236) GGATTTACTATGGGTTCAACAAAATCCCTCAACCATTCCATGCAAAGCAG
  VIT_04s0008g04020_SV2   (236) GGATTTACTATGGGTTCAACAAAATCCCTCAACCATTCCATGCAAAGCAG
  VIT_04s0008g04020_SV3    (96) GGATTCACAATCAGTTCAACAAAATCCCTCAACCATTCCATGCAAAGCAG
              Consensus   (251) GGATT AC AT  GTTCAACAAAATCCCTCAACCATTCCATGCAAAGCAG
                                301                                              350
VIT_04s0008g04020_CRIBI   (229) GCTCAAGATGGCTTAAATATAGGTAACTTCTTCTTGCAAACAGATCTGCA
  VIT_04s0008g04020_SV1   (286) GCCCAAGATGGCTTAAATATAGGTAACTTCTTCTTGCAAACAGATCTGCA
  VIT_04s0008g04020_SV2   (286) GCCCAAGATGGCTTAAATATAGGTAACTTCTTCTTGCAAACAGATCTGCA
  VIT_04s0008g04020_SV3   (146) GCTCAAGATGGCTTAAATATAGGTAACTTCTTCTTGCAAACAGATCTGCA
              Consensus   (301) GC CAAGATGGCTTAAATATAGGTAACTTCTTCTTGCAAACAGATCTGCA
                                351                                              400
VIT_04s0008g04020_CRIBI   (279) CCCAGGTACAAAAATGATGCTGCAGTTGCCACAAAGTACAAATGAAGGTA
  VIT_04s0008g04020_SV1   (336) CCCAGGTACAAAAATGATGCTGCAGTTGCCACAAAGTACAAATGAAGGTA
  VIT_04s0008g04020_SV2   (336) CCCAGGTACAAAAATGATGCTGCAGTTGCCACAAAGTACAAATGAAG---
  VIT_04s0008g04020_SV3   (196) CCCAGGTACAAAAATGATGCTGCAGTTGCCACAAAGTACAAATGAAG---
              Consensus   (351) CCCAGGTACAAAAATGATGCTGCAGTTGCCACAAAGTACAAATGAAGGTA
                                401                                              450
VIT_04s0008g04020_CRIBI   (329) TGTTCTTGCCTTGTCAAGTTGCTGACTCCATATCCTTTTCATCTAAGAAG
  VIT_04s0008g04020_SV1   (386) TGTTCTTGCCTTGTCAAGTTGCTGACTCCATATCCTTTTCATCTAAGAAG
  VIT_04s0008g04020_SV2   (383) --------------------------------------------------
  VIT_04s0008g04020_SV3   (243) --------------------------------------------------
              Consensus   (401) TGTTCTTGCCTTGTCAAGTTGCTGACTCCATATCCTTTTCATCTAAGAAG
                                451                                              500
VIT_04s0008g04020_CRIBI   (379) TTACCCAAAATTTTGAACCGGCTTTCAGTGAAGGAAAAATCTGCAGAAGC
  VIT_04s0008g04020_SV1   (436) TTACCCAAAATTTTGAACCGGCTTTCAGTGAAGGAAAAATCTGCAGAAGC
  VIT_04s0008g04020_SV2   (383) ----------------------------TGAAGGAAAAATCTGCAGAAGC
  VIT_04s0008g04020_SV3   (243) ----------------------------TGAAGGAAAAATCTGCAGAAGC
              Consensus   (451) TTACCCAAAATTTTGAACCGGCTTTCAGTGAAGGAAAAATCTGCAGAAGC
                                501                                              550
VIT_04s0008g04020_CRIBI   (429) TGAGCTGATGAAGAAGGAGATAGAAGAGCTAGTGTGAGGAGGCTGCCATG
  VIT_04s0008g04020_SV1   (486) TGAGCTGATGAAGAAGGAGATAGAAGAGCTAGTGTGAGGAGGCTGCCATG
  VIT_04s0008g04020_SV2   (405) TGAGCTGATGAAGAAGGAGATAGAAGAGCTAGTGTGAGGAGGCTGCCATG
  VIT_04s0008g04020_SV3   (265) TGAGCTGATGAAGAAG----------------------------------
              Consensus   (501) TGAGCTGATGAAGAAGGAGATAGAAGAGCTAGTGTGAGGAGGCTGCCATG
```

```
                                    551                                               600
VIT_04s0008g04020_CRIBI     (479)   GATGGAGAATCTAGGTTCTGTGCAACATCATTAGAGTCCCTAATCGATTT
  VIT_04s0008g04020_SV1     (536)   GATGGAGAATCTAGGTTCTGTGCAACATCATTAGAGTCCCTAATCGATTT
  VIT_04s0008g04020_SV2     (455)   GATGGAGAATCTAGGTTCTGTGCAACATCATTAGAGTCCCTAATCGATTT
  VIT_04s0008g04020_SV3     (281)   --------------------------------------------------
              Consensus     (551)   GATGGAGAATCTAGGTTCTGTGCAACATCATTAGAGTCCCTAATCGATTT
                                    601                                               650
VIT_04s0008g04020_CRIBI     (529)   CAGCACTTCAAAGCTCGGAAGAAATGTGAATGTGCTGACGAATGAGGTCA
  VIT_04s0008g04020_SV1     (586)   CAGCACTTCAAAGCTCGGAAGAAATGTGAATGTGCTGACGAATGAGGTCA
  VIT_04s0008g04020_SV2     (505)   CAGCACTTCAAAGCTCGGAAGAAATGTGAATGTGCTGACGAATGAGGTCA
  VIT_04s0008g04020_SV3     (281)   --------------------------------------------------
              Consensus     (601)   CAGCACTTCAAAGCTCGGAAGAAATGTGAATGTGCTGACGAATGAGGTCA
                                    651                                               700
VIT_04s0008g04020_CRIBI     (579)   AAACGGGAAGCCAAGAGTATGAATTTGGAGTGGGAATGAAGAAGGTTGCA
  VIT_04s0008g04020_SV1     (636)   AAACGGGAAGCCAAGAGTATGAATTTGGAGTGGGAATGAAGAGGGTTGCA
  VIT_04s0008g04020_SV2     (555)   AAACGGGAAGCCAAGAGTATGAATTTGGAGTGGGAATGAAGAGGGTTGCA
  VIT_04s0008g04020_SV3     (281)   --------------------------------------------------
              Consensus     (651)   AAACGGGAAGCCAAGAGTATGAATTTGGAGTGGGAATGAAGAGGGTTGCA
                                    701                                               750
VIT_04s0008g04020_CRIBI     (629)   GACAAATCAGTGGTGTGCCATAAGATGAACTACCCATATGCTGTTTTCTA
  VIT_04s0008g04020_SV1     (686)   GACAAATCAGTGGTGTGCCATAAGATGAACTACCCATATGCTGTTTTCTA
  VIT_04s0008g04020_SV2     (605)   GACAAATCAGTGGTGTGCCATAAGATGAACTACCCATATGCTGTTTTCTA
  VIT_04s0008g04020_SV3     (281)   --------------------------------------------------
              Consensus     (701)   GACAAATCAGTGGTGTGCCATAAGATGAACTACCCATATGCTGTTTTCTA
                                    751                                               800
VIT_04s0008g04020_CRIBI     (679)   TTGCCATACCTTCACTAAGACACGAACTTACATGATTCCATTGGTGGGTG
  VIT_04s0008g04020_SV1     (736)   CTGCCATACATTCACTAAGACACGGACTTACATGATTCCGTTGGTGGGTG
  VIT_04s0008g04020_SV2     (655)   CTGCCATACATTCACTAAGACACGGACTTACATGATTCCGTTGGTGGGTG
  VIT_04s0008g04020_SV3     (281)   --------------------------------------------------
              Consensus     (751)   CTGCCATACATTCACTAAGACACGGACTTACATGATTCCGTTGGTGGGTG
                                    801                                               850
VIT_04s0008g04020_CRIBI     (729)   CTGATGGAAGCAAAGCTAAAGCCATGGCAGCTTGTCACAGTGATACATCA
  VIT_04s0008g04020_SV1     (786)   CTGATGGAAGCAAAGCTAAAGCCATGGCAGCTTGTCATAGTGATACATCA
  VIT_04s0008g04020_SV2     (705)   CTGATGGAAGCAAAGCTAAAGCCATGGCAGCTTGTCATAGTGATACATCA
  VIT_04s0008g04020_SV3     (281)   --------------------------------------------------
              Consensus     (801)   CTGATGGAAGCAAAGCTAAAGCCATGGCAGCTTGTCATAGTGATACATCA
                                    851                                               900
VIT_04s0008g04020_CRIBI     (779)   GCTTGGCACCCACAGCATGTGGCTTTCCAAGTGCTCAAAATTAAGCCAGG
  VIT_04s0008g04020_SV1     (836)   GCTTGGCACCCAAAACATGTGGCCTTCAAAGTGCTCAATGTTAAGCCAGG
  VIT_04s0008g04020_SV2     (755)   GCTTGGCACCCAAAACATGTGGCCTTCAAAGTGCTCAATGTTAAGCCAGG
  VIT_04s0008g04020_SV3     (281)   --------------------------------------------------
              Consensus     (851)   GCTTGGCACCCAAAACATGTGGCCTTCAAAGTGCTCAATGTTAAGCCAGG
                                    901                                               950
VIT_04s0008g04020_CRIBI     (829)   AACGGTCGCAGTCTATCATTTCCTTCACAACAATGCCATGGTCTGGATTC
  VIT_04s0008g04020_SV1     (886)   AACAGTCCCTATCTGCCATTTCGTTCACAACAATGCCATGGTCTGGATTC
  VIT_04s0008g04020_SV2     (805)   AACAGTCCCTATCTGCCATTTCGTTCACAACAATGCCATGGTCTGGATTC
  VIT_04s0008g04020_SV3     (281)   --------------------------------------------------
              Consensus     (901)   AACAGTCCCTATCTGCCATTTCGTTCACAACAATGCCATGGTCTGGATTC
                                    951                                              1000
VIT_04s0008g04020_CRIBI     (879)   CAAAGTAAGAATCAGCCTCAAGCCTCAAGCCCAGTTGCATTATCAGTTCC
  VIT_04s0008g04020_SV1     (936)   CAAAATAGCCTGATCATGAAATCAACGGAAATATTTAATCCTCAAGCCCA
  VIT_04s0008g04020_SV2     (855)   CAAAATAGCCTGATCATGAAATCAACGGAAATATTTAATCCTCAAGCCCA
  VIT_04s0008g04020_SV3     (281)   --------------------------------------------------
              Consensus     (951)   CAAAATAGCCTGATCATGAAATCAACGGAAATATTTAATCCTCAAGCCCA
                                    1001                                             1050
VIT_04s0008g04020_CRIBI     (929)   ATCTTAAAGCTGTTTGTAGTATGAATGGTTCGAATAAATGCAGGTTCGAG
  VIT_04s0008g04020_SV1     (986)   GTTGCATTGTTCCATGTAATAGTCGTTTCTAGTATCAATGGTGTGGTTTG
  VIT_04s0008g04020_SV2     (905)   GTTGCATTGTTCCATGTAATAGTCGTTTCTAGTATCAATGGTGTGGTTTG
  VIT_04s0008g04020_SV3     (281)   --------------------------------------------------
              Consensus    (1001)   GTTGCATTGTTCCATGTAATAGTCGTTTCTAGTATCAATGGTGTGGTTTG
                                    1051                                             1100
VIT_04s0008g04020_CRIBI     (979)   GATTAATGTGTGTAGTGTGCAATTGATGCTATG---------TATTCATC
  VIT_04s0008g04020_SV1    (1036)   AATAAATGCAGGTTAATTTGAGAAGAATGTAGTGTGGAATGCTATTCATC
  VIT_04s0008g04020_SV2     (955)   AATAAATGCAGGTTAATTTGAGAAGAATGTAGTGTGGAATGCTATTCATC
  VIT_04s0008g04020_SV3     (281)   --------------------------------------------------
              Consensus    (1051)   AATAAATGCAGGTTAATTTGAGAAGAATGTAGTGTGGAATGCTATTCATC
```

```
                                   1101                                              1150
VIT_04s0008g04020_CRIBI    (1020)  AATGTTTCCAGGTTGATTAATTAAGGAGTATTATGGTATATATATATATA
  VIT_04s0008g04020_SV1    (1086)  AATCTTTCCCGGTTGATTTAAGGAATATTGTAAATATTGTTGTGTGGTTT
  VIT_04s0008g04020_SV2    (1005)  AATCTTTCCCGGTTGATTTAAGGAATATTGTAAATATTGTTGTGTGGTTT
  VIT_04s0008g04020_SV3     (281)  --------------------------------------------------
              Consensus    (1101)  AATCTTTCCCGGTTGATTTAAGGAATATTGTAAATATTGTTGTGTGGTTT

                                   1151                          1181
VIT_04s0008g04020_CRIBI    (1070)  TA-----------------------------
  VIT_04s0008g04020_SV1    (1136)  GAATAAATGCAGGTTAATTTGAGAAGAATGT
  VIT_04s0008g04020_SV2    (1055)  GAATAAATGCAGGTTAATTTGAGAAGAATGT
  VIT_04s0008g04020_SV3     (281)  -------------------------------
              Consensus    (1151)  GAATAAATGCAGGTTAATTTGAGAAGAATGT
```

VvBURP09
Protein alignment

```
                                     1                                                 50
GSVIVP00032495001-32596001    (1)  MPKAVKDHLRPGVTSFNASAAGCFGIHNQFNKIPQPFHAKQAQDGLNTGN
         VIT_04s0008g04020    (1)  --------------------------------------------------
     VIT_04s0008g04020_SV1    (1)  --------------------------------------------------
     VIT_04s0008g04020_SV2    (1)  --------------------------------------------------
                 Consensus    (1)
                                   51                                               100
GSVIVP00032495001-32596001   (51)  FFLQTDLHPGTKMMLQLPQTRNEAMFLPRQVADSIPFSSKKLPEILNRLS
         VIT_04s0008g04020    (1)  --------------------------------------------------
     VIT_04s0008g04020_SV1    (1)  --------------------------------------------------
     VIT_04s0008g04020_SV2    (1)  --------------------------------------------------
                 Consensus   (51)
                                   101                                              150
GSVIVP00032495001-32596001  (101)  VKEKSAEAELMKEEIEECEEDAMDGESRFCATSLESLIDFSTSKLGRNVN
         VIT_04s0008g04020    (1)  ----------------------MDGESRFCATSLESLIDFSTSKLGRNVN
     VIT_04s0008g04020_SV1    (1)  ----------------------MDGESRFCATSLESLIDFSTSKLGRNVN
     VIT_04s0008g04020_SV2    (1)  ----------------------MDGESRFCATSLESLIDFSTSKLGRNVN
                 Consensus  (101)                        MDGESRFCATSLESLIDFSTSKLGRNVN
                                   151                                              200
GSVIVP00032495001-32596001  (151)  VLTNEVKTGSQEYEFGVGMKKVADKSVVCHKMNYPYAVFYCHTFTKARTY
         VIT_04s0008g04020   (29)  VLTNEVKTGSQEYEFGVGMKKVADKSVVCHKMNYPYAVFYCHTFTKTRTY
     VIT_04s0008g04020_SV1   (29)  VLTNEVKTGSQEYEFGVGMKRVADKSVVCHKMNYPYAVFYCHTFTKTRTY
     VIT_04s0008g04020_SV2   (29)  VLTNEVKTGSQEYEFGVGMKRVADKSVVCHKMNYPYAVFYCHTFTKTRTY
                 Consensus  (151)  VLTNEVKTGSQEYEFGVGMK VADKSVVCHKMNYPYAVFYCHTFTKTRTY
                                   201                                              250
GSVIVP00032495001-32596001  (201)  MIPLVGVDGSKAKAMAACHSDTSAWHPQHVALPSAQN-------------
         VIT_04s0008g04020   (79)  MIPLVGADGSKAKAMAACHSDTSAWHPQHVAFQVLKIKPGTVAVYHFLHN
     VIT_04s0008g04020_SV1   (79)  MIPLVGADGSKAKAMAACHSDTSAWHPKHVAFKVLNVKPGTVPICHFVHN
     VIT_04s0008g04020_SV2   (79)  MIPLVGADGSKAKAMAACHSDTSAWHPKHVAFKVLNVKPGTVPICHFVHN
                 Consensus  (201)  MIPLVGADGSKAKAMAACHSDTSAWHP HVAFKVLNVKPGTVPICHFVHN
                                   251
GSVIVP00032495001-32596001  (238)  --------
         VIT_04s0008g04020  (129)  NAMVWIPK
     VIT_04s0008g04020_SV1  (129)  NAMVWIPK
     VIT_04s0008g04020_SV2  (129)  NAMVWIPK
                 Consensus  (251)  NAMVWIPK
```

VvBURP12
Protein alignment

```
                                1                                                50
                                                  ↓
      GSVIVP00032501001    (1) --------------------------------------------------
      VIT_04s0008g04060    (1) MEFRSFPFLAFLSVMVVVIHASQPDEDYWKLALPHTPMPKAIRDLLQSKG
  VIT_04s0008g04060_SV1    (1) -------MVACMQVMVVVIHASQPDEDYWKLALPHTPMPKAIRDLLQSKG
              Consensus    (1)         LA L VMVVVIHASQPDEDYWKLALPHTPMPKAIRDLLQSKG

                               51                                               100
      GSVIVP00032501001    (1) -------------------------------------------------M
      VIT_04s0008g04060   (51) LSSVDASTASCTVHIHFDKFSQPAGEEAQVDDSKIGNFFLETDLHPGKKM
  VIT_04s0008g04060_SV1   (44) LSSVDASTASCTVHIHFDKFSQPAGEEAQVDDSKIGNFFLETDLHPGKKM
              Consensus   (51) LSSVDASTASCTVHIHFDKFSQPAGEEAQVDDSKIGNFFLETDLHPGKKM

                               101                                              150
      GSVIVP00032501001    (2) KLNLATTTNGAVFLPHQVAESMPFSSNKLPEILNRFSLKENSAEAEIIKK
      VIT_04s0008g04060  (101) KLNLATTTNGAVFLPHQVAESMPFSSNKLPEILNRFSLKENSAEAEIIKK
  VIT_04s0008g04060_SV1   (94) KLNLATTTNGAVFLPHQVAESMPFSS------------------------
              Consensus  (101) KLNLATTTNGAVFLPHQVAESMPFSSNKLPEILNRFSLKENSAEAEIIKK

                               151                                              200
      GSVIVP00032501001   (52) ELEECEEPAMEGEARYCATSLQSLIHFSTSKLGRNVNVLTNEVKTGSQEY
      VIT_04s0008g04060  (151) ELEECEEPAMEGEARYCATSLQSLIHFSTSKLGRNVNVLTNEVKTGSQEY
  VIT_04s0008g04060_SV1  (120) --------------------------------------------------
              Consensus  (151) ELEECEEPAMEGEARYCATSLQSLIHFSTSKLGRNVNVLTNEVKTGSQEY

                               201                                              250
      GSVIVP00032501001  (102) EFGVGMKRVADKSVVCHKMNYPYAVFYCHTFTKTRTYMIPLVGADGSKAK
      VIT_04s0008g04060  (201) EFGVGMKRVADKSVVCHKMNYPYAVFYCHTFTKTRTYMIPLVGADGSKAK
  VIT_04s0008g04060_SV1  (120) --------------------------------------------------
              Consensus  (201) EFGVGMKRVADKSVVCHKMNYPYAVFYCHTFTKTRTYMIPLVGADGSKAK

                               251                                       295
      GSVIVP00032501001  (152) AMAACHSDTSAWHPKHVAFKVLNVKPGTVPICHFVHNNAMVWIPK
      VIT_04s0008g04060  (251) AMAACHSDTSAWHPKHVAFKVLNVKPGTVPICHFVHNNAMVWIPK
  VIT_04s0008g04060_SV1  (120) ---------------------------------------------
              Consensus  (251) AMAACHSDTSAWHPKHVAFKVLNVKPGTVPICHFVHNNAMVWIPK
```

Arrow: possible translation start site

VvBURP14
Protein alignment

```
                                 1                                                50
    GSVIVP00032505001     (1) --------------------------------------------------
    VIT_04s0008g04120     (1) MTGGDNSQFSLVTSNGYCEGVLAPPDRGTSLELFPSHLSHIPLQLESFHG
VIT_04s0008g04110_SV1     (1) --------------------------------------------------
                              51                                              100
    GSVIVP00032505001     (1) --------------------------------------------------
    VIT_04s0008g04120    (51) VLPPSPSGISLPIIYIHQLISFLHTNVQLSLRFTASMELHLLPILTCLSV
VIT_04s0008g04110_SV1     (1) --------------------------------------------------
                              101                                             150
    GSVIVP00032505001     (1) --------------------------------------------------
    VIT_04s0008g04120   (101) RLRFYLSFVFIFLTTQKQGDASPIAGYDAVYGSTQKQRDASPIAGYDAVY
VIT_04s0008g04110_SV1     (1) --------------------------------------------------
                              151                                             200
    GSVIVP00032505001     (1) --------------------------------------------------
    VIT_04s0008g04120   (151) GSTQKQGDASPIAGYDAVYGSTKKQGDASPIAGYDAVYGSTKKQGNASPI
VIT_04s0008g04110_SV1     (1) --------------------------------------------------
                              201                                             250
    GSVIVP00032505001     (1) --------------------------------------------------
    VIT_04s0008g04120   (201) AGYDAVYGSTRKQGDASPIAGYAAVYGSTKKQGDASPISGYDAVYGTSQK
VIT_04s0008g04110_SV1     (1) --------------------------------------------------
                              251                                             300
    GSVIVP00032505001     (1) --------------------------------------MQYERVYGNPQK
    VIT_04s0008g04120   (251) AEGSSINVHKDASSIAGYDAVYGTPKKAIESSTKAHKDASQIAGYDAVYG
VIT_04s0008g04110_SV1     (1) --------------------------------------------------
                              301                                             350
    GSVIVP00032505001    (13) AGGSSVSAGKNSPNLQQGHQDGTSTGNVFLEKDLHPGTKMMVRFTKTSSA
    VIT_04s0008g04120   (301) NSQQKAGGSSINAHKDSSPVASYGAVNVFLEKDLHPGTKMAVRFTKTSSA
VIT_04s0008g04110_SV1     (1) ---------------------------------------MAVRFTKTSSA
                              351                                             400
    GSVIVP00032505001    (63) AHFLPQQVAESIPFSSNKLPEILNQFSVKENSAEAKIIQKTIEECEKPAI
    VIT_04s0008g04120   (351) AHFLPHQVAESIPFSSNKLPEILKRFSVKENSAEAQMIQKTIKQCETPAI
VIT_04s0008g04110_SV1    (12) AHFLPHQVAESIPFSSNKLPEILKRFSVKENSAEAQMIQKTIKQCETPAI
                              401                                             450
    GSVIVP00032505001   (113) EGEEKYCARSLESLINFSTSKLGKNIRALPNVVEGEIQEYKFGKGAKMLG
    VIT_04s0008g04120   (401) VGEVKYCARSLESLIDFSTSRLGKNIRVLSNEVEADIQEYKFGEGVKMVG
VIT_04s0008g04110_SV1    (62) VGEVKYCARSLESLIDFSTSRLGKNIRVLSNEVEADIQEYKFGEGVKMVG
                              451                                             500
    GSVIVP00032505001   (163) EKSVVEISGVPPAELSICCGILPYTSHDKDLHGSIGGC------------
    VIT_04s0008g04120   (451) EKSVVCHQLNYPYAVAFCHTLHMTKIYMVPSVGADGTGVEAVAVCHRDTS
VIT_04s0008g04110_SV1   (112) EKSVVCHQLNYPYAVAFCHTLHMTKIYMVPLVGADGTGVEAVAVCHRDTS
                              501                                             550
    GSVIVP00032505001   (201) --------------------------------------------------
    VIT_04s0008g04120   (501) TWDPKALVFQRLKVKPGTLPICHFLPNGHIVWVPKFNGVSSPSHSDFFLG
VIT_04s0008g04110_SV1   (162) TWDPKALVFQSLKVKPGTLPICHFLPNGHTVWVPK---------------
                              551                            583
    GSVIVP00032505001   (201) ---------------------------------
    VIT_04s0008g04120   (551) DWWWSAMLILYLPRLIGSRLCLTLPSRKLCGIS
VIT_04s0008g04110_SV1   (197) ---------------------------------
```

VvBURP15
DNA alignment

- Query: genomic (exon+introns) of GSVIVT00032506001 (8.4x)
- Sbjct: genomic 12x

```
Query  1        ATGCAATACGAGAGGGTCTATGGAAATCCTCAAAAAGCAGGCGGAAGTTCTGTAAGCGCG  60
                ||||||||||||||||||||||||||||||||||||||||||||||||||||||||||||
Sbjct  3464865  ATGCAATACGAGAGGGTCTATGGAAATCCTCAAAAAGCAGGCGGAAGTTCTGTAAGCGCG  3464924

Query  61       GGCAAAAAATAGTCCAAACATGCAACAAGGACACCAAGATGGTACCAGTACAGGTAATGTG  120
                ||||||||||||||||||||||||||||||||||||||||||||||||||||||||||||
Sbjct  3464925  GGCAAAAAATAGTCCAAACATGCAACAAGGACACCAAGATGGTACCAGTACAGGTAATGTG  3464984

Query  121      TTCTTGGAAAAAGACCTGCATCCTGGGACTAAAATGATGGTGCGCTTCACAAAAACCTCA  180
                ||||||||||||||||||||||||||||||||||||||||||||||||||||||||||||
Sbjct  3464985  TTCTTGGAAAAAGACCTGCATCCTGGGACTAAAATGATGGTGCGCTTCACAAAAACCTCA  3465044

Query  181      AGTGCAGCTCATTTCTTGCCCCAGCAAGTTGCTGAATCCATACCTTTTTTCATCAAACAA  240
                |||||||||||||||||||||||||||||||||||||||||||||||| |||||||||||
Sbjct  3465045  AGTGCAGCTCATTTCTTGCCCCAGCAAGTTGCTGAATCCATACCTTTT-TCATCAAACAA  3465103

Query  241      GCTACCAGAAATTTTGAATCAGTTCTCAGTGAAAGAAAACTCCGCAGAAGCCAAGATAAT  300
                ||||||||||||||||||||||||||||||||||||||||||||||||||||||||||||
Sbjct  3465104  GCTACCAGAAATTTTGAATCAGTTCTCAGTGAAAGAAAACTCCGCAGAAGCCAAGATAAT  3465163

Query  301      ACAGAAAACAATAGAAGAATGTGAGAAACCGGCTATAGAAGGAGAAGAGAAGTACTGTGC  360
                ||||||||||||||||||||||||||||||||||||||||||||||||||||||||||||
Sbjct  3465164  ACAGAAAACAATAGAAGAATGTGAGAAACCGGCTATAGAAGGAGAAGAGAAGTACTGTGC  3465223

Query  361      GAGATCGTTGGAGTCCCTAATCGATTTCAGCACTTCAAAGCTTGGAAAAAACATTCGAGC  420
                ||||||||||||||||||||||||||||||||||||||||||||||||||||||||||||
Sbjct  3465224  GAGATCGTTGGAGTCCCTAATCGATTTCAGCACTTCAAAGCTTGGAAAAAACATTCGAGC  3465283

Query  421      ACTCCCAAATGTGGTGGAAGGGGAAATCCAGGAATATAAATTTGGAGAGGGAGCGAAGAT  480
                ||||||||||||||||||||||||||||||||||||||||||||||||||||||||||||
Sbjct  3465284  ACTCCCAAATGTGGTGGAAGGGGAAATCCAGGAATATAAATTTGGAGAGGGAGCGAAGAT  3465343

Query  481      GCTTGGAGAGAAATCAGTGGTGTGCCACCAGCTGAACTATCCATATGCTGTGGCTCTTTG  540
                ||||||||||||||||||||||||||||||||||||||||||||||||||||||||||||
Sbjct  3465344  GCTTGGAGAGAAATCAGTGGTGTGCCACCAGCTGAACTATCCATATGCTGTGGCTCTTTG  3465403

Query  541      CCATGCATTTCACATGACAAAAATTTACAAGGTTCCATTGGTGGGAGCTGATGGAACCAG  600
                ||||||||||||||||||||||||||||||||||||||||||||||||||||||||||||
Sbjct  3465404  CCATGCATTTCACATGACAAAAATTTACAAGGTTCCATTGGTGGGAGCTGATGGAACCAG  3465463

Query  601      AGTTCAAGCTTTAGCAGTATGTCATGAGGACACTTCGATTTGGGACCCAAATGCTTTGGC  660
                ||||||||||||||||||||||||||||||||||||||||||||||||||||||||||||
Sbjct  3465464  AGTTCAAGCTTTAGCAGTATGTCATGAGGACACTTCGATTTGGGACCCAAATGCTTTGGC  3465523

Query  661      TTTTCAAGTGCTCAAAGTTAAGCCAGGAACTTGGCCCATCTGTCATTTCCTTCCCAATGG  720
                ||||||||||||||||||||||||||||||||||||||||||||||||||||||||||||
Sbjct  3465524  TTTTCAAGTGCTCAAAGTTAAGCCAGGAACTTGGCCCATCTGTCATTTCCTTCCCAATGG  3465583

Query  721      TCATTTTGTGTGGGTTCCAAACTAG  745
                |||||||||||||||||||||||||
Sbjct  3465584  TCATTTTGTGTGGGTTCCAAACTAG  3465608
```

- 12x VIT_04s0008g04130 is probably wrongly predicted to start at 554

VvBURP15
Protein alignment

```
                               1                                                50
GSVIVP00032506001     (1) MQYERVYGNPQKAGGSSVSAGKNSPNMQQGHQDGTSTGNVFLEKDLHPGT
VIT_04s0008g04130     (1) --------------------------------------------------
        Consensus     (1)
                               51                                              100
GSVIVP00032506001    (51) KMMVRFTKTSSAAHFLPQQVAESIPFFIKQATRNFESVLKCEKPAIEGEE
VIT_04s0008g04130     (1) --------------------------------------------------
        Consensus    (51)
                               101                                             150
GSVIVP00032506001   (101) KYCARSLESLIDFSTSKLGKNIRALPNVVEGEIQEYKFGEGAKMLGEKSV
VIT_04s0008g04130     (1) --------------------------------------------------
        Consensus   (101)
                               151                                             200
GSVIVP00032506001   (151) VCHQLNYPYAVALCHAFHMTKIYKVPLVGADGTRVQALAVCHEDTSIWDP
VIT_04s0008g04130     (1) ------------------MTKIYKVPLVGADGTRVQALAVCHEDTSIWDP
        Consensus   (151)                   MTKIYKVPLVGADGTRVQALAVCHEDTSIWDP
                               201                            231
GSVIVP00032506001   (201) NALAFQVLKVKPGTWPICHFLPNGHFVWVPN
VIT_04s0008g04130    (33) NALAFQVLKVKPGTWPICHFLPNGHFVWVPN
        Consensus   (201) NALAFQVLKVKPGTWPICHFLPNGHFVWVPN
```

VvBURP16
Protein alignment (arrows indicate alternative protein start sites)

```
                                    1                                                50
      GSVIVP00032509001     (1) --------------------------------------------------
      VIT_04s0008g04150     (1) --------------------------------------------------
  VIT_04s0008g04150_SV1     (1) --------------------------------------------------
  VIT_04s0008g04150_SV2     (1) MFSILEDQDKAQMKLFFTEQFQNIFLKILKKTICKFKWTELATCIFIFFI
              Consensus     (1)

                                    51                                              100
      GSVIVP00032509001     (1) --------------------------------------------------
      VIT_04s0008g04150     (1) ---MHLHLFYFKSSYIFANASLFLLATELWPLVVVESHASLRSELYWPLL
  VIT_04s0008g04150_SV1     (1) ------------------------------------MVFYLLPLLAFLSL
  VIT_04s0008g04150_SV2    (51) LSHRIYLLMLAYFFLQLNYGRLWWWKAMLLCDLSFTGPWYCQTLPNQSFL
              Consensus    (51)        L           A LF     L   L     FYL SLL F  L

                                    101                                             150
      GSVIVP00032509001     (1) --------------------------------------------------
      VIT_04s0008g04150    (48) TLTAGDSDLPSEIYWNSVLPNTPMPQAVKNSLRP-------------GVS
  VIT_04s0008g04150_SV1    (15) TLTAGDSDLPSEIYWNSVLPNTPMPQAVKNSLRPESTGVEIGKGTDIGVS
  VIT_04s0008g04150_SV2   (101) TLTAGDSDLPSEIYWNSVLPNTPMPQAVKNSLRP---------GTDIGVS
              Consensus   (101) TLTAGDSDLPSEIYWNSVLPNTPMPQAVKNSLRP         GTDIGVS

                                    151                                             200
      GSVIVP00032509001     (1) --------------------------------------------------
      VIT_04s0008g04150    (85) KGGVSVSTGHEEKPGYVGVTKAKKPIFFYRYAATEDQLHAHPSVALFFLE
  VIT_04s0008g04150_SV1    (65) KGGVSVSTGHEEKPGYVGVTKAKKPIFFYRYAATEDQLHAHPSVALFFLE
  VIT_04s0008g04150_SV2   (142) KGGVSVSTGHEEKPGYVGVTKAKKPIFFYRYAATEDQLHAHPSVALFFLE
              Consensus   (151) KGGVSVSTGHEEKPGYVGVTKAKKPIFFYRYAATEDQLHAHPSVALFFLE

                                    201                                             250
      GSVIVP00032509001     (1) --MRLGTKMNLDFMKNTNEATFLPHQVATSIPFSSDKLPEILDQLSVKPE
      VIT_04s0008g04150   (135) KDMRLGTKMNLDFMKNTNEATFLPHQVATSIPFSSDKLPEILDQLSVKPE
  VIT_04s0008g04150_SV1   (115) KDMRLGTKMNLDFMKNTNEATFLPHQVATSIPFSSDKLPEILDQLSVKPE
  VIT_04s0008g04150_SV2   (192) KDMRLGTKMNLDFMKNTNEATFLPHQVATSIPFSSDKLPEILDQLSVKPE
              Consensus   (201) KDMRLGTKMNLDFMKNTNEATFLPHQVATSIPFSSDKLPEILDQLSVKPE

                                    251                                             300
      GSVIVP00032509001    (49) SVEAETIKNTIIDCDRPGIKGEEKYCATSLESMIDFSTSKLGNKGVKAVS
      VIT_04s0008g04150   (185) SVEAETIKNTIIDCDRPGIKGEEKYCATSLESMIDFSTSKLGNKGVKAVS
  VIT_04s0008g04150_SV1   (165) SVEAETIKNTIIDCDRPGIKGEEKYCATSLESMIDFSTSKLGNKGVKAVS
  VIT_04s0008g04150_SV2   (242) SVEAETIKNTIIDCDRPGIKGEEKYCATSLESMIDFSTSKLGNKGVKAVS
              Consensus   (251) SVEAETIKNTIIDCDRPGIKGEEKYCATSLESMIDFSTSKLGNKGVKAVS

                                    301                                             350
      GSVIVP00032509001    (99) TEVENKSQTLYRIAAGVEKMGGDVSVVCHKMEYAYAVFYCHKIAATRAYM
      VIT_04s0008g04150   (235) TEVENKSQTLYRIAAGVEKMGGDVSVVCHKMEYAYAVFYCHKIAATRAYM
  VIT_04s0008g04150_SV1   (215) TEVENKSQTLYRIAAGVEKMGGDVSVVCHKMEYAYAVFYCHKIAATRAYM
  VIT_04s0008g04150_SV2   (292) TEVENKSQTLYRIAAGVEKMGGDVSVVCHKMEYAYAVFYCHKIAATRAYM
              Consensus   (301) TEVENKSQTLYRIAAGVEKMGGDVSVVCHKMEYAYAVFYCHKIAATRAYM

                                    351                                             400
      GSVIVP00032509001   (149) VPLVGRDGAKAKAVALCHTNTKEWNPKHLAFQLLKVKPGVPICHFLTQDQ
      VIT_04s0008g04150   (285) VPLVGRDGAKAKAVALCHTNTKEWNPKHLAFQLLKVKPGVPICHFLTQDQ
  VIT_04s0008g04150_SV1   (265) VPLVGRDGAKAKAVALCHTNTKEWNPKHLAFQLLKVKPGVPICHFLTQDQ
  VIT_04s0008g04150_SV2   (342) VPLVGRDGAKAKAVALCHTNTKEWNPKHLAFQLLKVKPGVPICHFLTQDQ
              Consensus   (351) VPLVGRDGAKAKAVALCHTNTKEWNPKHLAFQLLKVKPGVPICHFLTQDQ

                                    401
      GSVIVP00032509001   (199) IIWVVSK
      VIT_04s0008g04150   (335) IIWVVSK
  VIT_04s0008g04150_SV1   (315) IIWVVSK
  VIT_04s0008g04150_SV2   (392) IIWVVSK
              Consensus   (401) IIWVVSK
```

VvBURP17
Protein alignment

```
                                  1                                                50
    GSVIVP00032510001      (1)  MKLWQLVVVESRASLRSELYWPLVLPNTPKPKFCGFSGGLLQLTLTAGDA
    GSVIVP00032511001      (1)  --------------------------------------------------
      VIT_04s0008g04160    (1)  MKLWQLVVVESRASLRSELYWPLVLPNTPKPKFCGFSGGLLQLTLTAGDA
  VIT_04s0008g04160_SV1    (1)  MKLWQLVVVESRASLRSELYWPLVLPNTPKPKFCGFSGGLLQLTLTAGDA
              Consensus    (1)  MKLWQLVVVESRASLRSELYWPLVLPNTPKPKFCGFSGGLLQLTLTAGDA

                                  51                                              100
    GSVIVP00032510001     (51)  DLPSEVYWSSVLPNTPMPQAVKNSLRPGWL--------------------
    GSVIVP00032511001      (1)  ---------------------MVIEYADLLEDQSTPVEIGKGTSMGFSKE
      VIT_04s0008g04160   (51)  DLPSEVYWSSVLPNTPMPQAVKNSLRPDLLEDQSTPVEIGKGTSMGFSKE
  VIT_04s0008g04160_SV1   (51)  DLPSEVYWSSVLPNTPMPQAVKNSLRPDLLEDQSTPVEIGKGTSMGFSKE
              Consensus   (51)  DLPSEVYWSSVLPNTPMPQAVKNSLRPDLLEDQSTPVEIGKGTSMGFSKE

                                  101                                             150
    GSVIVP00032510001     (81)  --------------------------------------------------
    GSVIVP00032511001     (30)  GGAMNMYAGVKPAKAAMPFSYHYAATKDQLHAYPNVAIFFLEKDMHPGMK
      VIT_04s0008g04160  (101)  GGAMNMYAGVKPAKAAMPFSYHYAATKDQLHAYPNVAIFFLEKDMHPGMK
  VIT_04s0008g04160_SV1  (101)  GGAMNMYAGVKPAKAAMPFSYHYAATKDQLHAYPNVAIFFLEKDMHPGMK
              Consensus  (101)  GGAMNMYAGVKPAKAAMPFSYHYAATKDQLHAYPNVAIFFLEKDMHPGMK

                                  151                                             200
    GSVIVP00032510001     (81)  --------------------------------------------------
    GSVIVP00032511001     (80)  LTLHFTKTTNATFLPHQVANSLPFSSDKLAEILDQLSIKPESVEAETIKN
      VIT_04s0008g04160  (151)  LTLHFTKTTNATFLPHQVANSLPFSSDKLAEILDQLSIKPESVEAETIKN
  VIT_04s0008g04160_SV1  (151)  LTLHFTKTTNATFLPHQVANSLPFSSDKLAEILDQLSIKPESVEAETIKN
              Consensus  (151)  LTLHFTKTTNATFLPHQVANSLPFSSDKLAEILDQLSIKPESVEAETIKN

                                  201                                             250
    GSVIVP00032510001     (81)  --------------------------------------------------
    GSVIVP00032511001    (130)  TIEECEDPGIKGEEKYCATSLESMIDFSTSKLGNKGVKAVSTEAENKSQM
      VIT_04s0008g04160  (201)  TIEECEDPGIKGEEKYCATSLESMIDFSTSKLGNKGVKAVSTEAENKSQM
  VIT_04s0008g04160_SV1  (201)  TIEECEDPGIKGEEKYCATSLESMIDFSTSKLGNKGVKAVSTEAENKSQM
              Consensus  (201)  TIEECEDPGIKGEEKYCATSLESMIDFSTSKLGNKGVKAVSTEAENKSQM

                                  251                                             300
    GSVIVP00032510001     (81)  --------------------------------------------------
    GSVIVP00032511001    (180)  KYRIAAGLEKMGGDFSVVCHKMNYPYAVFYCHKIQATRAYMVPLVGRDGT
      VIT_04s0008g04160  (251)  KYRIAAGLEKMGGDFSVVCHKMNYPYAVFYCHKIQATRAYMVPLVGRDGT
  VIT_04s0008g04160_SV1  (251)  KYRIAAGLEKMGGDFSVVCHKMNYPYAVFYCHKIQATRAYMVPLVGRDGT
              Consensus  (251)  KYRIAAGLEKMGGDFSVVCHKMNYPYAVFYCHKIQATRAYMVPLVGRDGT

                                  301                                           348
    GSVIVP00032510001     (81)  ------------------------------------------------
    GSVIVP00032511001    (230)  KAKAVAVCHANTMEWNPNHLAFQLLKVKPGTAPICHFLPEDHVVWVAK
      VIT_04s0008g04160  (301)  KAKAVAVCHANTMEWNPNHLAFQLLKVKPGTAPICHFLPEDHVVWVAK
  VIT_04s0008g04160_SV1  (301)  KAKAVAVCHANTMEWNPNHLAFQLLKVKPGTAPICHFLPEDHVVWVAK
              Consensus  (301)  KAKAVAVCHANTMEWNPNHLAFQLLKVKPGTAPICHFLPEDHVVWVAK
```

VvBURP19

DNA alignment. Black boxes: Translation start and end sites.

```
                                1                                                50
    GSVIVT00016109001     (1) --------------------------------------------------
     VIT_17s0000g08160     (1) --------------------------------------------------
VIT_17s0000g08160_SV1     (1) CTTCGATTTTGGTGTCCTAGTGGAACCCGTTCAATGGTGCTGGCTGCTCT
            Consensus     (1)
                                51                                              100
    GSVIVT00016109001     (1) --------------------------------------------------
    VIT_17s0000g08160     (1) --------------------------------------------------
VIT_17s0000g08160_SV1    (51) GAATCTTGTGTTCCGCACATGATAATGTGGTTGTGTTTACAAGAATCACT
            Consensus    (51)
                                101                                             150
    GSVIVT00016109001     (1) --------------------------------------------------
    VIT_17s0000g08160     (1) --------------------------------------------------
VIT_17s0000g08160_SV1   (101) TGCTCCCCATTAACAACTTACTCTATGGTCTTGATTCCCATGATCATATG
            Consensus   (101)
                                151                                             200
    GSVIVT00016109001     (1) --------------------------------------------------
    VIT_17s0000g08160     (1) --------------------------------------------------
VIT_17s0000g08160_SV1   (151) AATTAAAATCATCATTCACTTCATTCCTATTTGAGTACGCAAGATCCACT
            Consensus   (151)
                                201                                             250
    GSVIVT00016109001     (1) --------------------------------------------------
    VIT_17s0000g08160     (1) --------------------------------------------------
VIT_17s0000g08160_SV1   (201) ATGAGTGGAAAAAATAAAAAGATTAAGAGCAGAATAAAGGAGAAAGAGGA
            Consensus   (201)
                                251                                             300
    GSVIVT00016109001     (1) --------------------------------------------------
    VIT_17s0000g08160     (1) --------------------------------------------------
VIT_17s0000g08160_SV1   (251) GTCAAGTGGAAGGTATCGTCAAATGATGGCTACGCCTTTGATTTTGTGAA
            Consensus   (251)
                                301                                             350
    GSVIVT00016109001     (1) --------------------------------------------------
    VIT_17s0000g08160     (1) --------------------------------------------------
VIT_17s0000g08160_SV1   (301) TCTTTTTGGCCAATTCACAACACCAGAGAGGAGACAAAGAGCCAACTTTG
            Consensus   (301)
                                351                                             400
    GSVIVT00016109001     (1) --------------------------------------------------
    VIT_17s0000g08160     (1) --------------------------------------------------
VIT_17s0000g08160_SV1   (351) TAAAGCAAAATCCTTGTAAAACAAAAACGAAATACTATTCTCAACACAGA
            Consensus   (351)
                                401                                             450
    GSVIVT00016109001     (1) --------------------------------------------------
    VIT_17s0000g08160     (1) --------------------------------------------------
VIT_17s0000g08160_SV1   (401) CTCAGAGGGGGTGGTTCTTTCTCTCTTTATTTATATTACCTGCTTCCCTT
            Consensus   (401)
                                451                                             500
    GSVIVT00016109001     (1) ---------------------------CAGACATGGAAACAATGCGCACA
    VIT_17s0000g08160     (1) ---------------------------CAGACATGGAAACAATGCGCACA
VIT_17s0000g08160_SV1   (451) CTTCCAGATCTCTCTCTTTCAGATTCTCAGACATGGAAACAATGCGCACA
            Consensus   (451)                            CAGACATGGAAACAATGCGCACA
                                501                                             550
    GSVIVT00016109001    (24) CCGCCATTGCTCACAACCATTCTCTTCGTATTCTTCTTGTACTTGTCATT
    VIT_17s0000g08160    (24) CCGCCATTGCTCACAACCATTCTCTTCGTATTCTTCTTGTACTTGTCATT
VIT_17s0000g08160_SV1   (501) CCGCCATTGCTCACAACCATTCTCTACGTATTCTTCTTGTACTTGTCATT
            Consensus   (501) CCGCCATTGCTCACAACCATTCTCTTCGTATTCTTCTTGTACTTGTCATT
                                551                                             600
    GSVIVT00016109001    (74) CACATCTTTCAATGTACTGAATCGAAACCTTCATCTTGTTTTTTTTGCTT
    VIT_17s0000g08160    (74) CACATCTTTCAAT-------------------------------------
VIT_17s0000g08160_SV1   (551) CACATCTTTCAAT-------------------------------------
            Consensus   (551) CACATCTTTCAAT
                                601                                             650
    GSVIVT00016109001   (124) TCTATCTGTTTTGTCTTACTTCTGATCTTCTTGCTTGGATTCTGTGAATG
    VIT_17s0000g08160    (87) --------------------------------------------------
VIT_17s0000g08160_SV1   (564) --------------------------------------------------
            Consensus   (601)
```

```
                                   651                                               700
    GSVIVT00016109001   (174)  CAGGTATGTTTCGCTGAAGGAAAGTCATCCACCGGTGGCAACCCCTTTAC
    VIT_17s0000g08160    (87)  ---GTATGTTTCGCTGAAGGAAAGTCATCCACCGGTGGCAACCCCTTTAC
VIT_17s0000g08160_SV1   (564)  ---GTATGTTTCGCTGAAGGAAAGTCATCCACCGGTGGCAACCCCTTTAC
            Consensus   (651)     GTATGTTTCGCTGAAGGAAAGTCATCCACCGGTGGCAACCCCTTTAC
                               701                                               750
    GSVIVT00016109001   (224)  GGCGAAAGCTTCCCTGATTCGGTATTGGAACAAACAGATATCGAACAAGC
    VIT_17s0000g08160   (134)  GGCGAAAGCTTCCCTGATTCGGTATTGGAACAAACAGATATCGAACAAGC
VIT_17s0000g08160_SV1   (611)  GGCGAAAGCTTCCCTGATTCGGTATTGGAACAAACAGATATCGAACAAGC
            Consensus   (701)  GGCGAAAGCTTCCCTGATTCGGTATTGGAACAAACAGATATCGAACAAGC
                               751                                               800
    GSVIVT00016109001   (274)  TCCCCAAACCATCTTTTCTTCTCTCAAAAGCTTCCCCACTCAATGCCGTC
    VIT_17s0000g08160   (184)  TCCCCAAACCATCTTTTCTTCTCTCAAAAGCTTCCCCACTCAATGCCGTC
VIT_17s0000g08160_SV1   (661)  TCCCCAAACCATCTTTTCTTCTCTCAAAAGCTTCCCCACTCAATGCCGTC
            Consensus   (751)  TCCCCAAACCATCTTTTCTTCTCTCAAAAGCTTCCCCACTCAATGCCGTC
                               801                                               850
    GSVIVT00016109001   (324)  GACTCGGCCGTTCTCACCAAACTCGCCACCCAAAACGCTCTGTCTTCTCA
    VIT_17s0000g08160   (234)  GACTCGGCCGTTCTCACCAAACTCGCCACCCAAAACGCTCTGTCTTCTCA
VIT_17s0000g08160_SV1   (711)  GACTCGGCCGTTCTCACCAAACTCGCCACCCAAAACGCTCTGTCTTCTCA
            Consensus   (801)  GACTCGGCCGTTCTCACCAAACTCGCCACCCAAAACGCTCTGTCTTCTCA
                               851                                               900
    GSVIVT00016109001   (374)  TCTATCATCCTTCTGCTCCTCTGCCAACTTGTTCTGTGTCTTCGACTCAT
    VIT_17s0000g08160   (284)  TCTATCATCCTTCTGCTCCTCTGCCAACTTGTTCTGTGTCTTCGACTCAT
VIT_17s0000g08160_SV1   (761)  TCTATCATCCTTCTGCTCATCTGCCAACTTGTTCTGTGTCTTCGACTCAG
            Consensus   (851)  TCTATCATCCTTCTGCTCCTCTGCCAACTTGTTCTGTGTCTTCGACTCAT
                               901                                               950
    GSVIVT00016109001   (424)  CGCCCACTTCGGAAAAACAAGTCAAGGACGCTAACTTCGCTTTTTACTCC
    VIT_17s0000g08160   (334)  CGCCCACTTCGGAAAAACAAGTCAAGGACGCTAACTTCGCTTTTTACTCC
VIT_17s0000g08160_SV1   (811)  CGCCCACTTCGGAAAAACAAGTCAAGGACGCTAACTTCGCTTTTTACTCC
            Consensus   (901)  CGCCCACTTCGGAAAAACAAGTCAAGGACGCTAACTTCGCTTTTTACTCC
                               951                                              1000
    GSVIVT00016109001   (474)  AATAGAGGATTCGCCAACTATGGCGACTCGCGAATCGGTGGTGTTGACTC
    VIT_17s0000g08160   (384)  AATAGAGGATTCGCCAACTATGGCGACTCGCGAATCGGTGGTGTTGACTC
VIT_17s0000g08160_SV1   (861)  AATAGAGGATTCGCCAACTATGGCGACTCGCGAATCGGTGGTGTTGACTC
            Consensus   (951)  AATAGAGGATTCGCCAACTATGGCGACTCGCGAATCGGTGGTGTTGACTC
                               1001                                             1050
    GSVIVT00016109001   (524)  GTTCAAGAATTACTCCGACGGGTTGAACATGCCCAGCGGCTCGTTCCGGC
    VIT_17s0000g08160   (434)  GTTCAAGAATTACTCCGACGGGTTGAACATGCCCAGCGGCTCGTTCCGGC
VIT_17s0000g08160_SV1   (911)  GTTCAAGAATTACTCCGACGGGTTGAACATGCCCAGCGGCTCGTTCCGGC
            Consensus  (1001)  GTTCAAGAATTACTCCGACGGGTTGAACATGCCCAGCGGCTCGTTCCGGC
                               1051                                             1100
    GSVIVT00016109001   (574)  GGTACAGCGGGGACTCCACTGACCACCACGAAGAGTTCACGAGCTACGCT
    VIT_17s0000g08160   (484)  GGTACAGCGGGGACTCCACTGACCACCACGAAGAGTTCACGAGCTACGCT
VIT_17s0000g08160_SV1   (961)  GGTACAGCGGGGACTCCACTGACCACCAAGAAGAGTTCACGAGCTACGCT
            Consensus  (1051)  GGTACAGCGGGGACTCCACTGACCACCACGAAGAGTTCACGAGCTACGCT
                               1101                                             1150
    GSVIVT00016109001   (624)  CGAGACGGGAACGTCGCCACCGGGAGCTTCGCCGGCTACGGCTCAGGCGC
    VIT_17s0000g08160   (534)  CGAGACGGGAACGTCGCCACCGGGAGCTTCGCCGGCTACGGCTCAGGCGC
VIT_17s0000g08160_SV1  (1011)  CGCGACGGGAACGTCGCCACCGGGACCTTCGCCGGCTACGG---------
            Consensus  (1101)  CGAGACGGGAACGTCGCCACCGGGAGCTTCGCCGGCTACGGCTCAGGCGC
                               1151                                             1200
    GSVIVT00016109001   (674)  CACCGGCGGCTCCGGTGAGTTCACGAACTACGACCCACTGGTCAACGTCC
    VIT_17s0000g08160   (584)  CACCGGCGGCTCCGGTGAGTTCACGAACTACGACCCACTGGTCAACGTCC
VIT_17s0000g08160_SV1  (1052)  ----------------GAGTTCACGAACTACGACCCACTGGTCAACGTCC
            Consensus  (1151)  CACCGGCGGCTCCGGTGAGTTCACGAACTACGACCCACTGGTCAACGTCC
                               1201                                             1250
    GSVIVT00016109001   (724)  CCCATCTCGAATTCACCACGTACGACCCCAACGGCAACAACCACAAACTC
    VIT_17s0000g08160   (634)  CCCATCTCGAATTCACCACGTACGACCCCAACGGCAACAACCACAAACTC
VIT_17s0000g08160_SV1  (1086)  CCCATCTCGAATTCACCACGTACGACCCCAACGGCAACAACCACAAACTC
            Consensus  (1201)  CCCATCTCGAATTCACCACGTACGACCCCAACGGCAACAACCACAAACTC
                               1251                                             1300
    GSVIVT00016109001   (774)  ACGTTCGCGGCCTACACCGATGACACCAACTCCGGCACCGAGTCCTTCAC
    VIT_17s0000g08160   (684)  ACGTTCGCGGCCTACACCGATGACACCAACTCCGGCACCGAGTCCTTCAC
VIT_17s0000g08160_SV1  (1136)  ACGTTCGCGGCCTACACCGATGAGACCAACTCCGGCACCGAATCCTTCAC
            Consensus  (1251)  ACGTTCGCGGCCTACACCGATGACACCAACTCCGGCACCGAGTCCTTCAC
                               1301                                             1350
    GSVIVT00016109001   (824)  CAGCTACGGCAAGAACGGCAAGAACGTCCCGGCTGAGTTCACCAGCTACG
    VIT_17s0000g08160   (734)  CAGCTACGGCAAGAACGGCAAGAACGTCCCGGCTGAGTTCACCAGCTACG
VIT_17s0000g08160_SV1  (1186)  CAGCTACGGCAAGAACGGCAAGAACGTCCCGGCTGAGTTCACCAGCTACG
            Consensus  (1301)  CAGCTACGGCAAGAACGGCAAGAACGTCCCGGCTGAGTTCACCAGCTACG
```

```
                                      1351                                              1400
      GSVIVT00016109001     (874)  GCGACAACTCCAACATCATCGGCTCCACTTTCACAGCTTACGGCCAATTA
      VIT_17s0000g08160     (784)  GCGACAACTCCAACATCATCGGCTCCACTTTCACAGCTTACGGCCAATTA
  VIT_17s0000g08160_SV1    (1236)  GCGACAACTCCAACATCATCGGCTCCACTTTCACAGCTTACGGCCAATTA
              Consensus    (1351)  GCGACAACTCCAACATCATCGGCTCCACTTTCACAGCTTACGGCCAATTA

                                      1401                                              1450
      GSVIVT00016109001     (924)  GCCAATAGCCAAAACGACTCGTTCAAAGCTTACGGCCACTCCTCCAACAA
      VIT_17s0000g08160     (834)  GCCAATAGCCAAAACGACTCGTTCAAAGCTTACGGCCACTCCTCCAACAA
  VIT_17s0000g08160_SV1    (1286)  GCCAATGGCCAAAACGACTCGTTCAAAGCTTACGGCCACTCCTCCAACAA
              Consensus    (1401)  GCCAATAGCCAAAACGACTCGTTCAAAGCTTACGGCCACTCCTCCAACAA

                                      1451                                              1500
      GSVIVT00016109001     (974)  CCCACATAACAATTTCAAGAGCTACAGCCTTGGAGGCAATGCCGCAACGG
      VIT_17s0000g08160     (884)  CCCACATAACAATTTCAAGAGCTACAGCCTTGGAGGCAATGCCGCAACGG
  VIT_17s0000g08160_SV1    (1336)  CCCACATAACAATTTCAAGAGCTACAGCCTTGGAGGCAATGCCGCAACGG
              Consensus    (1451)  CCCACATAACAATTTCAAGAGCTACAGCCTTGGAGGCAATGCCGCAACGG

                                      1501                                              1550
      GSVIVT00016109001    (1024)  ATACCTTCTCAAATTACAGAGATGGAGCCAATGTGGGTGACGATTCATTT
      VIT_17s0000g08160     (934)  ATACCTTCTCAAATTACAGAGATGGAGCCAATGTGGGTGACGATTCATTT
  VIT_17s0000g08160_SV1    (1386)  ATACCTTCTCAAATTACAGAGATGGAGCCAATGTGGGTGACGATTCATTT
              Consensus    (1501)  ATACCTTCTCAAATTACAGAGATGGAGCCAATGTGGGTGACGATTCATTT

                                      1551                                              1600
      GSVIVT00016109001    (1074)  CAATCTTATGCCAGGAGTTCGAACTCTGCAGAAGTGAATTTCGCGAATTA
      VIT_17s0000g08160     (984)  CAATCTTATGCCAGGAGTTCGAACTCTGCAGAAGTGAATTTCGCGAATTA
  VIT_17s0000g08160_SV1    (1436)  CAATCTTATGCCAGGAGTTCGAACTCTGCAGAAGTGAATTTCGCGAATTA
              Consensus    (1551)  CAATCTTATGCCAGGAGTTCGAACTCTGCAGAAGTGAATTTCGCGAATTA

                                      1601                                              1650
      GSVIVT00016109001    (1124)  TGGGAGGACATTCAACGTAGGAAACGACACATTCAAAGAATACGGAAAAG
      VIT_17s0000g08160    (1034)  TGGGAGGACATTCAACGTAGGAAACGACACATTCAAAGAATACGGAAAAG
  VIT_17s0000g08160_SV1    (1486)  TGGGAGGACATTCAACGTAGGAAACGACACATTCAAAGAATACGGAAAAG
              Consensus    (1601)  TGGGAGGACATTCAACGTAGGAAACGACACATTCAAAGAATACGGAAAAG

                                      1651                                              1700
      GSVIVT00016109001    (1174)  GGTCGACGGATTCGTCGGTTGGGTTCAAAATCTACGGTTTGAATTACACA
      VIT_17s0000g08160    (1084)  GGTCGACGGATTCGTCGGTTGGGTTCAAAATCTACGGTTTGAATTACACA
  VIT_17s0000g08160_SV1    (1536)  GGTCGACGGATTCGTCGGTTGGGTTCAAAATCTACGGTTTGAATTACACA
              Consensus    (1651)  GGTCGACGGATTCGTCGGTTGGGTTCAAAATCTACGGTTTGAATTACACA

                                      1701                                              1750
      GSVIVT00016109001    (1224)  TTCAAAGACTATGAAAGAAAGGGTGTAACCTTCTCTCAATACAGCCGTGC
      VIT_17s0000g08160    (1134)  TTCAAAGACTATGAAAGAAAGGGTGTAACCTTCTCTCAATACAGCCGTGC
  VIT_17s0000g08160_SV1    (1586)  TTCAAAGACTATGAAAGAAAGGGTGTAACCTTCTCTCAATACAGCCGTGC
              Consensus    (1701)  TTCAAAGACTATGAAAGAAAGGGTGTAACCTTCTCTCAATACAGCCGTGC

                                      1751                                              1800
      GSVIVT00016109001    (1274)  AAGTAGCAACACTACCGCTACCGCGACTACCGCGAGTGGCATTTCCGTAA
      VIT_17s0000g08160    (1184)  AAGTAGCAACACTACCGCTACCGCGACTACCGCGAGTGGCATTTCCGTAA
  VIT_17s0000g08160_SV1    (1636)  AAGTAGCAACACTACTGCTACTGCGACTACCGCGAGTGGCATCTCCGTAA
              Consensus    (1751)  AAGTAGCAACACTACCGCTACCGCGACTACCGCGAGTGGCATTTCCGTAA

                                      1801                                              1850
      GSVIVT00016109001    (1324)  ATAGATGGGTCGAGCCGGGCAAATTCTTCCGCGAGTCCATGTTAAAGGAG
      VIT_17s0000g08160    (1234)  ATAGATGGGTCGAGCCGGGCAAATTCTTCCGCGAGTCCATGTTAAAGGAG
  VIT_17s0000g08160_SV1    (1686)  ATAGATGGGTCGAGCCGGGCAAATTCTTCCGCGAGTCCATGTTAAAGGAG
              Consensus    (1801)  ATAGATGGGTCGAGCCGGGCAAATTCTTCCGCGAGTCCATGTTAAAGGAG

                                      1851                                              1900
      GSVIVT00016109001    (1374)  GGGAATGTTATGGTGATGCCAGACATACGCGACAGAATGCCCGAAAGGTC
      VIT_17s0000g08160    (1284)  GGGAATGTTATGGTGATGCCAGACATACGCGACAGAATGCCCGAAAGGTC
  VIT_17s0000g08160_SV1    (1736)  GGGAATGTTATGGTGATGCCAGACATACGCGACAGAATGCCCGAAAGGTC
              Consensus    (1851)  GGGAATGTTATGGTGATGCCAGACATACGCGACAGAATGCCCGAAAGGTC

                                      1901                                              1950
      GSVIVT00016109001    (1424)  GTTTTTGCCCCGCGTCATCGCGTCCAAATTACCATTTTCCTCCTCTCGTC
      VIT_17s0000g08160    (1334)  GTTTTTGCCCCGCGTCATCGCGTCCAAATTACCATTTTCCTCCTCTCGTC
  VIT_17s0000g08160_SV1    (1786)  GTTTTTGCCCCGCGTCATCGCGTCCAAATTACCATTTTCCTCCTCTCGTC
              Consensus    (1901)  GTTTTTGCCCCGCGTCATCGCGTCCAAATTACCATTTTCCTCCTCTCGTC
```

```
                                    1951                                              2000
       GSVIVT00016109001   (1474)   TCCAGGAGCTCAAGGACATCTTCCACGCGCGGGACAACTCCACGATGGAG
       VIT_17s0000g08160   (1384)   TCCAGGAGCTCAAGGACATCTTCCACGCGCGGGACAACTCCACGATGGAG
   VIT_17s0000g08160_SV1   (1836)   TCCAGGAGCTCAAGGACATGTTCCACGCGCGGGACAACTCCACGATGGAG
               Consensus   (1951)   TCCAGGAGCTCAAGGACATCTTCCACGCGCGGGACAACTCCACGATGGAG
                                    2001                                              2050
       GSVIVT00016109001   (1524)   CACGTGATTGCGAACGCGCTGGAAGAGTGCGAGAGAGCTCCGAGTCGCGG
       VIT_17s0000g08160   (1434)   CACGTGATTGCGAACGCGCTGGAAGAGTGCGAGAGAGCTCCGAGTCGCGG
   VIT_17s0000g08160_SV1   (1886)   CACGTGCTTGCGAACGCGCTGGAAGAGTGCGAGAGAGCTCCGAGTCGCGG
               Consensus   (2001)   CACGTGATTGCGAACGCGCTGGAAGAGTGCGAGAGAGCTCCGAGTCGCGG
                                    2051                                              2100
       GSVIVT00016109001   (1574)   CGAGACCAAGCGGTGCGTGGGTTCGGTCGAGGACATGATAGACTTCGCCG
       VIT_17s0000g08160   (1484)   CGAGACCAAGCGGTGCGTGGGTTCGGTCGAGGACATGATAGACTTCGCCG
   VIT_17s0000g08160_SV1   (1936)   CGAGACCAAGCGGTGCGTGGGTTCGGTCGAGGACATGATAGACTTCGCCG
               Consensus   (2051)   CGAGACCAAGCGGTGCGTGGGTTCGGTCGAGGACATGATAGACTTCGCCG
                                    2101                                              2150
       GSVIVT00016109001   (1624)   TCTCGGTCCTGGGGCACGACGTGGTGGTGAGAACCACGGAGACGACGCGT
       VIT_17s0000g08160   (1534)   TCTCGGTCCTGGGGCACGACGTGGTGGTGAGAACCACGGAGACGACGCGT
   VIT_17s0000g08160_SV1   (1986)   TCTCGGTCCTGGGGCACGACGTGGTGGTGAGAACCACGGAGACGACGCGT
               Consensus   (2101)   TCTCGGTCCTGGGGCACGACGTGGTGGTGAGAACCACGGAGACGACGCGT
                                    2151                                              2200
       GSVIVT00016109001   (1674)   GGGTCAAAGCAGAGGGTGATGGTGGGGGAAGTCAGAGGGATCAACGGCGG
       VIT_17s0000g08160   (1584)   GGGTCAAAGCAGAGGGTGATGGTGGGGGAAGTCAGAGGGATCAACGGCGG
   VIT_17s0000g08160_SV1   (2036)   GGGTCAAAGCAGAGGGTGATGGTGGGGGAAGTCAGAGGGATCAACGGCGG
               Consensus   (2151)   GGGTCAAAGCAGAGGGTGATGGTGGGGGAAGTCAGAGGGATCAACGGCGG
                                    2201                                              2250
       GSVIVT00016109001   (1724)   AAAAGTGACTAGATCAGTATCATGCCACCAAAGCCTGTACCCCTACTTAC
       VIT_17s0000g08160   (1634)   AAAAGTGACTAGATCAGTATCATGCCACCAAAGCCTGTACCCCTACTTAC
   VIT_17s0000g08160_SV1   (2086)   AAAAGTGACTAAATCAGTATCATGCCACCAAAGCCTGTACCCCTACTTAC
               Consensus   (2201)   AAAAGTGACTAGATCAGTATCATGCCACCAAAGCCTGTACCCCTACTTAC
                                    2251                                              2300
       GSVIVT00016109001   (1774)   TGTATTATTGCCACTCAGTTCCCAAGGTTAGGGTTTACGAGGTGGACATT
       VIT_17s0000g08160   (1684)   TGTATTATTGCCACTCAGTTCCCAAGGTTAGGGTTTACGAGGTGGACATT
   VIT_17s0000g08160_SV1   (2136)   TGTATTATTGCCACTCAGTTCCCAAGGTTAGGGTTTACGAGGTGGACATT
               Consensus   (2251)   TGTATTATTGCCACTCAGTTCCCAAGGTTAGGGTTTACGAGGTGGACATT
                                    2301                                              2350
       GSVIVT00016109001   (1824)   CTTGACGTGGAGCGCAAAGAGAAGATGAATAAAGGGGTTGCCATCTGTCA
       VIT_17s0000g08160   (1734)   CTTGACGTGGAGCGCAAAGAGAAGATGAATAAAGGGGTTGCCATCTGTCA
   VIT_17s0000g08160_SV1   (2186)   CTTGACGTGGAGCGCAAAGAGAAGATGAATAAAGGGATTGCCATCTGTCA
               Consensus   (2301)   CTTGACGTGGAGCGCAAAGAGAAGATGAATAAAGGGGTTGCCATCTGTCA
                                    2351                                              2400
       GSVIVT00016109001   (1874)   TATTGACACGTCAGCATGGAGTCAGAGCCATGGCGCTTTCGTGGCGCTGG
       VIT_17s0000g08160   (1784)   TATTGACACGTCAGCATGGAGTCAGAGCCATGGCGCTTTCGTGGCGCTGG
   VIT_17s0000g08160_SV1   (2236)   TATTGACACGTCAGCATGGAGTCAGAGCCATGGCGCTTTCGTGGCGCTGG
               Consensus   (2351)   TATTGACACGTCAGCATGGAGTCAGAGCCATGGCGCTTTCGTGGCGCTGG
                                    2401                                              2450
       GSVIVT00016109001   (1924)   GGTCCAGCCCTGGGCTCATAGAGGTGTGCCATTGGATCTTTGAGAATGAC
       VIT_17s0000g08160   (1834)   GGTCCAGCCCTGGGCTCATAGAGGTGTGCCATTGGATCTTTGAGAATGAC
   VIT_17s0000g08160_SV1   (2286)   GGTCCAGCCCTGGGCTCATAGAGGTGTGCCATTGGATCTTTGAGAATGAC
               Consensus   (2401)   GGTCCAGCCCTGGGCTCATAGAGGTGTGCCATTGGATCTTTGAGAATGAC
                                    2451                                              2500
       GSVIVT00016109001   (1974)   ATGACATGGACGACTTCTGATTGA--------------------------
       VIT_17s0000g08160   (1884)   ATGACATGGACGACTTCTGATTGATGATAAAT------------------
   VIT_17s0000g08160_SV1   (2336)   ATGACATGGACGACTTCTGATTGATGATAAATTATTTTTCATTTTTTATT
               Consensus   (2451)   ATGACATGGACGACTTCTGATTGATGATAAAT
                                    2501                                              2550
       GSVIVT00016109001   (1998)   --------------------------------------------------
       VIT_17s0000g08160   (1916)   --------------------------------------------------
   VIT_17s0000g08160_SV1   (2386)   TTATTTTTTCACTTTTTCTCCATTTTTACTTTTGTTGGTTTGGAAGTTGA
               Consensus   (2501)
                                    2551                2572
       GSVIVT00016109001   (1998)   ----------------------
       VIT_17s0000g08160   (1916)   ----------------------
   VIT_17s0000g08160_SV1   (2436)   ACGGGGCTGCTTTCCCTTCCCC
               Consensus   (2551)
```

VvBURP19
DNA alignment

```
                                        1                                                50
        GSVIVP00016109001      (1)     -------------------------MQVCFAEGKSSTGGNPFTAKASLIR
        VIT_17s0000g08160      (1)     METMRTPPLLTTILFVFFLYLSFTSFNVCFAEGKSSTGGNPFTAKASLIR
    VIT_17s0000g08160_SV1      (1)     METMRTPPLLTTILYVFFLYLSFTSFNVCFAEGKSSTGGNPFTAKASLIR
                Consensus      (1)     METMRTPPLLTTILFVFFLYLSFTSFNVCFAEGKSSTGGNPFTAKASLIR
                                        51                                              100
        GSVIVP00016109001     (26)     YWNKQISNKLPKPSFLLSKASPLNAVDSAVLTKLATQNALSSHLSSFCSS
        VIT_17s0000g08160     (51)     YWNKQISNKLPKPSFLLSKASPLNAVDSAVLTKLATQNALSSHLSSFCSS
    VIT_17s0000g08160_SV1     (51)     YWNKQISNKLPKPSFLLSKASPLNAVDSAVLTKLATQNALSSHLSSFCSS
                Consensus     (51)     YWNKQISNKLPKPSFLLSKASPLNAVDSAVLTKLATQNALSSHLSSFCSS
                                        101                                             150
        GSVIVP00016109001     (76)     ANLFCVFDSSPTSEKQVKDANFAFYSNRGFANYGDSRIGGVDSFKNYSDG
        VIT_17s0000g08160    (101)     ANLFCVFDSSPTSEKQVKDANFAFYSNRGFANYGDSRIGGVDSFKNYSDG
    VIT_17s0000g08160_SV1    (101)     ANLFCVFDSAPTSEKQVKDANFAFYSNRGFANYGDSRIGGVDSFKNYSDG
                Consensus    (101)     ANLFCVFDSSPTSEKQVKDANFAFYSNRGFANYGDSRIGGVDSFKNYSDG
                                        151                                             200
        GSVIVP00016109001    (126)     LNMPSGSFRRYSGDSTDHHEEFTSYARDGNVATGSFAGYGSGATGGSGEF
        VIT_17s0000g08160    (151)     LNMPSGSFRRYSGDSTDHHEEFTSYARDGNVATGSFAGYGSGATGGSGEF
    VIT_17s0000g08160_SV1    (151)     LNMPSGSFRRYSGDSTDHQEEFTSYARDGNVATGTFAGYGSSRTT---TH
                Consensus    (151)     LNMPSGSFRRYSGDSTDHHEEFTSYARDGNVATGSFAGYGSGATGGSGEF
                                        201                                             250
        GSVIVP00016109001    (176)     TNYDPLVNVPHLEFTTYDPNGNNHKLTFAAYTDDTNSGTESFTSYGKNGK
        VIT_17s0000g08160    (201)     TNYDPLVNVPHLEFTTYDPNGNNHKLTFAAYTDDTNSGTESFTSYGKNGK
    VIT_17s0000g08160_SV1    (198)     WSTSPISNSPRTTPTATTTN-SRSRPTPMRPTPAPNPSPATARTARTSRL
                Consensus    (201)     TNYDPLVNVPHLEFTTYDPNGNNHKLTFAAYTDDTNSGTESFTSYGKNGK
                                        251                                             300
        GSVIVP00016109001    (226)     NVPAEFTSYGDNSNIIGSTFTAYGQLANSQNDSFKAYGHSSNNPHNNFKS
        VIT_17s0000g08160    (251)     NVPAEFTSYGDNSNIIGSTFTAYGQLANSQNDSFKAYGHSSNNPHNNFKS
    VIT_17s0000g08160_SV1    (247)     SSPATATTPTSSAPLSQLTAN-----------------------------
                Consensus    (251)     NVPAEFTSYGDNSNIIGSTFTAYGQLANSQNDSFKAYGHSSNNPHNNFKS
                                        301                                             350
        GSVIVP00016109001    (276)     YSLGGNAATDTFSNYRDGANVGDDSFQSYARSSNSAEVNFANYGRTFNVG
        VIT_17s0000g08160    (301)     YSLGGNAATDTFSNYRDGANVGDDSFQSYARSSNSAEVNFANYGRTFNVG
    VIT_17s0000g08160_SV1    (268)     --------------------------------------------------
                Consensus    (301)     YSLGGNAATDTFSNYRDGANVGDDSFQSYARSSNSAEVNFANYGRTFNVG
                                        351                                             400
        GSVIVP00016109001    (326)     NDTFKEYGKGSTDSSVGFKIYGLNYTFKDYERKGVTFSQYSRASSNTTAT
        VIT_17s0000g08160    (351)     NDTFKEYGKGSTDSSVGFKIYGLNYTFKDYERKGVTFSQYSRASSNTTAT
    VIT_17s0000g08160_SV1    (268)     --------------------------------------------------
                Consensus    (351)     NDTFKEYGKGSTDSSVGFKIYGLNYTFKDYERKGVTFSQYSRASSNTTAT
                                        401                                             450
        GSVIVP00016109001    (376)     ATTASGISVNRWVEPGKFFRESMLKEGNVMVMPDIRDRMPERSFLPRVIA
        VIT_17s0000g08160    (401)     ATTASGISVNRWVEPGKFFRESMLKEGNVMVMPDIRDRMPERSFLPRVIA
    VIT_17s0000g08160_SV1    (268)     --------------------------------------------------
                Consensus    (401)     ATTASGISVNRWVEPGKFFRESMLKEGNVMVMPDIRDRMPERSFLPRVIA
                                        451                                             500
        GSVIVP00016109001    (426)     SKLPFSSSRLQELKDIFHARDNSTMEHVIANALEECERAPSRGETKRCVG
        VIT_17s0000g08160    (451)     SKLPFSSSRLQELKDIFHARDNSTMEHVIANALEECERAPSRGETKRCVG
    VIT_17s0000g08160_SV1    (268)     --------------------------------------------------
                Consensus    (451)     SKLPFSSSRLQELKDIFHARDNSTMEHVIANALEECERAPSRGETKRCVG
                                        501                                             550
        GSVIVP00016109001    (476)     SVEDMIDFAVSVLGHDVVVRTTETTRGSKQRVMVGEVRGINGGKVTRSVS
        VIT_17s0000g08160    (501)     SVEDMIDFAVSVLGHDVVVRTTETTRGSKQRVMVGEVRGINGGKVTRSVS
    VIT_17s0000g08160_SV1    (268)     --------------------------------------------------
                Consensus    (501)     SVEDMIDFAVSVLGHDVVVRTTETTRGSKQRVMVGEVRGINGGKVTRSVS
                                        551                                             600
        GSVIVP00016109001    (526)     CHQSLYPYLLYYCHSVPKVRVYEVDILDVERKEKMNKGVAICHIDTSAWS
        VIT_17s0000g08160    (551)     CHQSLYPYLLYYCHSVPKVRVYEVDILDVERKEKMNKGVAICHIDTSAWS
    VIT_17s0000g08160_SV1    (268)     --------------------------------------------------
                Consensus    (551)     CHQSLYPYLLYYCHSVPKVRVYEVDILDVERKEKMNKGVAICHIDTSAWS
                                        601                            633
        GSVIVP00016109001    (576)     QSHGAFVALGSSPGLIEVCHWIFENDMTWTTSD
        VIT_17s0000g08160    (601)     QSHGAFVALGSSPGLIEVCHWIFENDMTWTTSD
    VIT_17s0000g08160_SV1    (268)     ---------------------------------
                Consensus    (601)     QSHGAFVALGSSPGLIEVCHWIFENDMTWTTSD
```
