# Supplementary figures and images for: Inspection of the Grapevine BURP Superfamily Highlights an Expansion of RD22 Genes with Distinctive Expression Features in Berry Development and ABA-Mediated Stress Responses
Source: PLoS One. 2014 Oct 16;9(10):e110372. doi: 10.1371/journal.pone.0110372 (PMC4199669; doi:10.1371/journal.pone.0110372)

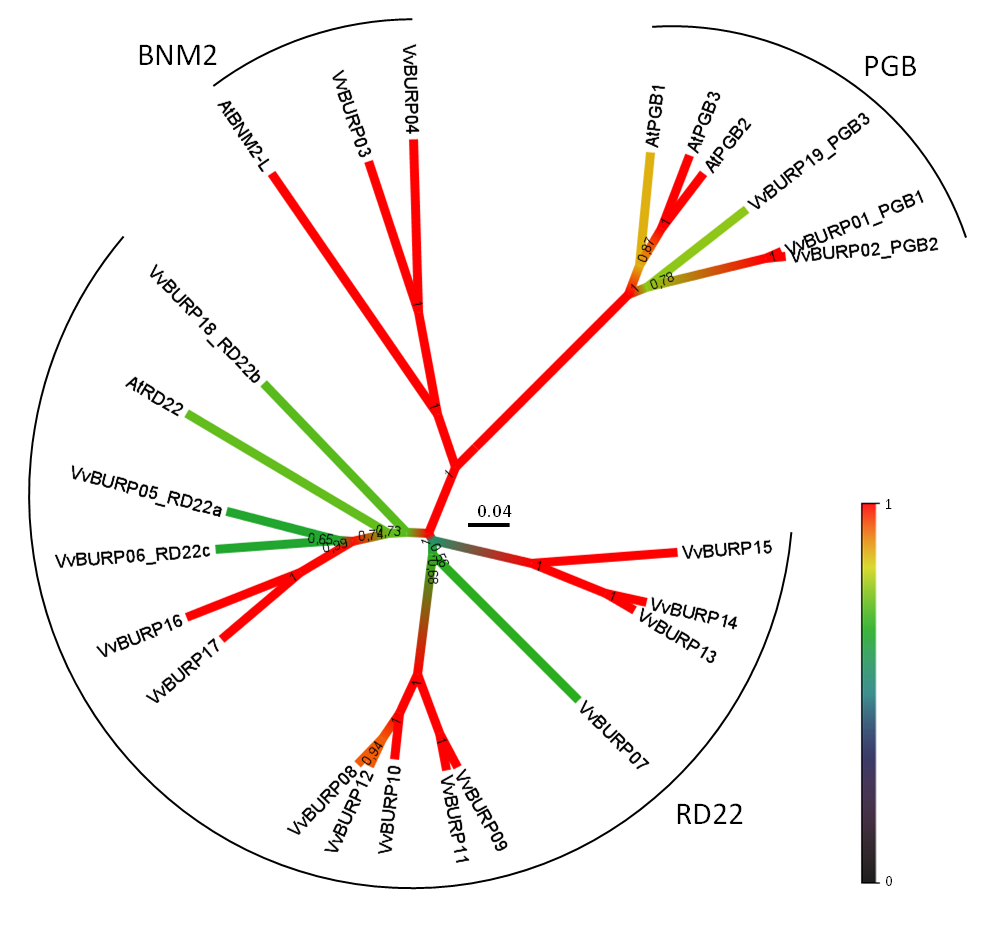

Supplement: Figure S2 — Phylogenetic relationships of BURP homologues from Vitis and Arabidopsis. Colour bar represents bootstrap values for each node. Protein IDs and descriptions for each Arabidopsis gene are found in Table S2. Evolutionary distances are represented as amino acid substitutions per site. (TIF) [file pone.0110372.s002.tif]

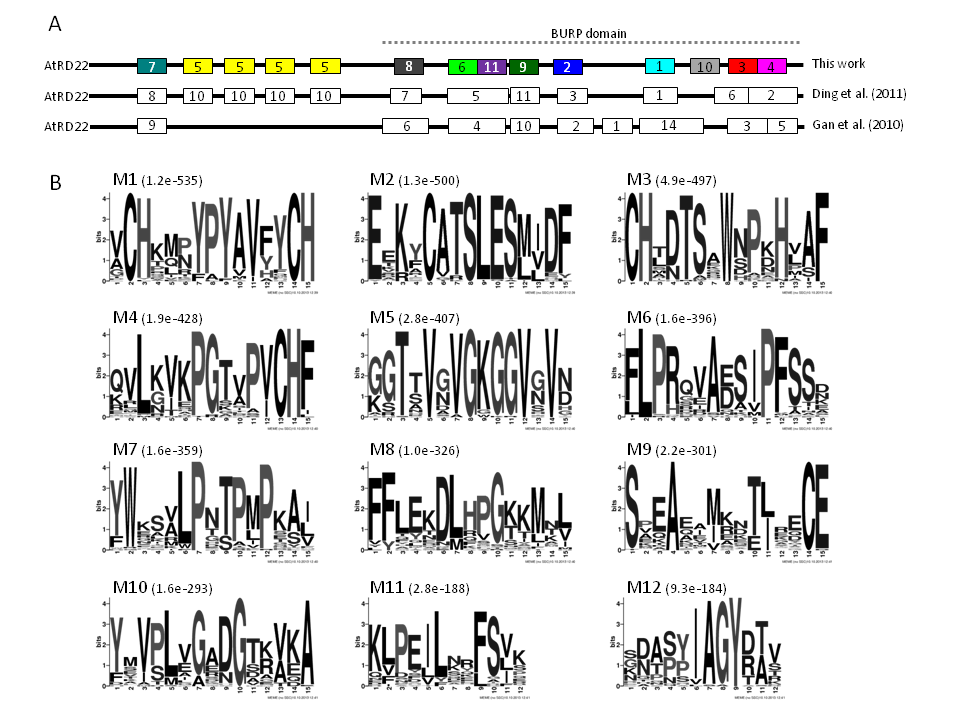

Supplement: Figure S4 — Schematic representation for AtRD22 motifs. A) A comparison of motif identification between this work and those conducted by Ding et al [14] and Gan et al [16]. B) Consensus sequences for each of the motifs found by MEME software. Adjusted p-values for each motif are shown in parenthesis. Motif 12 is only present in VvBURP13, VvBURP14, Gm 11.2 and Gm 12.1. (PNG) [file pone.0110372.s004.png]

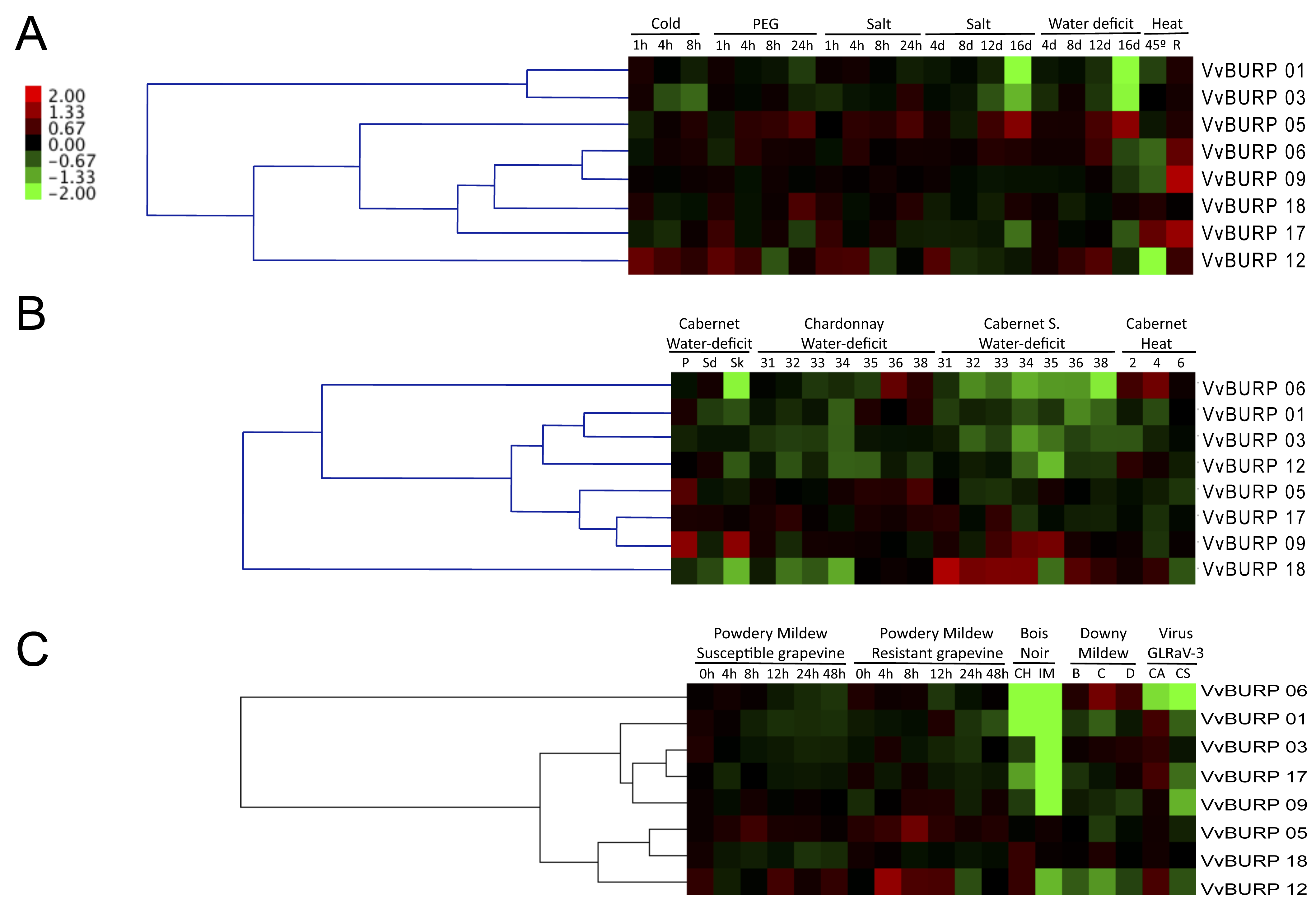

Supplement: Figure S5 — Heatmap clustering of BURP gene expressions for abiotic and biotic stress conditions in grapevine organs, obtained from the Affymetrix Plant Expression database (PLEXdb). A-B) short and long-term abiotic stress conditions in A) vegetative organs and B) berry tissues, C) biotic stress in vegetative tissues. h: hours, d: days, P: pulp, Sd: seed, Sk: skin. R means recovery (25 °C for 5 h) after exposure to 45 °C. Numbers ranging from 31 to 38 represent stages of the Modified Eichhorn-Lorenz system for pericarp samples taken at different developmental stages (35: véraison). CH: cv. Chardonnay, IM: cv. Incrocio Manzoni, B: genotype Rpv1(+)Rpv2(-), C: genotype Rpv1(-)Rpv2(+), D: genotype Rpv1(-)Rpv2(-), CA: cv. Carmenere, CS: cv. Cabernet-Sauvignon. (PNG) [file pone.0110372.s005.png]

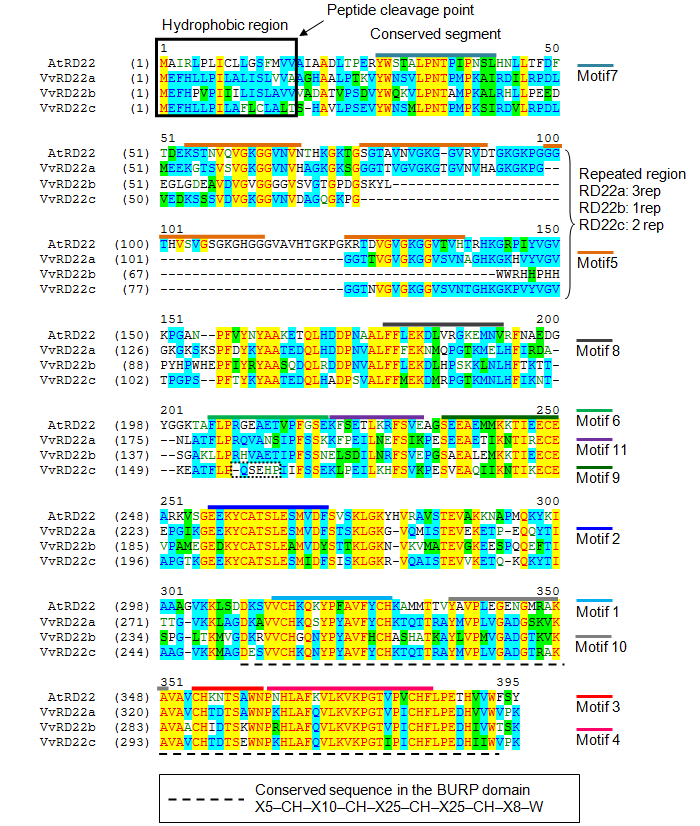

Supplement: Figure S6 — Protein alignment and motifs found in isolated grape RD22 proteins. Residues highlighted in yellow correspond to conserved aminoacids. Orange segments represent the repeated motif 5 identified by MEME. A discontinuous box shows an incomplete motif 6 in RD22c. (PNG) [file pone.0110372.s006.png]
