# Supplementary material for: Inspection of the Grapevine BURP Superfamily Highlights an Expansion of RD22 Genes with Distinctive Expression Features in Berry Development and ABA-Mediated Stress Responses
Source: PLoS One. 2014 Oct 16;9(10):e110372. doi: 10.1371/journal.pone.0110372 (PMC4199669; doi:10.1371/journal.pone.0110372)
Supplement: Figure S3 — Protein alignment between the grape and Arabidopsis BURP domains. Residues highlighted in yellow correspond to conserved aminoacids from all grape and Arabidopsis proteins. Coloured bars correspond to highly conserved segments, which were found by MEME software (Figure S4). (PDF) [file pone.0110372.s003.pdf]

```
AtRD22      (174) ALFFLEKDLVRGKEMNVRFNAEDGYGGKTAFLPRGEAETVPFGSEKFSETLKRFSVEAGSEEAEMMKKTIEECEARKVSGEEK
VvBURP06(c) (126) ALFFMEKDMRPGTKMNLHFIKNTKE---ATFLP-QSEHPIIFSSEKLPEILKHFSVKPESVEAQIIKNTIKECEAPGTKGEEK
VvBURP05(a) (152) ALFFFEKNMQPGTKMELHFIRDANL---ATFLPRQVANSIPFSSKKFPEILNEFSIKPESEEAETIKNTIRECEEPGIKGEEK
VvBURP16    (186) ALFFLEKDMRLGTKMNLDFMKNTNE---ATFLPHQVATSIPFSSDKLPEILDQLSVKPESVEAETIKNTIIDCDRPGIKGEEK
VvBURP17    (137) AIFFLEKDMHPGMKLTLHFTKTTN----ATFLPHQVANSLPFSSDKLAEILDQLSIKPESVEAETIKNTIEECEDPGIKGEEK
VvBURP18(b) (114) ALFFLEKDLHPSKKLNLHFTKTTSG---AKLLPRHVAETIPFSSNEFSDILNRFSVEPGSAEALEMKKTIEECEVPAMEGEDK
VvBURP08    (210) GNFFLETDLHPGKKMKLNLATTTNG---AVFLPHQVAESMPFSSNKLPEILNRFSLKENSAEAEIIKKELEECEEPAMEGEAR
VvBURP10      (1) --------------MKMNLATTTNG---AVFLPHQVAESIPFSSNKLPEILNRFSLKEKSTEAEIIK-ELEECEEPAMEGEAK
VvBURP12     (86) GNFFLETDLHPGKKMKLNLATTTNG---AVFLPHQVAESMPFSSNKLPEILNRFSLKENSAEAEIIKKELEECEEPAMEGEAR
VvBURP09     (49) GNFFLQTDLHPGTKMMLQLPQTRNE---AMFLPRQVADSIPFSSKKLPEILNRLSVKEKSAEAELMKKEEIEECEEDAMDGESR
VvBURP11     (88) GNFFLQTDLHPGTKMVLQLPQTTNE---AMFLPRQVADSIPFSSKKLPEILNRLSVKEKSAEAELMKKEIEKCEEPAMDGESR
VvBURP07    (112) G-YFLEKDLHSTTKMKMHFKKTTNE---ATFLPRQVADSIPFSSDKFPEILNRFSLKQDSEEAEIMKETIQDCEQPALEGYSR
VvBURP15     (38) GNVFLEKDLHPGTKMMVRFTKTSSA---AHFLPQQVAESIPFF------IK--Q--------ATRNFESVLKCEKPAIEGEEK
VvBURP13    (311) VNVFLEKDLHPGTKMAVRFTKTSSA---AHFLPHQVAESIPFSSNKLPEILKRFSVKENSAEAQIIQKTIKQCETPAIVGEVK
VvBURP14    (326) VNVFLEKDLHPGTKMAVRFTKTSSA---AHFLPHQVAESIPFSSNKLPEILKRFSVKENSAEAQMIQKTIKQCETPAIVGEVK
AtBNM2-L     (56) YMYFTLNDLKLGTKLLIYFYKNDLQK-LPPLLTRQQADLIPFTKSKLDFLLDHFSITKDSPQGKAIKETLGHCDAKAIEGEHK
VvBURP04     (56) RIFFTITELKVGKRIPVYFSKRDPAT-SPHLLPREEVESIPFSSAQLPYLLQFFGFSQGSPQAIAMENTLRHCETEPIEGETK
VvBURP03    (108) VVFFTMKDLKVGKTMPIYFAKTDPAS-SPRMLPKEEADSIPFSFAQLPHLLEFFSFSQGSPQARAMENTLRECELKPIKGETK
AtPGB1      (407) GKFFREAMLKEGTLMQMPDIKDKMP--KRTFLPRNIVKNLPFSSSTIGEIWRVFGAGENSSMAGIISSAVSECERPASHGETK
AtPGB2      (409) GKFFRESSLKEGTVIPMPDIKDKMP--KRSFLPRSIITKLPFSTSKLGEIKRIFHAVENSTMGGIITDAVTECERPPSVGETK
AtPGB3      (405) GKFFRESMLKEGTLIWMPDIKDKMP--KRSFLPRSIVSKLPFSTSKIAEIKRVFHANDNSTMEGIITDAVRECERPPTVSETK
VvBURP02    (407) GKFFRESMLKKGTVMPMPDIRDKMP--KRSFLPRSISSKLPFSTSKLEEMKKIFHAADNSSMEHMFTEALDDCERAPSKGETR
VvBURP01    (416) GKFFRESMLKKGTVMPMPDIRDKMP--KRSFLPRTISSKFPFSTSKLEEMKKIFHAADNSSMEHMFTEALDDCERAPSKGETK
VvBURP19    (416) GKFFRESMLKEGNVMVMPDIRDRMP--ERSFLPRVIASKLPFSSSRLQELKDIFHARDNSTMEHVIANALEECERAPSRGETK

AtRD22      (257) YCATSLESMVDFSVSKLGK-YHVRAVSTEVAKKNAP-M---QKYKIAAAGVKKLSDDKSVVCHKQKYPFAVFYCHKAMMTTVY
VvBURP06(c) (205) YCATSLESMIDFSISKLG--KRVQAISTEVVKETQK------QKYTIAAGVKKMAGDESVVCHKQNYPYAVFYCHKTQTTRAY
VvBURP05(a) (232) YCATSLESMVDFSTSKLG--KGVQVISTEVEKETPE------QQYTITTGVKKLAGDKAVVCHKQSYPYAVFYCHKTQTTRAY
VvBURP16    (266) YCATSLESMIDFSTSKLGN-KGVKAVSTEVENKSQ-------TLYRIAAGVEKMGGDVSVVCHKMEYAYAVFYCHKIAATRAY
VvBURP17    (216) YCATSLESMIDFSTSKLGN-KGVKAVSTEAENKSQ-------MKYRIAAGLEKMGGDFSVVCHKMNYPYAVFYCHKIQATRAY
VvBURP18(b) (194) YCATSLEAMVDYSTSKLG--KNVKVMATEVGKEESPQ-----QEFTISPGLTKMVGDKRVVCHGQNYPYAVFHCHASHATKAY
VvBURP08    (290) YCATSLQSLIHFSTSKLG--RNVNVLTNEVKTGSQ--------EYEFGVGMKRVA-DKSVVCHKMNYPYAVFYCHTFTKTRTY
VvBURP10     (66) YCTTSLESLIDFSTSKLG--RNVNVQANEVKTG--------SQEYEFGVGMEKLA-DKSVVCYKMNYPYAVFYCHTFTKTRTY
VvBURP12    (166) YCATSLQSLIHFSTSKLG--RNVNVLTNEVKTG--------SQEYEFGVGMKRVA-DKSVVCHKMNYPYAVFYCHTFTKTRTY
VvBURP09    (129) FCATSLESLIDFSTSKLG--RNVNVLTNEVKTGSQ--------EYEFGVGMKRVA-DKSVVCHKMNYPYAVFYCHTFTKTRTY
VvBURP11    (168) FCATSLESLIDFSTSKLG--RNVNVLTNEVRMG--------SQEFEFGVRMKKVA-DKSVVCHKMNYPYAVFYCHTFAKTRTY
VvBURP07    (191) FCATSLESLIDFSISKLG--KNIKLISNGVEMG--------SQEYELGVGVKVVA-DKSVVCHKQKYPYAVFYCHATHKTRVY
VvBURP15    (102) YCARSLESLIDFSTSKLG--KNIRALPNVVEGEIQ--------EYKFGEGAKMLG-EKSVVCHQLNYPYAVALCHAFHMTKIY
VvBURP13    (391) YCARSLESLIDFSTSRLG--KNIRVLSNEVEADIQ--------EYKFGEGVKMVG-EKSVVCHQLNYPYAVAFCHTLHMTKIY
VvBURP14    (406) YCARSLESLIDFSTSRLG--KNIRVLSNEVEADIQ--------EYKFGEGVKMVG-EKSVVCHQLNYPYAVAFCHTLHMTKIY
AtBNM2-L    (138) FCGTSLESLIDLVKKTMGYNVDLKVMTTKVMVPAQNSISYALHNYTFVEAPKELVGIKMLGCHRMPYPYAVYYCHGHKGGSRV
VvBURP04    (138) SCVTSLESMLDFSRKIFGL-KASFEVISTKLGEKTTSL---LQNYTILKLPKPISAPKMVACHTLPYPYAVFYCHFQEGENKV
VvBURP03    (190) FCATSLESLLDFVHSIFGL-ESHFQVLTTSYLTKSSTL---FQNYTFLEVPTEIPAPKMVACHTMPYPYAIFYCHSQASENKV
AtPGB1      (488) RCVGSAEDMIDFATSVLGR-GVVVRTTENVVGSKKK------VVIGKVNGINGGDVTRAVSCHQSLYPYLLYYCHSVPRVRVY
AtPGB2      (490) RCVGSAEDMIDFATSVLGR-SVVLRTTENVAGSKEK------VVIGKVNGINGGKLTKAVSCHQSLYPYLLYYCHSVPKVRVY
AtPGB3      (486) RCVGSAEDMIDFATSVLGR-SVVLRTTESVAGSKEK------VMIGKVNGINGGRVTKSVSCHQSLYPYLLYYCHSVPKVRVY
VvBURP02    (488) RCVPSIEDMIDFATTVLGR-NVVVRTTQSVEGSKQN------VMIGSVKGINGGQVTKSVSCHQSLFPYLLYYCHFVPKVRVY
VvBURP01    (497) RCVPSIEDMIDFATTVLGR-NVVVRTTQSVEGSKQN------LMIGSVKGINGGQVTKSVSCHQSLFPYLLYYCHSVPKVRVY
VvBURP19    (497) RCVGSVEDMIDFAVSVLGH-DVVVRTTETTRGSKQR------VMVGEVRGINGGKVTRSVSCHQSLYPYLLYYCHSVPKVRVY

AtRD22      (335) AVPLEG--ENGMRAKAVAVCHKNTSAWNPNHLAFKVLKVKPGTVPVCHFLPETHVVWFSY
VvBURP06(c) (280) MVPLVG--ADGTRAKAVAVCHTDTSEWNPKHLAFQVLKVKPGTIPICHFLPEDHIIWVPK
VvBURP05(a) (307) MVPLVG--ADGSKVKAVAVCHTDTSAWNPKHLAFQVLKVKPGTVPICHFLPEDHVVWVPK
VvBURP16    (341) MVPLVG--RDGAKAKAVALCHTNTKEWNPKHLAFQLLKVKPG-VPICHFLTQDQIIWVVS
VvBURP17    (291) MVPLVG--RDGTKAKAVAVCHANTMEWNPNHLAFQLLKVKPGTAPICHFLPEDHVVWVAK
VvBURP18(b) (270) LVPMVG--ADGTKVKAVAACHIDPSKWNPRHLAFQVLKVKPGTVPICHFLPEDHIVWTSK
VvBURP08    (362) MIPLVG--ADGSKAKAMAACHSDTSAWHPQHVAFQVLKIKPGTVAVCHFLHNNAMVWIPK
VvBURP10    (138) MIPLVG--ADGSKAKAMSACHSDRSAWHPKHVAFKVLNVKPGTVPVCHFVHNNAMVWIPK
VvBURP12    (238) MIPLVG--ADGSKAKAMAACHSDTSAWHPKHVAFKVLNVKPGTVPICHFVHNNAMVWIPK
VvBURP09    (201) MIPLVG--ADGSKAKAMAACHSDTSAWHPKHVAFKVLNVKPGTVPICHFVHNNAMVWIPK
VvBURP11    (240) MIPLVG--DDGSKAKAMAACHSDTSAWHPQHVAFQVLKIKPGTVPICHFLHNNAMVWIPK
VvBURP07    (263) TLPFVGT-EDGTKVEVVASCHIDTSAWNPKHAAFQVLKVKPGTVPVCHFLPRDDLIWVPK
VvBURP15    (174) KVPLVG--ADGTRVQALAVCHEDTSIWDPNALAFQVLKVKPGTWPICHFLPNGHFVWVPN
VvBURP13    (463) MVPLVG--ADGTGVEAVAVCHRDTSTWDPKALVFQSLKVKPGTLPICHFLPNGHTVWVPK
VvBURP14    (478) MVPSVG--ADGTGVEAVAVCHRDTSTWDPKALVFQRLKVKPGTLPICHFLPNGHIVWVPK
AtBNM2-L    (221) FEVNLVTDDGRQRVVGPAVCHMDTSTWDADHVAFKVLKMEPRSAPVCHFFPLDNIVWVTK
VvBURP04    (217) FEVSLGG-ENGDRVEAVAVCHMDTSQWNQDHVSFRLLGVQPGASPVCHFFPADNLIWVPS
VvBURP03    (269) FKVSLEG-QNGDRVEAFAVCHLDTSAWSRDHVSFRVLGIEPGTSPVCHFFPASNLIWVPR
AtPGB1      (564) ETDLLDPKSLEKINHGVAICHIDTSAWSPSHGAFLALGSGPGQIEVCHWIFENDMTWNII
AtPGB2      (566) EADLLELNSKKKINHGIAICHMDTSSWGPSHGAFLALGSKPGRIEVCHWIFENDMNWAIA
AtPGB3      (562) ESDLLDPKSKAKINHGIAICHMDTSAWGANHGAFMLLGSRPGQIEVCHWIFENDMNWAIA
VvBURP02    (564) EADLLDPKTKANINHGVAICHLDTSDWSAGHGAFVALGSGPGRIEVCHWIFENDMTWTIV
VvBURP01    (573) EADLLDPKTKANVNHGVAICHLDTSDWSAGHGAFVALGSGPGRIEVCHWIFENDMTWTIA
VvBURP19    (573) EVDILDVERKEKMNKGVAICHIDTSAWSQSHGAFVALGSSPGLIEVCHWIFENDMTWTTS
```
